# BACE1 regulates expression of Clusterin in astrocytes for enhancing clearance of β-amyloid peptides

**DOI:** 10.1186/s13024-023-00611-w

**Published:** 2023-05-04

**Authors:** John Zhou, Neeraj Singh, James Galske, Jacob Hudobenko, Xiangyou Hu, Riqiang Yan

**Affiliations:** 1 Department of Neuroscience, UConn Health, 263 Farmington Avenue, Farmington, CT 06030-3401 USA; 2 Department of Molecular Medicine, Cleveland Clinic Lerner College of Medicine, Case Western Reserve University, Cleveland, OH 44195 United States; 3 Department of Neuroscience, Lerner Research Institute, Cleveland Clinic, Cleveland, OH 44195 United States

**Keywords:** BACE1, Amyloid plaques, Reactive astrocytes, Clusterin, CXCL14, Insulin receptor, Aβ clearance

## Abstract

**Background:**

Abnormal accumulation of amyloid beta peptide (Aβ) in the brain induces a cascade of pathological changes in Alzheimer’s disease (AD), and inhibiting BACE1, which is required for Aβ generation, is therefore being explored for the treatment of AD by reducing Aβ accumulation. As *Bace1* knockout mice exhibit increased number of reactive astrocytes and AD brains have reactive astrocytes that surround amyloid plaques, we investigated the role of BACE1 in astrocytes and determined whether BACE1 regulates astrocytic functions.

**Methods:**

We conducted unbiased single cell RNA-seq (scRNA-seq) using purified astrocytes from *Bace1* KO mice and wild type control littermates. Similar scRNA-seq was also conducted using AD mice with conditional deletion of *Bace1* in the adult stage (*5xFAD;Bace1*^*fl/fl*^*;UBC-creER* compared to *5xFAD;Bace1*^*fl/fl*^ controls). We compared the transcriptomes of astrocyte and reactive astrocyte clusters and identified several differentially expressed genes, which were further validated using *Bace1* KO astrocyte cultures. Mice with astrocyte-specific *Bace1* knockout in 5xFAD background were used to compare amyloid deposition. Mechanistic studies using cultured astrocytes were used to identify BACE1 substrates for changes in gene expression and signaling activity.

**Results:**

Among altered genes, Clusterin (*Clu*) and *Cxcl14* were significantly upregulated and validated by measuring protein levels. Moreover, BACE1 deficiency enhanced both astrocytic Aβ uptake and degradation, and this effect was significantly attenuated by siRNA knockdown of *Clu*. Mechanistic study suggests that BACE1 deficiency abolishes cleavage of astrocytic insulin receptors (IR), and this may enhance expression of *Clu* and *Cxcl14*. Acutely isolated astrocytes from astrocyte-specific knockout of *Bace1* mice (*Bace1 *^*fl/fl*^*;Gfap-cre*) show similar increases in CLU and IR. Furthermore, astrocyte-specific knockout of *Bace1* in a 5xFAD background resulted in a significant attenuation in cortical Aβ plaque load through enhanced clearance.

**Conclusion:**

Together, our study suggests that BACE1 in astrocytes regulates expression of *Clu* and *Cxcl14*, likely via the control of insulin receptor pathway, and inhibition of astrocytic BACE1 is a potential alternative strategy for enhancing Aβ clearance.

**Supplementary Information:**

The online version contains supplementary material available at 10.1186/s13024-023-00611-w.

## Background

Typical clinical symptoms of Alzheimer’s disease (AD) are gradual loss of memory and cognitive ability, likely resulting from amyloid deposition and neurofibrillary tangles in the patients’ brains [1–3]. BACE1 is identified as Alzheimer’s β-secretase for initiating production of β-amyloid peptides (Aβ) from amyloid precursor protein (APP), and oligomeric or aggregated Aβ is viewed as the major pathogenic protein causing amyloid deposition and senile plaques [4]. Inhibition of BACE1 is, therefore, pursued for treating AD patients [5]. As demonstrated in clinical trials, BACE1 inhibition is effective for reducing brain Aβ levels [6]. However, BACE1 inhibitors have failed in the trials due to lack of cognitive benefit [7–9]. This is in line with reports that reveal critical roles of neuronal BACE1 in the control of synaptic transmission [10], proper organization of hippocampal mossy fiber infrapyramidal bundle [11], and regulation of synaptic plasticity such as long term potentiation (LTP) from Schaffer collateral-CA1 synapses and in mossy fiber-CA3 synapses [12–16]. *Bace1* KO mice also exhibit deficits in myelination and neurogenesis, as well as schizophrenia-like behaviors and seizures [17].

Considering the fact that abnormal accumulation of Aβ leads to amyloid deposition, which is associated with pathological events including the intraneuronal tau hyperphosphorylation and formation of neurofibrillary tangles [18, 19], it becomes important to understand how to properly inhibit BACE1 activity and to avoid neuronal side effects. We have previously shown that deletion of *Bace1* in the adult mice removes pre-existing amyloid plaques [15], implying the potential upregulation of clearance machinery, which is most likely mediated by glia cells. BACE1 is also expressed by astrocytes [20, 21], and germline *Bace1* deficiency induces astrogenesis during mouse early development [22]. The role of BACE1 in astrocyte function in the context of AD however remains to be explored. Hence, we aimed to determine the role of BACE1 in astrocytes by utilizing the generated *Bace1* global and conditional knockout mice.

We purified astrocytes from 2-month-old *Bace1* KO mouse brains and conducted single cell sequencing (scRNA-seq). Deletion of *Bace1* in astrocytes increased the percentage of reactive astrocytes when compared to wild type (WT) littermates. Genes related to Aβ clearance, including *Clusterin* (*Clu*), were significantly increased in *Bace1* deficient astrocytes. We confirmed that this upregulation of *Clu* in *Bace1*-null astrocytes also resulted in an increase at the protein level in astrocytes from global and astrocyte-specific deletion of BACE1. Downregulation of CLU partially reversed the effect of *Bace1* deletion on astrocytic clearance of Aβ. Mechanistically, we show that astrocytic BACE1 deficiency results in an increase in insulin receptor (IR) and downstream P38 and ERK1/2 signaling pathways, which may regulate transcription of the Aβ clearance-related genes. Astrocyte specific-knockout of *Bace1* (*Bace1*^*fl/fl*^*;Gfap-cre*) resulted in similar increases in both CLU and IR. Furthermore, astrocyte specific-knockout of *Bace1* crossed with 5xFAD (*5xFAD;Bace1*^*fl/fl*^*;Gfap-cre*) resulted in a significant attenuation of total cortical Aβ plaque load without affecting Aβ generation. Together, this suggests that astrocytic inhibition of BACE1 may contribute to a enhanced amyloid clearance and might provide an alternative therapeutic pathway that avoids off-target effects associated with significant inhibition of neuronal BACE1.

## Results

### Deletion of *Bace1* in astrocytes alters transcriptomic profiles of reactive population

**Fig. 1:**
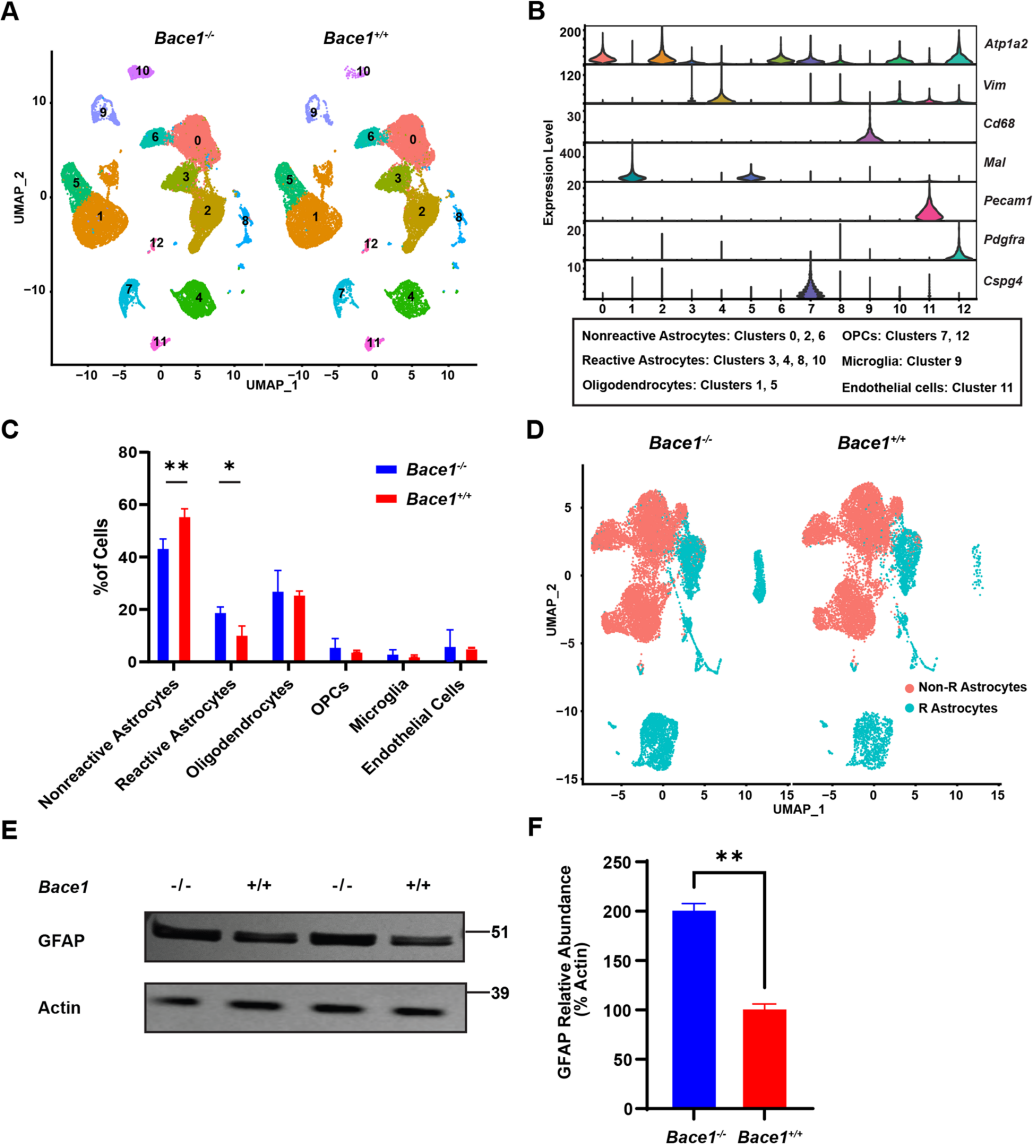
BACE1 deficiency enhances reactive astrocyte population. **A** Total Uniform manifold approximation and projection (UMAP) clustering of ACSA2 + positive cells from derived from 2-month old *Bace1*-null and WT mice via single-cell RNA-seq, *N* = 3 samples, ~ 17,000 cells per genotype. Clusters are labeled by each cell type. **B** Violin plot representation of log2 fold change gene expression of known cell type gene markers and correlated with labeled clusters. **C** Proportion of indicated cell types. Comparison of average proportion of each cell types from each samples genotype (* *p*-value < 0.05, ** *p*-value < 0.01). Two-way ANOVA with Sidak multiple comparison post-test. **D** UMAP of only astrocyte clusters after removing other cell types. R Astrocytes refer reactive astrocytes while Non-R Astrocytes refer to non-reactive astrocytes. **E** Western blot of primary astrocytes cultures lysates derived from *Bace1*-null and WT mice to confirm increase in reactive astrocytes from *Bace1*-null mice. GFAP antibody was used for detecting GFAP levels, while actin was for loading controls. **F** GFAP levels were quantified based on Western blot band intensity normalized to actin (*N* = 3, ** *p*-value < 0.01, Student t-test)

To understand the role of BACE1 in astrocytes, we enriched astrocytic cell population from 3 brains of wild-type (WT) and *Bace1* KO mice at 2-months of age using astrocyte-cell specific antigen 2 (ACSA2) immunomagnetic beads. Purified astrocytes were subjected to 10 × Genomic single cell RNA sequencing and initial read count analysis by Cell Ranger (10 × Genomics). Overall gene number, read counts, and mitochondrial RNA appeared to be similar between all samples (Supplemental Figure S1A). Approximately 17,000 cells were identified for each genotype. Using Seurat R package (Version 4, Satija lab), we analyzed the sequencing results and clustered these cells into UMAP-defined clusters (Fig. 1A). Different cell clusters were arranged based on the unique gene signature as previously described [23, 24]: 1) major populations of non-reactive astrocytes [indicated by high *Atp1b2* expression and low *Vimentin* (*Vim*) expression], 2) reactive astrocytes (high *Atp1b2* and high *Vim*), 3) microglia [*Cluster of differentiation 68* (*CD68*)], 4) oligodendrocytes [*Myelin and lymphocyte protein* (*Mal*)], 5) oligodendrocyte precursor cells (OPC) [*Platelet-derived growth factor receptor A 2* (*Pdgfr2*) or *Chondroitin sulfate proteoglycan 4* (*CSPG4*)], and 6) endothelial cells [Platelet endothelial cell adhesion molecule (*PECAM1)*] (Fig. 1B). From UMAP-defined clusters, it appeared that Cluster 0, 2, and 6 were non-reactive astrocytes while Clusters 3, 4, 8, and 10 were reactive astrocytes (Fig. 1B and Supplemental Figure S1B). As expected, astrocytes represented the majority of cells, and approximately 20% of remaining cells were identified as oligodendrocytes (ochre; Clusters 1 and 5), and OPCs (teal; Cluster 7 and 12). Although in low abundance, microglia (in cluster 9 in blue) and endothelial cells (cluster 7 in fuchsia) were present (Fig. S1B).

After using known brain cell type markers to filter out non-astrocyte cells [23, 25], we re-clustered and compared gene profiles derived from *Bace1*-null and WT astrocytes. Quantification showed that reactive astrocytes comprised approximately 20% of total cells in *Bace1*^*−/−*^ brains compared to only ~ 10% reactive astrocytes in *Bace1*^+*/*+^ controls (Fig. 1C). Correspondingly, the proportion of non-reactive astrocytes in WT controls was greater, while other cell populations were not significantly altered under this immune-panning condition (Fig. 1C). Furthermore, BACE1 deficiency visibly increased numbers of reactive astrocytes (labeled as R Astrocytes in teal, Fig. 1D).

Consistent with this unbiased result, we found that GFAP protein levels in lysates from BACE1 deficient primary astrocyte cultures were significantly higher compared to WT controls (Fig. 1E and F), further indicating that BACE1 deficiency promotes astrocytes in the reactive states.

**Fig. 2:**
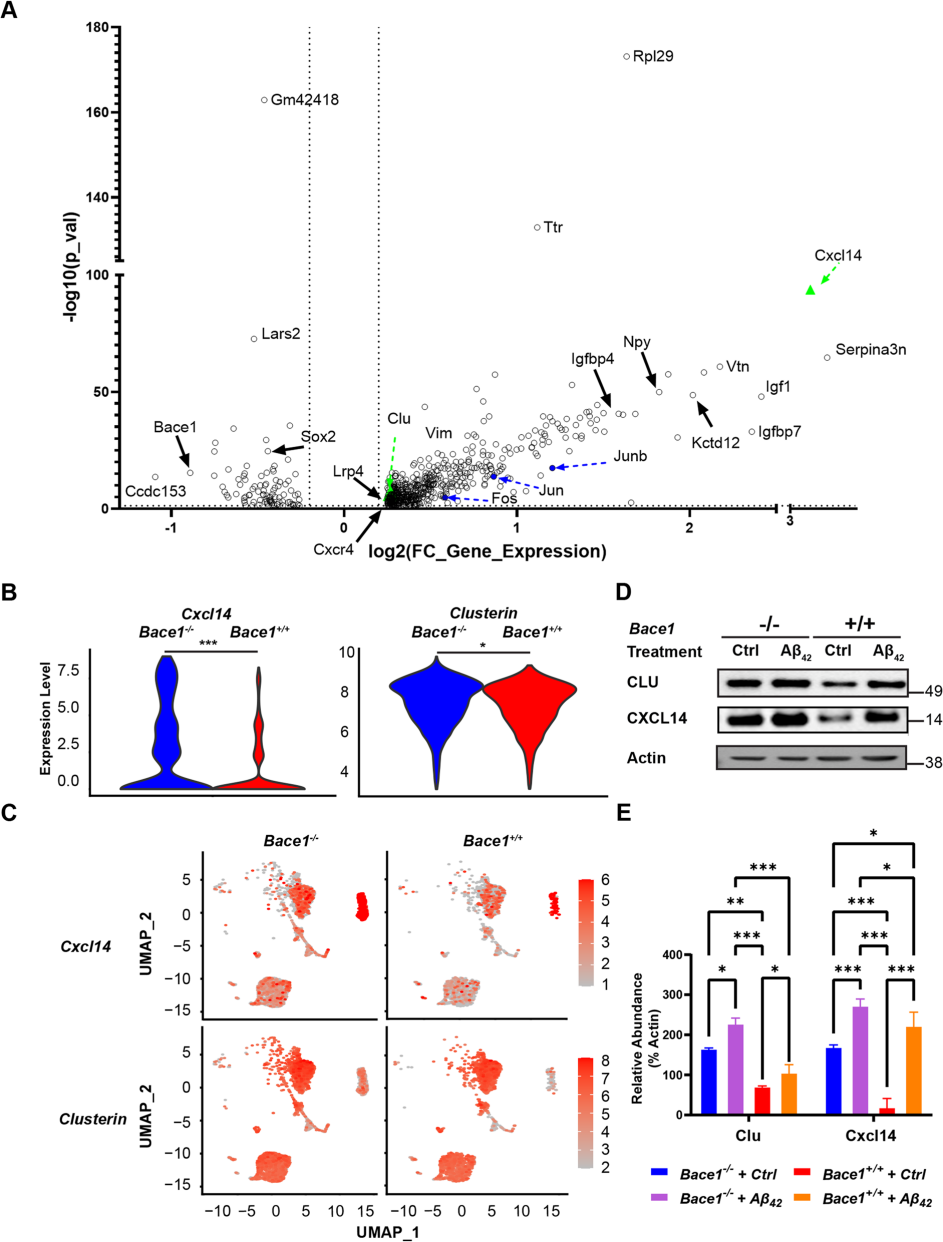
Germline BACE1 deficiency results in unique reactive astrocyte transcriptomes. **A** Top differentially expressed genes comparing *Bace1*-null to WT reactive astrocyte transcriptomes expressed as a volcano plot of log2 fold change value gene expression and –log10(p-value), Wilcoxon ranked sum test was used to calculate p-values. Dotted lines indicate DEG cut-offs for |log2(fold change)|> 0.2 and -log10 (*p*-value) of 1.3, corresponding to *p*-value < 0.05. Green triangle highlights elevated expression of *Clu* and *Cxcl14*. Blue dots highlight genes that are members of the AP-1 transcription machinery (**B**) Violin plot of genes of interest, *Clu*, and *Cxcl14*, expression comparing *Bace1*-null and WT reactive astrocyte clusters (* *p*-value < 0.05, *** *p*-value < 0.001; Wilcoxon ranked sum test was used to calculate p-value). **C** Distribution of cells highly expression *Clu* and *Cxcl14*. Scale indicates gene expression levels. **D** Western blotting of genes of interest – *Clu* and *Cxcl14 *– in primary astrocyte culture lysates with or without 2 μM of aggregated Aβ_42_ treatment. **E** Quantification of Western blot band intensity normalized to actin with or without Aβ_42_ treatment (*N* = 3, * *p*-value < 0.05, ** *p*-value < 0. *** *p*-value < 0.001; One-way ANOVA with Sidak post-test)

Comparing the transcriptomes of pan reactive astrocytes from *Bace1* KO and WT mice using Seurat V4, we identified 37,658 unique gene signatures, with about 610 significantly differentially expressed genes (DEGs) (adjusted p-value < 0.05, log2FC > 0.2; Supplemental Table 1). A volcano plot revealed many significant DEGs (Fig. 2A). Among these genes, we found several genes known to have roles in synaptic maintenance, clearance of β-amyloid peptides (Aβ), glutamate homeostasis, metabolism, hippocampal synaptogenesis, insulin and insulin growth factor signaling and AP-1 transcription factor family (Table 1 and Supplemental Table 1). Expectedly, *Bace1* from *Bace1* KO reactive astrocytes was also significantly reduced.

Genes thought to regulate clearance of Aβ, marked in green triangles, received our particular attention. We showed that *Clu* and *C-X-C Motif Chemokine Ligand 14* (*Cxcl14*) expression levels were significantly elevated in *Bace1*-null reactive astrocyte gene clusters (Fig. 2B and C). These changes were then further validated on the protein level. Western blot analysis showed that both CLU and CXCL14 protein levels were increased in *Bace1*-null primary astrocytes, both basally and when stimulated with Aβ, compared to WT astrocytes (Fig. 2D-E).

### Deletion of *Bace1* in 5xFAD mice enhances expression of Clu and Cxcl14

**Fig. 3:**
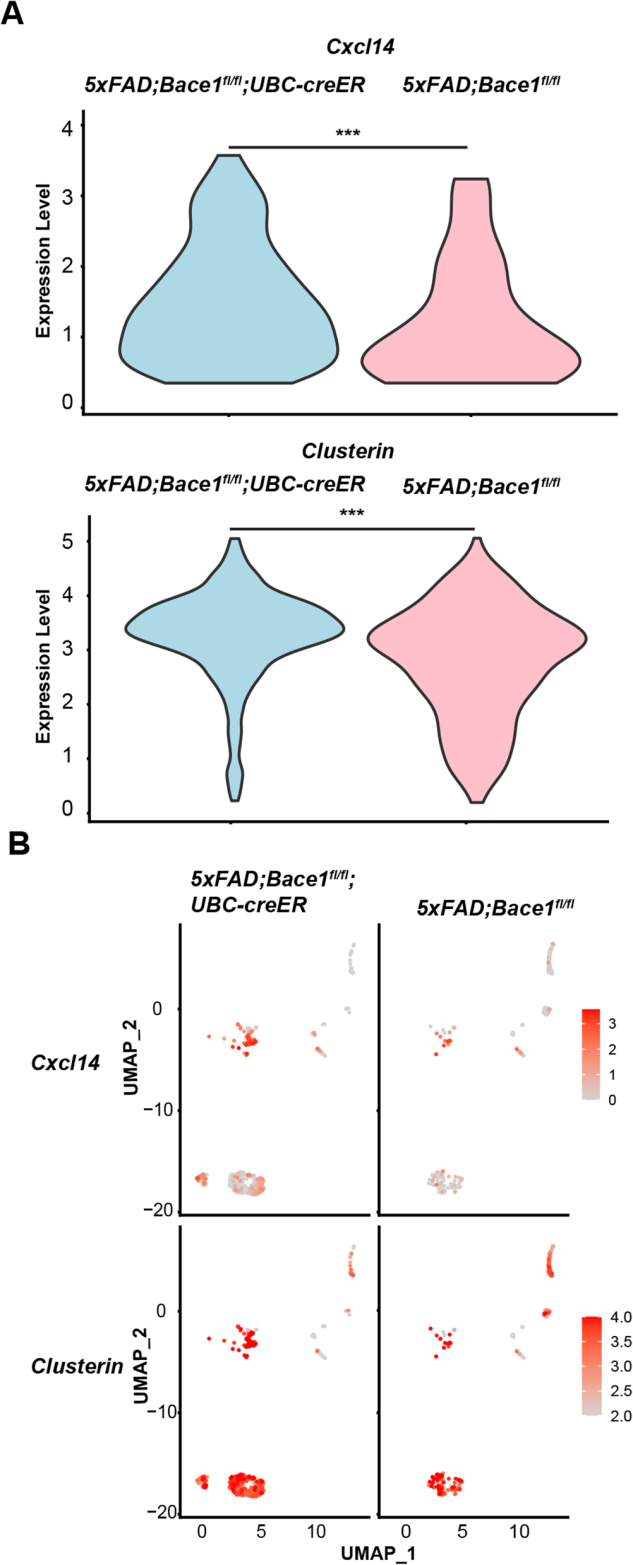
Bace1 deficiency in adult mice increases *Clu* and *Cxcl14* gene expression in reactive astrocytes. **A** Transcriptomic analysis was performed on single-cell RNA-seq of astrocytes sorted from 14-month-old 5*xFAD;Bace1*^*fl/fl*^ and *5xFAD;Bace1*^*fl/fl*^*;UBC-creER* mice (*N* = 3, per genotype). Violin plot of genes showed high expression of *Clu* and *Cxcl14* (log2 fold change expression) in *5xFAD;Bace1*^*fl/fl*^*; UBC-creER* compared *5xFAD;Bace1*^*fl/fl*^ reactive astrocyte clusters. **B** Distribution of cells highly expressing *Clu* and *Cxcl14* with indicated log2 fold change scaling based on UMAP clustering of astrocytes

As previously mentioned, conditional knockout of BACE1 in the adult reverses previously formed amyloid plaques in adult *5xFAD;Bace1*^*fl/fl*^*; UBC-creER* compared to non-BACE1 deleted *5xFAD;Bace1*^*fl/fl*^* adult mice* [15]. To understand the role that adult BACE1 deficiency might play on astrocyte transcriptomes in adult mice with the 5xFAD background, we compared scRNA-Seq of astrocytes from 14-month old *5xFAD;Bace1*^fl/fl^*; UBC-creER* and *5xFAD;Bace1*^*fl/fl*^ mice. Overall gene number, read counts, and mitochondrial RNA appeared to be similar between all samples (See Supplemental Figure S2). About 7,000 astrocytes were recovered from these mice after magnetic activated cell sorting (MACS) by ACSA2 immunomagnetic beads and cell type filtration. When comparing reactive astrocyte population transcriptomes from *5xFAD;Bace1fl/fl; UBC-creER *and *5xFAD;Bace1*^*fl/fl*^ mice, 1,873 DEGs (*P* < 0.05) were noted (Supplemental Table 2). Among these DEGs, insulin degrading enzyme (IDE), klotho (Kl) and lysosomal associated membrane protein 2 (LAMP2) were upregulated in the *5xFAD;Bace1*^*fl/fl*^*; UBC-creER* compared to *5xFAD;Bace1*^*fl/fl*^ mice. Furthermore, we once again found significantly elevated *Clu* and *Cxcl14* gene expression in the reactive astrocyte population (Fig. 3A), and an increase in the number of *Clu*^high^ and C*xcl14*^high^ astrocytes in the case of *5xFAD;Bace1*^*fl/fl*^*; UBC-creER* mice (Fig. 3B).

Our data suggest that both germline and adult BACE1 deficiency increases a group of astrocytes that commonly express more *Cxcl14* and *Clu*. These *Clu*^high^ and *Cxcl14*^high^ reactive astrocytes are likely capable of clearing more β-amyloid peptides (Aβ) as higher levels of CLU are known to increase Aβ clearance [26, 27].

### BACE1 deficiency increases uptake and degradation of Aβ by astrocytes

**Fig. 4:**
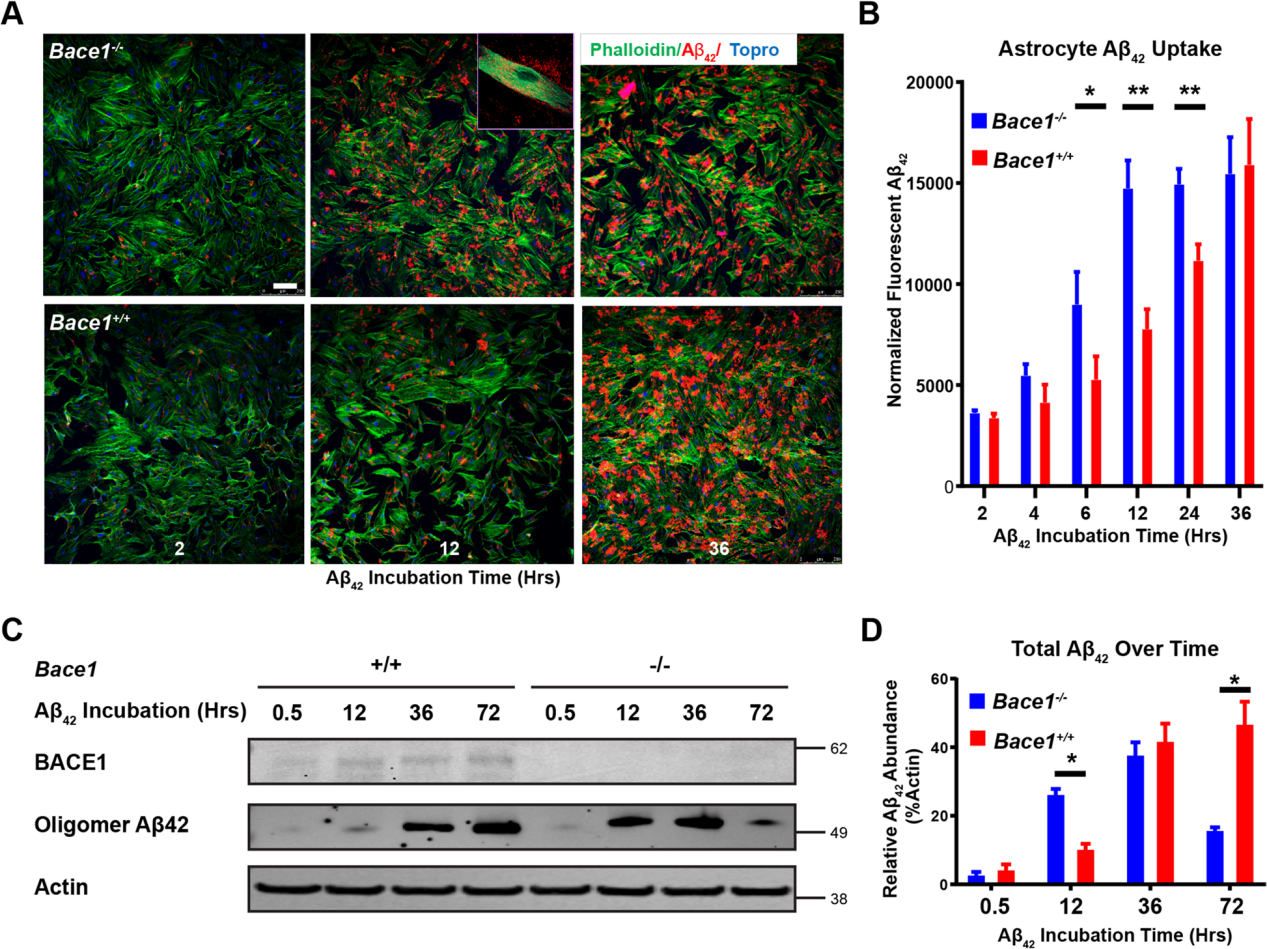
BACE1 deficiency enhances astrocytic clearance of Aβ in vitro. **A** Confocal imaging of astrocyte primary cultures from BACE1-null and WT perinatal mice pups and treated with 2 μM of Aβ_42_ tagged with fluorescent Hilyte-555 for indicated incubation times. Stained with phalloidin (green) to mark F-actin and ToPro3 (blue) to mark nuclei. Scale bars represents 10 μm. Inset shows magnified image of Aβ_42_ within phalloidin marked astrocyte boundaries. **B** Quantification of Aβ_42_ integrated fluorescence within phalloidin marked astrocyte boundaries and normalized to number of nuclei and astrocyte area (N = 6, * *p*-value < 0.05, ** *p*-value < 0.01; One way ANOVA, with Tukey post testing comparing between time points. **C** Western blot of astrocyte primary culture lysates from indicated genotypes and treated with 2 μM of aggregated Aβ_42_ for the indicated incubation times. Images indicate major bands for BACE1, Aβ_42_ (human, oligomeric), and actin. **D** Quantification of Western blot Aβ_42_ band intensity normalized to actin (*N* = 3, * *p*-value < 0.05, One-way ANOVA, with Tukey post testing comparing between samples)

Since BACE1 deficiency altered astrocytic transcriptomic profiles, we then asked whether astrocytes with BACE1 deficiency would enhance Aβ clearance, which might contribute to the reversal of pre-formed amyloid plaques in 5xFAD mice with deletion of *Bace1* in the adult as previously reported [15]. We cultured primary astrocytes from WT and *Bace1*-null mice, and then treated these cultured astrocytes with aggregated oligomeric human Aβ_42_ according to the published procedure [28]. We found that BACE1 deficient astrocytes had visibly increased uptake of HiLyte Fluor-555-labeled Aβ_42_ (Anaspec, Fremont, CA) after 12 hrs incubation compared to WT controls (Fig. 4A). High magnification images showed that the majority of the HiLyte Fluor-555-labeled Aβ_42_ signal was found within astrocyte cell bodies (Fig. 4A, inset). Quantification showed that enhanced uptake of aggregated Aβ_42_ began at around 6 hrs post incubation and plateaued at around 12 hrs, while it took about 36 hrs for WT astrocytes to uptake about the same amount of HiLyte Fluor-555-labeled Aβ_42_ (Fig. 4B).

We also examined Aβ levels by Western blot. Although the levels of oligomeric Aβ_42_ in *Bace1*-null astrocytes were higher than that in WT controls during initial incubation (12 to 36 h), significantly less Aβ_42_ was found at the time of 72 hrs (Fig. 4C). This was further confirmed by multiple replication experiments (Fig. 4D), indicating that BACE1 deletion likely also enhanced degradation of oligomeric Aβ_42_ after initial uptake. In line with previous reports [20, 21, 29], astrocytes treated with Aβ_42_ appeared to enhance expression of BACE1 (Fig. 4C), suggesting that elevated BACE1 in AD brains might have vicious inhibitory effect on astrocytic Aβ clearance.

### Clusterin contributes to enhanced Aβ uptake in *Bace1*-null astrocytes

Because CLU is known to regulate Aβ clearance and degradation and elevation of the CLU protein level was evident, we chose to examine whether elevated CLU levels would contribute to enhanced Aβ uptake and degradation in *Bace1*-null astrocytes. To this end, we identified that one well-characterized siRNA (Santa Cruz Biotechnology) was able to reduce CLU protein levels in primary astrocytes in a dose dependent manner, with astrocytes treated with 80 pmol of *Clu* siRNA down-regulated about 48.6% expression of CLU (Supplemental Figure 3A and B).

**Fig. 5:**
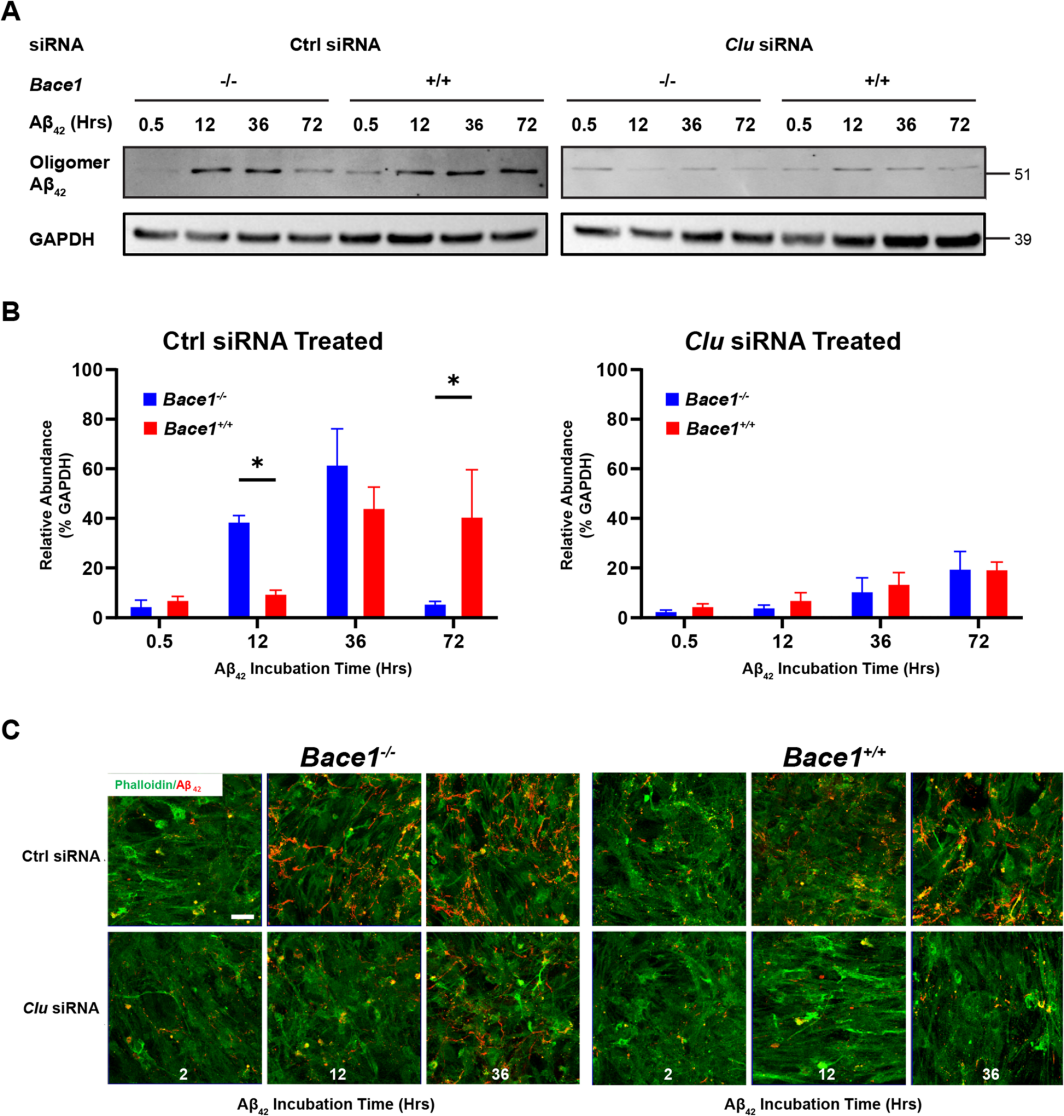
Clusterin underlies astrocyte endocytosis of Aβ in vitro. **A** Western blot of *Bace1*-null and WT-primary astrocytes pretreated with either 80 pmol of *Clu* siRNA or 80 pmol of control scrambled siRNA and incubated with 2 μM of Aβ_42_ for indicated times. Images indicate major bands for Aβ_42_ (human, oligomeric) and GAPDH. **B** Quantification of Western blot Aβ_42_ band intensity normalized to actin (*N* = 3, * *p*-value < 0.05, ** *p*-value < 0.01, *** *p*-value < 0.001; One-way ANOVA with Tukey post testing comparing between samples. **C** Confocal imaging of astrocyte primary cultures from BACE1-null and WT and pretreated with either Clu or control scrambled siRNA then treated with 2 μM of Aβ_42_ tagged with fluorescent Hilyte-555 for indicated incubation times. Stained with phalloidin (green) to mark F-actin and ToPro3 (blue) to mark nuclei. Scale bars represents 10 μm

We then pretreated *Bace1*-null and WT astrocytes with 80 pmol of *Clu* or control siRNA before treating astrocytes with aggregated Aβ_42_ and examined intracellular Aβ levels by either Western blot (Fig. 5A and B), or fluorescently tagged Aβ by confocal imaging (Fig. 5C). We showed that knocking down *Clu* in *Bace1*-null astrocytes reduced the uptake of Aβ_42_, when compared to *Bace1*-null astrocytes treated with control siRNA (Fig. 5).

Further quantification showed a clear shift in the uptake pattern (Fig. 5B): slowed uptake of Aβ_42_ by astrocytes when CLU levels were reduced, and treatment with Clu siRNA reduced total levels of Aβ_42_ in both *Bace1*-null and WT astrocytes from 0.5 to 36 hrs compared to control siRNA treated astrocyte (Fig. 5B). Confocal images also showed delaying uptake of Aβ_42_ when comparing *Bace1*-null astrocytes with and without specific *Clu* siRNA treatment (Fig. 5C).

Altogether, these results add evidence that CLU indeed plays a direct role in astrocytic clearance of Aβ. Furthermore, it suggests that increased CLU levels underlie the increased uptake of Aβ in *Bace1*-null astrocytes.

### BACE1 deficiency increases P38, ERK1/2 and cJun activity

**Fig. 6:**
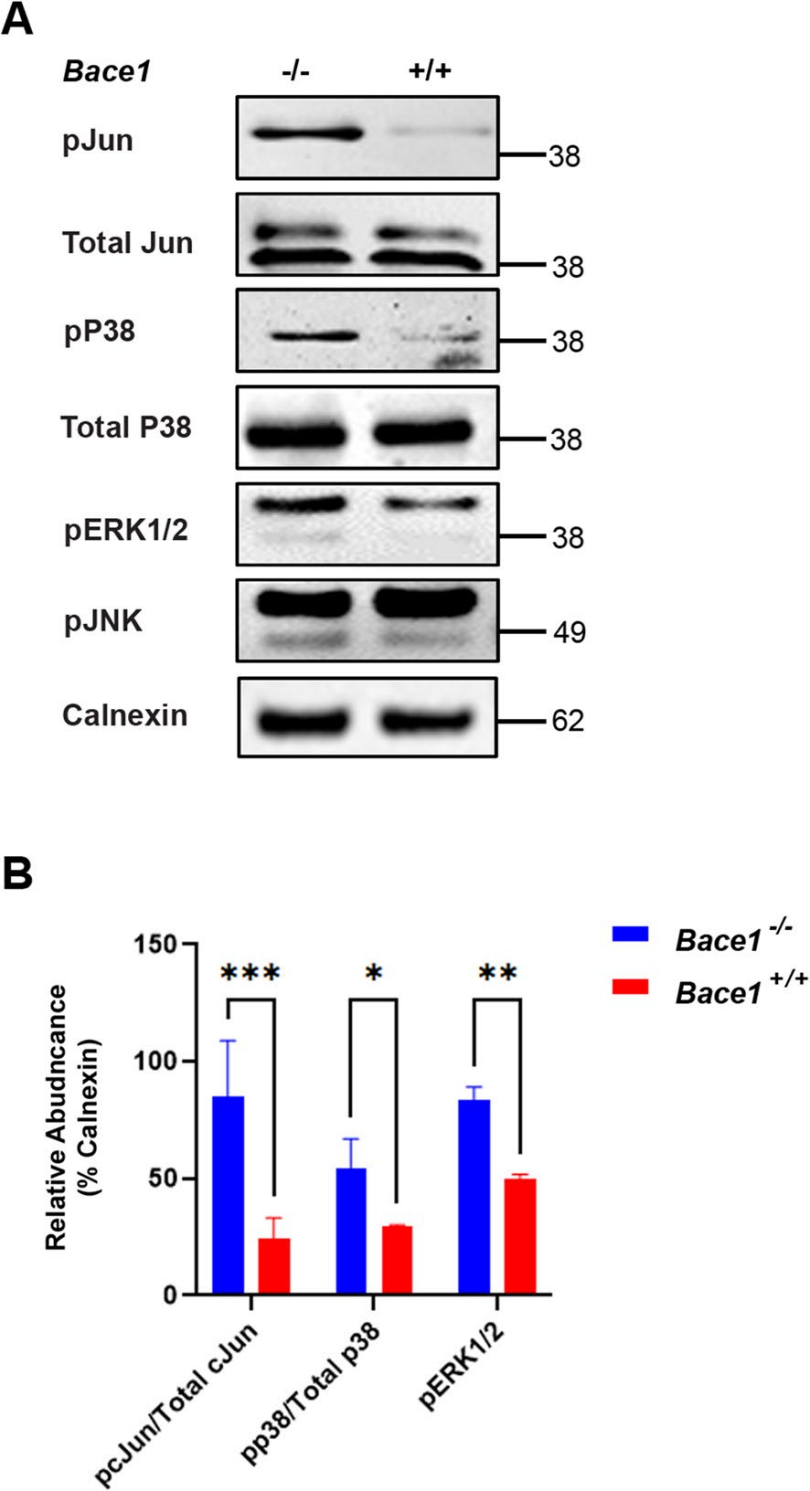
BACE1 deficiency enhances P38, ERK1/2, and cJun phosphorylation. **A** Western blot of BACE1-null and WT primary astrocytes lysates. Images indicate major bands for pJun, total Jun, pP38, total P38, pERK1/2, pJNK and Calnexin. **B** Quantification of pJun/total cJun, pP38/total p38, and pERK1/2 band intensity normalized to Calnexin (*N* = 3, * *p*-value < 0.05, One-way ANOVA with Tukey post testing comparing between samples)

In order to investigate signaling pathways responsible for the increased expression of *Clu* and other aforementioned DEGs in BACE1 deficient astrocytes, we performed a database search using BioMart to determine common transcription factors for these DEGs. Members of the activated protein 1 (AP-1) transcription family were the most commonly found elements to be related to DEGs of BACE1 deficient astrocytes, including *Cxcl14* and *Clu*. Furthermore, some members of the AP-1 family themselves were DEGs upregulated in BACE1 deficient reactive astrocytes (Fig. 2B, Table 1, and Supplemental Table 1). AP-1 activation is known to be modulated by upstream molecules such as P38, JNK1/2, and ERK1/2 [30, 31]. We found that the levels of JNK were not significantly changed, but levels of phosphorylated P38, Jun, and ERK1/2 were significantly elevated in *Bace1*-null astrocyte cultures (Fig. 6A); total Jun and P38 levels were not obviously changed. Quantification of replicated results confirmed this in Fig. 6B.

Our results imply that BACE1 deficiency in astrocytes results in an increase of pP38 and pERK1/2 activation, which then phosphorylates and activates downstream Jun and AP-1 mediated transcription of its downstream molecules such as *Clu*, which is related to enhanced astrocytic clearance of Aβ.

### BACE1 deficiency increases astrocytic insulin receptor signaling

We further explored how BACE1 deficiency would activate the aforementioned signaling molecules in astrocytes. It was previously reported that insulin receptor (IR) is a BACE1 cleavage substrate in the liver [32], and insulin signaling regulates both P38 and ERK1/2 MAPK activity. Furthermore, some DEGs of the insulin and insulin growth factor families were altered in *Bace1*-null astrocytes (Table 1 and Supplemental Table 1). IR is composed of a heterodimer dimer, α and β subunits, and the membrane anchored β subunit is identified as a BACE1 substrate. When *Bace1* is deleted, the IRβ subunit is no longer cleaved and more preserved IRβ subunit might be available for enhancing P38 and ERK1/2 activity. We therefore investigated whether BACE1 deficiency might also enhance astrocytic insulin receptor availability.

**Fig. 7:**
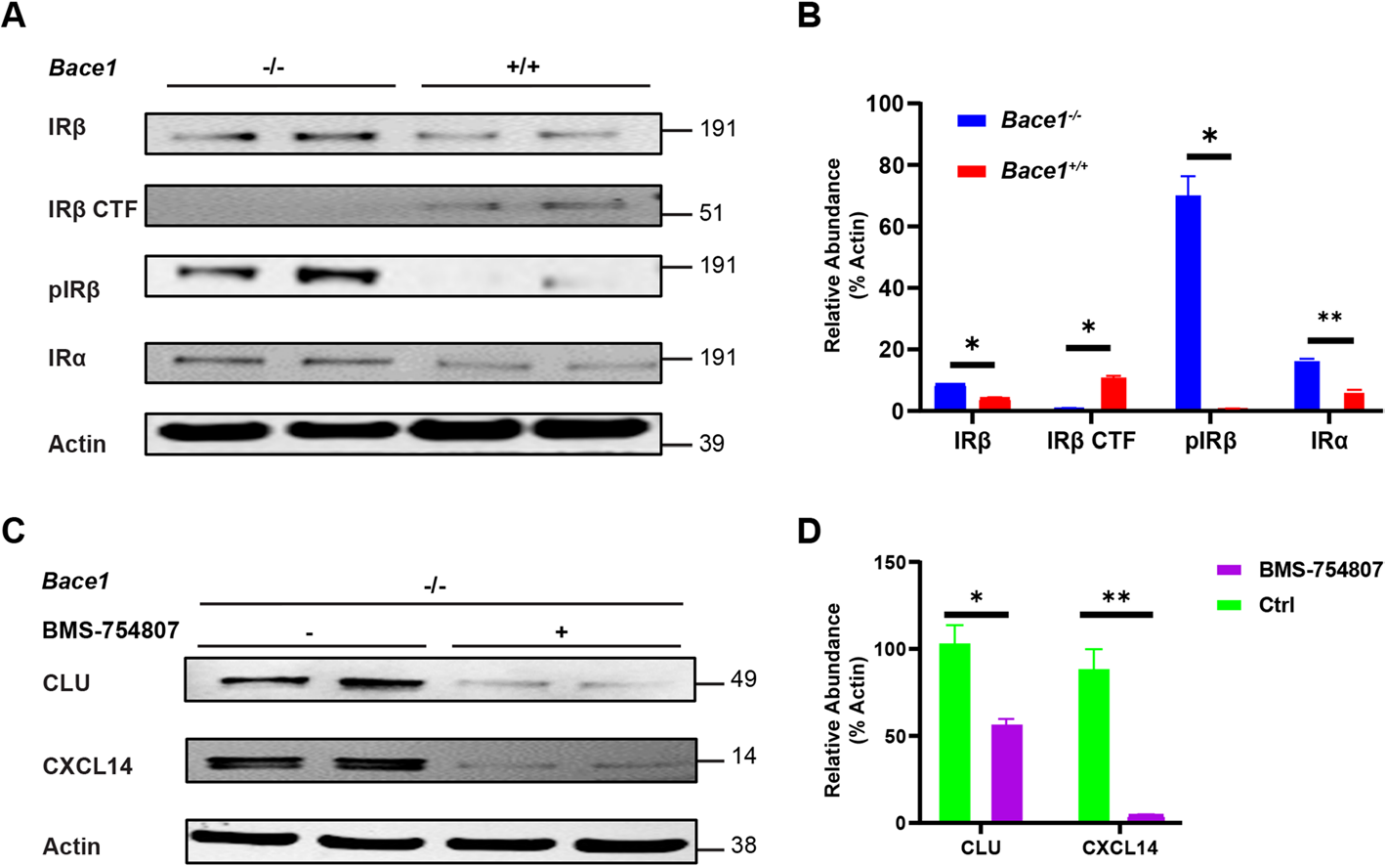
BACE1 deficiency preserves astrocytic IR bioavailability. **A** Western blot of *Bace1*-null and WT primary astrocytes culture lysates. Images indicate major bands for IRβ, IRβ CTF, pIRβ, IRα, and actin. **B** Quantification of IRβ, IRβ CTF, pIRβ, and IRα band intensity normalized to actin (*N* = 4, * *p*-value < 0.05, ** *p*-value < 0.01, One-way ANOVA with Tukey post testing comparing between samples. **C** Western blot of BACE1-null primary astrocytes culture lysates treated with 1 μM of BMS-754807 or control. Images indicate major bands for CLU, CXCL14, and actin. **D** Quantification of IRβ, IRβ CTF, pIRβ, and IRα band intensity normalized to actin (*N* = 4, * *p*-value < 0.05, One-way ANOVA with Tukey post testing comparing between samples)

Western blot analysis revealed that BACE1 deficiency significantly reduced levels of BACE1-cleaved IRβ C-terminal fragment (CTF) in *Bace1*-null astrocytes compared to their WT controls (Fig. 7A), consistent with prior results that BACE1 deficiency abrogated cleavage of IRβ in astrocytes. An increase of total mature IRα and IRβ levels was also noted, while as well as a much larger increase in the phosphorylated IRβ (pIRβ) subunit (Fig. 7A), which is required for downstream activation for pP38 and ERK1/2 MAPK. Quantification of replicated results confirmed changes of these proteins (Fig. 7B). Altogether, this suggests that astrocytic BACE1 regulated IR receptor availability by cleavage in a manner similar to liver BACE1 and IR.

We further tested whether insulin signaling might be responsible for the elevated CLU and CXCL14 levels in *Bace1*-null astrocytes. Since insulin is already present in the G-5 supplemented astrocyte media, we inhibited insulin signaling by using BMS-754807 (SelleckChem), a potent IR tyrosine kinase phosphorylation inhibitor (Fig. 7C and D). BMS-754807 treatment resulted in a potent reduction of both CLU and CXCL14 levels in *Bace1*-null astrocytes (Fig. 7C). Quantification of replicated results confirmed changes of these proteins (Fig. 7D). This suggests that elevated CLU and CXCL14 levels in *Bace1*-null astrocytes is dependent on IR signaling.

Altogether, this suggests that astrocytic BACE1 regulated IR availability by cleavage in a matter similar to liver BACE1 and IR and that IR signaling may play a role in reactive astrocyte amyloid clearance.

### Targeted deletion of *Bace1* in astrocytes increases astrocytic levels of IR, CLU and CXCL14

**Fig. 8:**
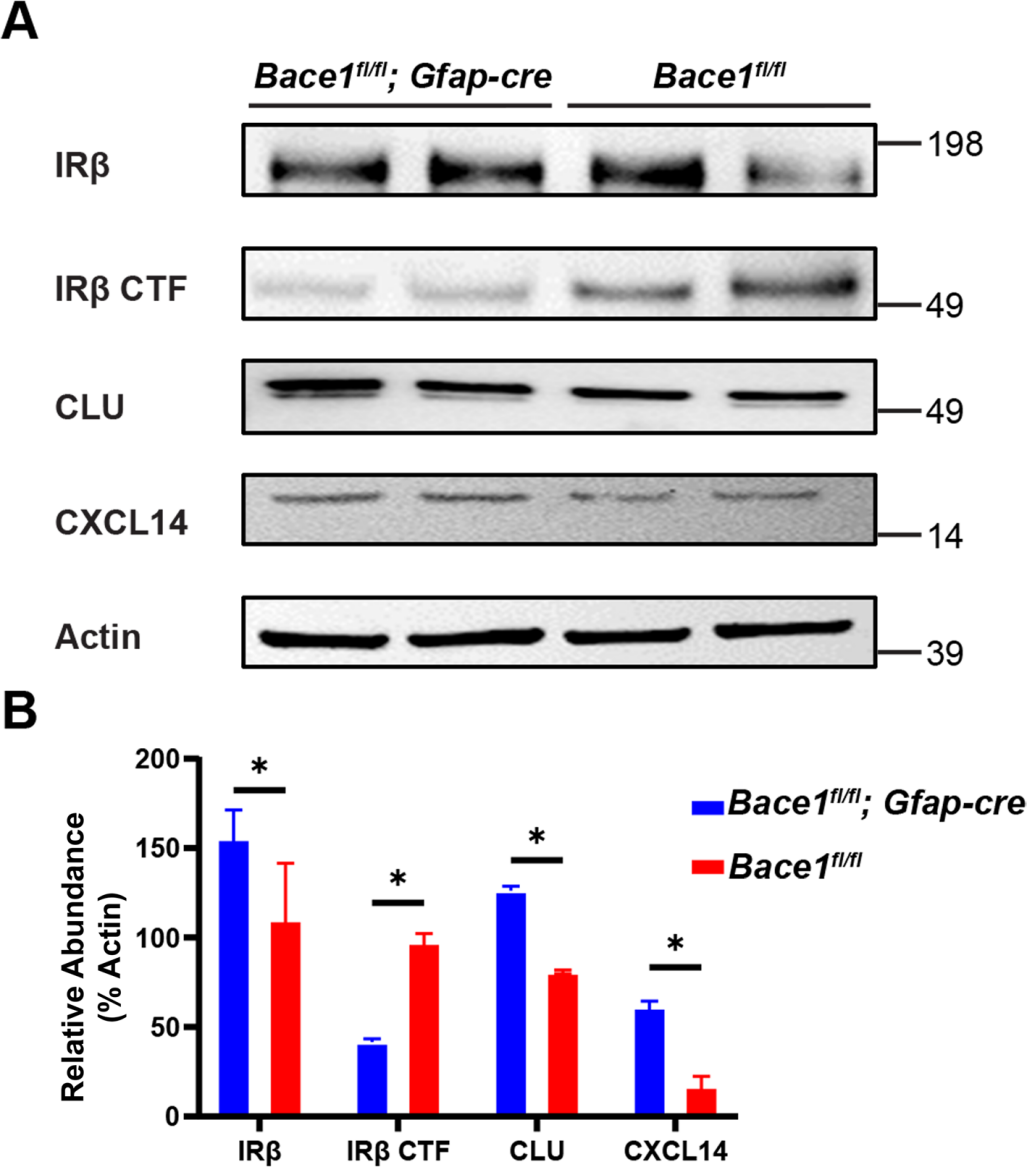
Targeted astrocytic deletion of *Bace1* enhances IR bioavailability and downstream amyloid clearance proteins. **A** Western blot of ACSA2 + astrocyte enriched lysates from *Bace1*^*fl/fl*^*;Gfap-cre* and *Bace1*^*fl/fl*^ mice. Images indicate major bands for IRβ, IRβ CTF, CLU, CXCL14, and actin. **B** Quantification of indicated protein intensity normalized to actin (*N* = 3, * *p*-value < 0.05, One-way ANOVA with Tukey post testing comparing between samples)

To investigate whether in vivo astrocytic BACE1 deficiency results in increased IR, CLU and CXCL14, we bred *Bace1*^*fl/fl*^ mice with *Gfap-cre* mice [Tg-Gfap-cre 73.12Mvs, the Jackson lab] and examined *Bace1*^*fl/fl*^*;Gfap-cre* mice, which conditionally deleted  *Bace1* mostly in astrocytes due to expression of Cre recombinase by the mouse Gfap promoter [33]. To avoid potential effects from other Gfap-expressing lineage cells, we isolated astrocytes from 2-month old *Bace1*^*fl/fl*^*;Gfap-cre* mice using ACSA2^+^ immunobeads and the purified ACSA2 + astrocytes were used for Western blot (Fig. 8).

We found that astrocytes from *Bace1*^*fl/fl*^*;Gfap-cre* mice had an increased amount of mature IRβ and reduced IRβ CTF compared to *Bace1*^*fl/fl*^ astrocytes in a manner similar to above in vitro conditions (comparing Fig. 7 and 8). We also found a significant increase in CLU and CXCL14 protein levels (Fig. 8). Altogether, this suggests that BACE1 deficiency increases astrocytic IR signaling and its downstream molecule CLU and CXCL14 in a mostly cell autonomous manner both in vivo and in vitro.

### *Bace1* deletion in astrocytes decreases amyloid plaque levels

**Fig. 9:**
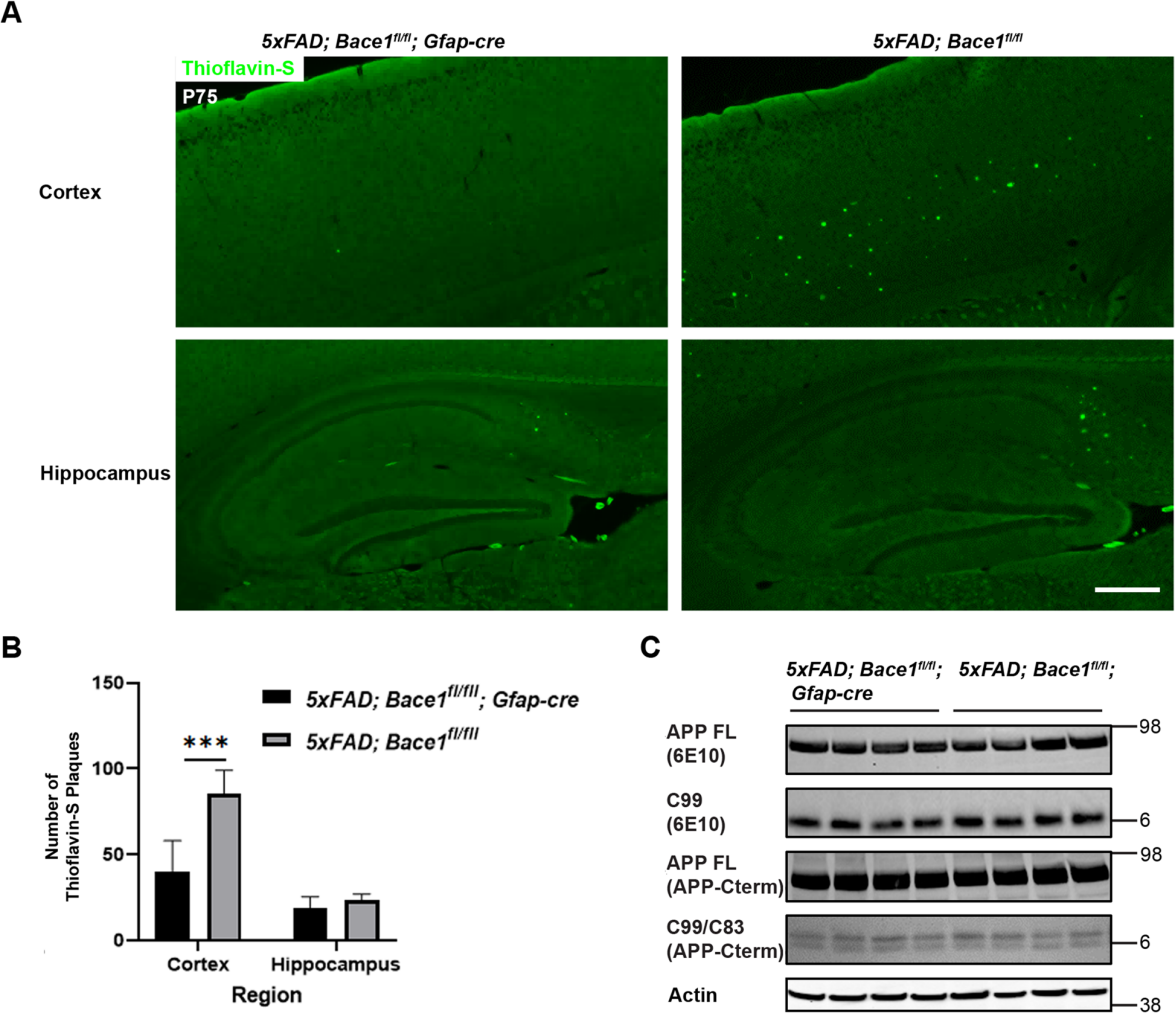
Targeted astrocytic deletion of *Bace1* reduces amyloid plaques by increasing Aβ clearance. **A** Thioflavin-S staining of amyloid plaques from saggital brain sections of *5xFAD;Bace1*^*fl/fl*^*;Gfap-cre* and *5xFAD;Bace1*^*fl/fl*^. Scale bar indicates 100 µm (**B**) Quantification of Thioflavin-S positive amyloid plaques calculated by counting serial sagittal sections, which were selected at 10-section intervals (*N* = 7 for *5xFAD;Bace1*^*fl/fl*^*;Gfap-cre* and *5xFAD;Bace1*^*fl/fl*^). **C** Western blot of cortical lysates from *5xFAD;Bace1*^*fl/fl*^*;Gfap-cre* and *5xFAD;Bace1*^*fl/fl*^ mice. Images indicate APP full length, APP C-terminal C99 (detected by 6E10) and C99/C83 bands (detected by APP A8717 antibody). Actin was included as loading control

To further understand the effect of astrocytic *Bace1* deletion on amyloid pathology, we bred *Bace1*^*fl/fl*^*;Gfap-cre* with *5xFAD;Bace1*^*fl/fl*^* mice* to generate *5xFAD;Bace1*^*fl/fl*^*;Gfap-cre* and *5xFAD;Bace1*^*fl/fl*^ mice for comparison. We used P75 female mice from each genotype, as female 5xFAD mice show less variations in amyloid pathology [34]. Thioflavin-S staining of fixed brain sections revealed an overall less amyloid depositions in *5xFAD;Bace1*^*fl/fl*^*;Gfap-cre* mouse brains compared to *5xFAD;Bace1*^*fl/fl*^ mouse controls (Supplemental Figure 4). In an enlarged view, it was clear that less Aβ plaques were formed in the *5xFAD;Bace1*^*fl/fl*^*;Gfap-cre* cortex compared to controls (Fig. 9A), and was confirmed by quantification (Fig. 9B). Although, levels of Aβ plaques appeared to be reduced in the subiculum and hippocampus of *5xFAD;Bace1*^*fl/fl*^*;Gfap-cre* mice, further quantification showed that this decrease in hippocampus was, overall, not significant (Fig. 9B).

We also examined the effect of astrocytic BACE1 on total amyloid processing and generation by Western blot (Fig. 9C). We found that total levels of full length APP, C99, and C83 cleavage substrates appeared to be unchanged across cortical samples. This most likely indicates that brain Aβ levels are mainly resulted from neuronal sources while the contribution of astrocytic Aβ levels are minimal. It is noted that neurons have higher levels of BACE1 and APP than glial cells. Most likely, astrocytic BACE1 deletion induced IR signaling and increased CLU levels, which contribute to the enhanced glial Aβ clearance.

## Discussion

BACE1 is a type I transmembrane aspartyl protease, which mainly cleaves membrane bound proteins such as amyloid precursor protein (APP), type I and type III neuregulin and Sez6L [35, 36]. Since its discovery, BACE1 studies have mostly focused on the role of BACE1 in neurons including amyloid production, myelination, and synaptic function [5, 10–12, 14, 16, 36]. Furthermore, BACE1 inhibitors used in clinical trials have largely failed due to lack of efficacy and because of neuronal side effect from global BACE1 inhibition [5, 10, 37, 38]. In contrast, relatively few studies reveal the unique role of BACE1 in astrocytes, despite known expression of BACE1 in reactive astrocytes and its contribution to astrocyte development [20, 22]. We provided the first evidence that astrocytic BACE1 regulates astrocytic Aβ clearance by promoting more beneficial astrocytes in the reactive state, and inhibition of astrocytic BACE1 might be a novel target for future AD therapies, which can contribute to the reduction of Aβ deposition.

In this study, we conducted unbiased single cell RNA-seq experiments and compared transcriptomic profiles in mouse brain astrocytes, with or without BACE1. We provided evidence that BACE1 deficiency increases a population of reactive astrocytes expressing higher levels of genes such as *Clu*, which are known to enhance clearance of aggregated Aβ. Cultured astrocytes with BACE1 deficiency showed an increase in Aβ clearance and degradation, suggesting that targeted inhibition of BACE1 or specific downstream pathways in astrocytes may likely be beneficial for AD treatment. Furthermore, we found evidence that astrocytic IR, a potential BACE1 cleavage substrate, and downstream pathways might contribute to astrocytic expression of astrocytic clearance genes both in vitro and in an astrocyte specific BACE1 deficiency mouse model.

In AD development of brains, astrocytes are activated and reactive astrocytes are often found surrounding amyloid plaques. Reactive astrocytes contribute to amyloid clearance, directly by endocytosis [39] or producing amyloid degrading enzymes [40, 41], and indirectly by preventing amyloid aggregation [42, 43] or enhancing clearance by other cell types [44, 45]. However, as AD pathology progresses, reactive astrocytes not only experience a loss of amyloid clearance efficiency, but may also play a role in promoting neuroinflammation and toxicity [46]. Furthermore, genetic descriptions of various disease-related astrocyte subtypes [25, 47, 48] have made it clear that an unbiased, single cell genetic approach is key to understanding how astrocyte subtypes contribute to neurological diseases. To this end, we used single cell RNA-seq to compare various astrocyte clusters from BACE1 deficient mice. In comparing *Bace1*-null and wild type mice, we found an increase in reactive astrocyte clusters, which is in line with the previous observation showing increased astrocyte genesis and reactivity in the *Bace1*-null mice [22]. When comparing the transcriptomes of individual UMAP defined astrocyte clusters between *Bace1*-null and wild type mice, a variety of known AD-related DEGs were noted (Fig. 2B and Supplemental Table 1). Among these upregulated DEGs from *Bace1*-null reactive astrocytes, *Clu* gained our particular attention, because it is highly expressed in astrocytes, is a top GWAS risk factors for the development of AD, and has a known role in clearance of aggregated Aβ [49–52]. *Cxcl14* was another highly expressed DEGs on the list, although total number of *Cxcl14* expressing cells was relatively low. CXCL14 is chemokine with known roles in peripheral inflammation, but is less explored in the CNS or in the field of neurodegeneration. However, GWAS indicates that two SNPs near the *Cxcl14* locus is associated with increased tau and p-tau [53]. We validated that both of these two genes were elevated in cultured *Bace1*-null primary astrocytes compared to WT controls (Fig. 2D). Intriguingly, CLU and CXCL14 levels appeared to be increased with Aβ_42_ treatment (Fig. 2D). In 14-month old *5xFAD;Bace1*^*fl/fl*^*; UBC-creER* mice, elevation of these proteins were also prominent compared to *5xFAD;Bace1*^*fl/fl*^ (Fig. 3A).

To understand the effect of BACE1 deficiency on amyloid clearance, we conducted in vitro experiments to compare astrocytes cultured from WT and *Bace1*-null mice to avoid the inference from other cell types. An increase of fluorescence-labeled Aβ_42_ uptake by *Bace1*-null astrocytes for the first 12 hrs was clearly more obvious than control astrocytes. With the consistently detected elevation of CLU in *Bace1*-null astrocytes, we asked whether CLU levels would affect uptake of the fluorescence-labeled Aβ42 in *Bace1*-null astrocytes. *Clu* knockdown resulted in decreased uptake by both *Bace1*-null and WT astrocytes, suggesting that higher level of CLU correlates with more uptake of Aβ_42_. Interestingly, *Clu* siRNA treatment in *Bace1*-null astrocytes still had slightly more uptake of Aβ_42_ than Clu siRNA-treated WT astrocytes, and this observation indicates that other BACE1- regulated genes in astrocytes may also contribute to enhanced uptake of Aβ_42_.

Our validated finding is in line with the published observation regarding the beneficial effect of CLU on astrocytic uptake and degradation of Aβ_42_ [26, 27]. CLU is also found to enhance brain endothelial vascular clearance of Aβ_42_ by protecting against AD associated metabolic stress, glutamate toxicity, and synaptic dysfunction [54, 55]. We further explored how BACE1 deficiency alters CLU levels, and our mechanistic study implied that CLU elevation is likely related to astrocytic insulin receptor (IR) signaling. IR has α and β subunits and its β subunit (IRβ) was previously identified as a BACE1 substrate in the liver [32]. We found that BACE1-cleaved C-terminal fragment (CTF) of IRβ was reduced on the western blot by BACE1 deficiency. In *Bace1*^*−/−*^ astrocytes, full length IRβ is consistently higher both in cultured astrocytes and with astrocyte specific knockout for BACE1, detectable even without γ-secretase inhibition. This elevation of full length IRβ and reduced IRβ CTF is attributable to abrogated cleavage by BACE1 (Fig. 7 and 8). The availability of more functional IRs on the surface of astrocytes of BACE1 deficient astrocytes would most likely explain the elevation of phosphorylated IRβ, and its downstream signaling molecules P38, ERK1/2, and Jun (Fig. 6). We also discovered upregulation of several members of the AP-1 transcription factors in BACE1-null astrocytes (Supplemental Table 1 and Fig. 2B), similar to changes seen in deletion of *Bace1* in microglia [56]. Although total protein levels of several AP-1 transcription factors were not increased in BACE1-null astrocytes, interestingly, we found increased phosphorylation of Jun (Fig. 6), indicating an upregulation of its transcription activity. Consequently, these transmitted signals will enhance AP-1 mediated transcription of *Clu*. The enhancement of insulin signaling has been pursued in AD clinical trials for purported benefits in adult neurogenesis and synaptic function as well.

To understand whether the effects of BACE1 inhibition on astrocytic pathways and Aβ clearance was astrocyte specific or due to cell–cell communication in vivo, we generated an astrocytic BACE1 knockout model (*Bace1*^*fl/fl*^*;Gfap-cre*). In astrocytes acutely extracted from *Bace1*^*fl/fl*^*;Gfap-cre* mice, we saw similar increases in mature IR, reduced IR CTF, increased CLU, and increased CXCL14 in a manner similar to previous in vitro results (Fig. 8). Importantly, we also found that astrocytic BACE1 deficiency reduces cortical Aβ load in a 5xFAD mouse background (*5xFAD;Bace1*^*fl/fl*^*;Gfap-cre*), without altering total Aβ generation in vivo (Fig. 9), but rather via *Cxcl14*^high^ and *Clu*^high^ astrocytes. In a pilot observation from small numbers of mice, amyloid plaques in 10-month old of *5xFAD;Bace1*^*fl/*^*fl;Gfap-cre* were not obviously reduced when compared to *5xFAD;Bace1*^*fl/fl*^ controls, likely due to more predominant effects of available BACE1 in neurons and microglia. We have recently shown that deletion of *Bace1* in microglia appears to have a strong effect on removing amyloid deposition [57], and Aβ is continuingly produced from neurons.

## Conclusion

**Fig. 10:**
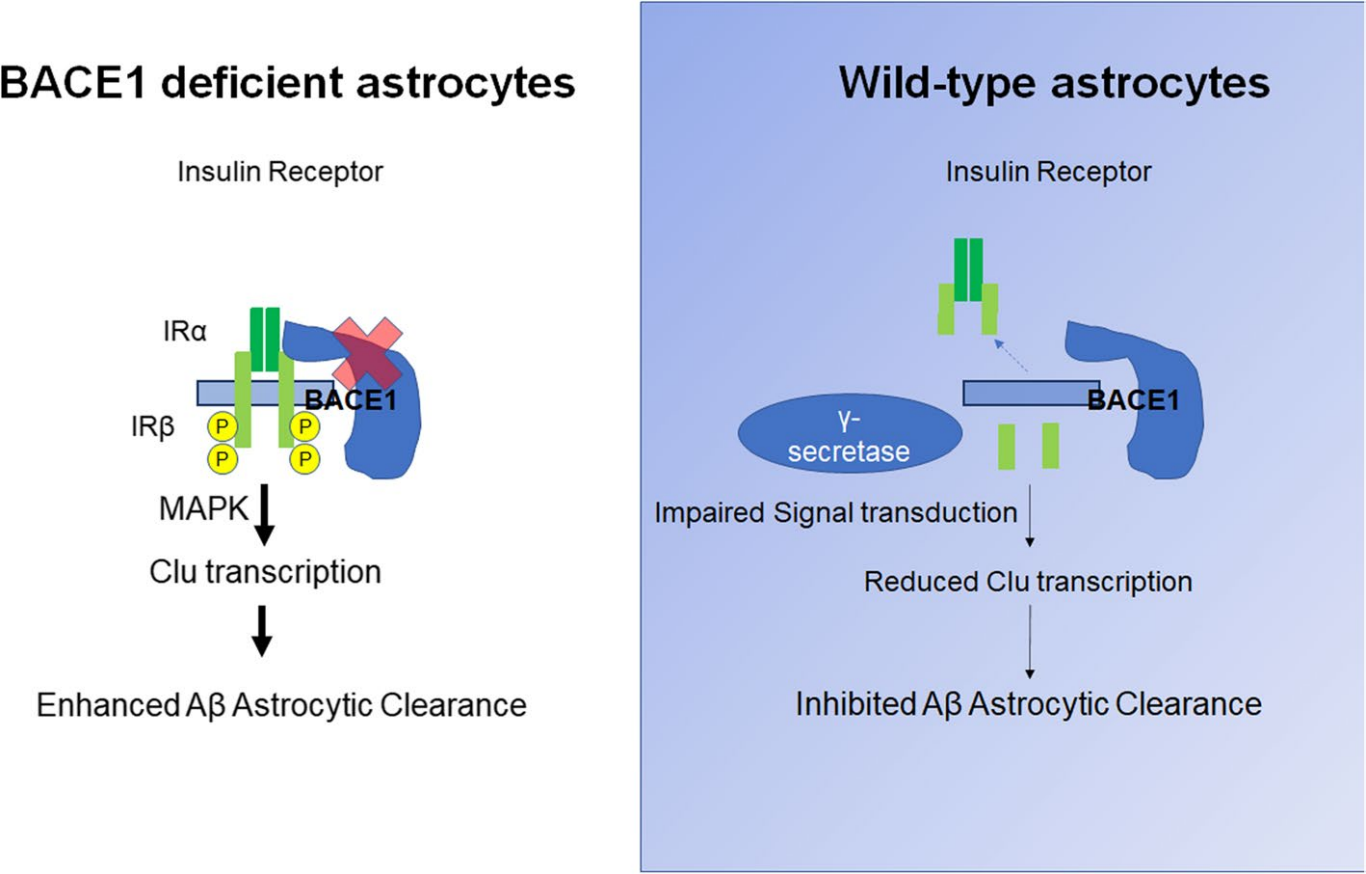
Schematic diagram illustrates BACE1 deficiency in astrocytes and its effect on the astrocytic function. BACE1 deficiency decreases cleavage of astrocytic inulin receptor (IR). This abrogated cleavage, compared to wild type astrocytes, in turn leads to gain of function of insulin receptor signaling to its downstream molecules such as P38 and ERK1/2. This increased MAPK pathway activates AP-1 transcription to elevate *Clu* levels, and to enhance amyloid endocytosis by BACE1-null reactive astrocytes

We describe a model of BACE1 inhibition that enhances astrocytic clearance of Aβ, most likely in a cell-specific and autonomous manner, by increasing insulin signaling, which in turn upregulates expression of Aβ clearance genes such as *Clu* (see graphic illustration in Fig. 10). Increasing BACE1 levels in AD reactive astrocytes is likely to cause a vicious cycle that may block an efficient astrocytic clearance of Aβ. Hence, specific inhibition of BACE1 in astrocytes may be an alternative strategy for reducing Aβ in human AD therapy that is worthy of further exploration in future.

## Materials and methods

### Mouse strains and breeding strategy

Astrocyte specific *Bace-1* deletion was achieved by breeding Tg-Gfap-cre, Gfap specific Cre expressing mice (73.12Mvs, JAX stock#012,886, the Jackson Laboratory) with Bace-1 conditional mice (*Bace-1*^* fl/fl*^), carrying loxP-flanked genes as previously described [15]. The resulting *Bace1*^*fl/*+^*;Gfap-cre mice* were crossed with *Bace1*^*fl/fl*^ mice to obtain a colony with the following genotype: *Bace1*^*fl/fl*^*;Gfap-cre*. *Bace1*^*fl/fl*^*;Gfap-cre* mice were maintained by breeding with *Bace-1*^* fl/fl*^. Female *Bace1*^*fl/fl*^*;Gfap-cre* mice were also crossed with male *5xFAD;Bace1 *^*fl/fl*^ resulting in *5xFAD;Bace1*^*fl/fl*^*;Gfap-cre* mice. Various age groups of *5xFAD;Bace1*^*fl/fl*^*;Gfap-cre* and *5xFAD;Bace1*^*fl/fl*^ females were used for downstream Western blot and amyloid staining due to reported inconsistencies in male 5xFAD amyloid pathology [34].

Adult conditional knockout *Bace1*^*fl/fl*^*;UBC-CreER* and *5xFAD;Bace1*^*fl/fl*^*;UBC-CreER* were bred and maintained as previously described [15]. All lines were routinely backcrossed with C57BL/6 J mice for at least five generations to ensure consistent genetic background for phenotypic analyses. BACE1-null mice were obtained by heterozygous breeding of *Bace1*^*+/-*^ males and females (Jax stock# 004,714, Jackson Lab) as previously described.

All experimental protocols were approved by the Institutional Animal Care and Use Committee of the Lerner Research Institute and University of Connecticut Health Center in compliance with the guidelines established by the Public Health Service Guide for the Care and Use of Laboratory Animals.

### Adult mouse astrocyte isolation

Astrocytes were isolated from mouse brains using adult brain dissociation kit (MiltenyiBiotec, cat.no. 130–107-677) for single-RNA sequencing (see below), as well as isolating astrocytes from *Bace1*^*fl/fl*^*;Gfap-cre* and *Bace1*^*fl/fl*^. Briefly, mice were perfused with 25 mL of PBS and forebrains were isolated and transferred to gentleMACS Dissosciator (Milltenyi) to form a single-cell suspension. Subsequently, myelin, cell debris, and erythrocytes were removed and remaining cells were immunolabelled with ACSA2 immunomagnetic beads (cat. No. 130–097-678). The cell suspension was passed though magnetic columns and QuadroMACS separators, which retained ACSA2-positive cells on the column. This ACSA2 immunomagnetic bead MACS method is reported to be less disruptive to glial cell processes and reactivity than microfluidic systems such as FACS [58].

### Single-cell RNA sequencing

For single cell RNA-sequencing, ACSA2 positive astrocytes from littermate controlled 2-month-old *Bace1*-null and WT mice. 3 separate samples per group (2 males, 1 female) were submitted for downstream analysis. Astrocytes from 3 pooled brains (2 males, 1 female) from 14-month old *5xFAD;Bace1*^*fl/fl;UBC−creER*^ and *5xFAD;Bace1*^*fl/fl*^ mice were also submitted for scRNA seq. Astrocytes were checked for cell number and viability. Samples with little debris and > 80% viability were then used for single cell RNA sequencing (University of Connecticut Single Cell Genomics). About 12,000 cells of each sample type were loaded onto 10X Chromium Single Cell RNA sequencing chips. Sample mRNAs were barcoded and subsequently converted into cDNA. After cDNA library quality inspection, libraries were sequenced using an Illumina Novaseq 6000 sequencer. Custom CellRanger pipeline (University of Connecticut Single Cell Genomics) provided initial UMI counts and tSNE clustering. Raw scRNA-seq data will be provided to the NIH Gene expression Omnibus (GEO) for open-source use.

The data analysis was taken into consideration of a published guidance [24]. For secondary analysis, the Seurat version 4 packaged in R (Satija lab) was used to determine differential gene expression using normalized read counts for identifiable genes, reclustering, displaying violin plots of gene expression, and mapping gene expression in cells. For violin and dimension plots of gene expression, scaling excludes outlier cells below 5^th^ or above 95^th^ percentile of range.

Quality control parameters were set to only include cells with greater than 1,000 identifiable genes, less than 25,000 read counts, and less than 20% mitochondrial RNA. Filtered samples were then grouped by genotype and read counts were normalized and transformed using SCTransform (Supplementary Figures 1 and 2) PCA dimensional reduction was followed by UMAP clustering. Identified reactive astrocyte cluster transcriptomes were then compared with a log normalized scaling factor of 100,000 in order to generate a number of differentially expressed genes, with low overall expression. DEG adjusted p-values are presented as p-values for analysis.

### Primary perinatal astrocyte culture and isolation

Primary astrocytes cultures were prepared from littermate controlled perinatal mouse pups resulting from *Bace1*^*+/-*^ X *Bace1*^*+/-*^ breeding. Pups were genotyped at P0 (see below) and then primary mixed glia were prepared from P1-3 as described previously [59, 60]. Briefly, brain tissue was isolated from skulls, bisected, and then incubated with 0.25% trypsin at 37 °C for 15 min, with occasional gentle swirling. Afterwards, tissue solution was gently triturated with fire-polished glass pipettes and passed through a 70 micron filter to form a single-cell suspension. The mixed glia culture was cultured in a T75 flask in DMEM-F12 containing 10% heat-inactivated fetal bovine serum, 2 mM L-glutamine, and 1% penicillin/streptomycin (Life Technologies) for 12–14 days. Mixed glia cultures were split twice with 4–5 day intervals to achieve a pure mature astrocyte culture.

Astrocytes were seeded 1 × 10^6^ onto 6 well plates for Western blot purpose, and 1 × 10^4^ for 8 well chamber sides for confocal staining. Media was switched to DMEM-F12 medium containing 1% serum-free G5 astrocyte media supplement (Gibco) for 12–24 hrs before treatment. Cells were maintained and grown in a humidified atmosphere of 5% CO_2_ at 37 °C.

### Preparation of aggregated Aβ_42_

Non-tagged Aβ_42_ peptide (ThermoFisher) and fluorescent Hilyte 555 tagged- Aβ_42_ (Anaspec) were prepared according to previous reports [28]. Briefly, peptides were solubilized in 0.1% NH_4_OH containing 0.01% (w/v) NaN_3_, and further re-suspended in DMEMF12 (pH 7.4) and stored at -20 °C. Prior to use, peptides were oligomerized by incubation with constant rotation for 24 hrs at 4 °C. Formation of beta-sheets was detected by Thioflavin-T fluorescence indicating the formation of aggregated Aβ_42_ (data not shown). Cells were treated with 2 μM of aggregated Aβ_42_ for 36 hrs for most experiments, except for Western blotting of phosphorylated signaling pathways, in which cells were treated with Aβ_42_ for 4 hrs or otherwise indicated.

### Clusterin siRNA knockdown

Knockdown of *Clu* in astrocyte cultures was attained by using Clusterin siRNA reagents (Santa Cruz Biotechnology, catalog no. sc-43689) or control scrambled siRNA (Santa Cruz Biotechnology, catalog no. sc-37007) according to manufacturer’s instructions. Briefly, siRNA plasmids were combined with siRNA transfection medium and duplexed with siRNA transfection media. Astrocyte specific G-5 media was removed and replaced with siRNA duplexed transfection solution for 6 hrs. Afterwards transfection solution was replaced with astrocyte specific G-5 media.

For determining correct dosage, cells were incubated with 10, 20, 40 or 80 pmol of *Clu* siRNA or 80 pmol of Ctrl siRNA, before switching to astrocyte media. After 72 hrs in astrocyte media, cells were lysed and probed for CLU expression. After optimization, cells were treated with 80 pmol of *Clu* or Ctrl siRNA before treatment with aggregated Aβ_42_ either untagged (Western blot) or tagged with HiLyte 555 (Cytochemistry imaging).

### Western blotting

Astrocytes from perinatal culture or acutely isolated from adult mice were washed with ice-cold PBS and then were lysed in RIPA lysis buffer containing 50 mM Tris–HCl (pH 7.4), 1 mM EDTA, 100 mM NaCl, 0.1% SDS, 1 mM PMSF, 1 mM sodium orthovanadate, 1 μg/ml leupeptin, 1 μg/ml pepstatin, and 10 μg/ml aprotinin, for 10–30 min at 4 °C with rotation. The lysate was collected and further bath sonicated on ice for 30 sec on and off cycles at 60 Hz for 5 min and then centrifuged at 15,000 × g for 30 min at 4 °C. Total brain lysates were sonicated by drill sonicator in RIPA buffer before centrifugation. Protein concentrations were determined using a BCA assay kit (Pierce). Equal amounts of protein from each sample were loaded and electrophoretically resolved on 4%–12% SDS-PAGE (NuPAGE system, Life Technologies) gels with MOPS or MES running buffer (Thermofisher). After electrophoresis, proteins were transferred to nitrocellulose membranes at 100 V for 2 hrs. The membranes were washed with PBS with 1:2,000 20% Tween-20 and blocked with 1% bovine serum albumin (BSA) and 0.5% evaporated milk for 1 hr at room temperature. The membranes were probed with primary antibody (see table for antibody list), followed by incubation with appropriate secondary HRP-conjugated antibody (1:2,000). The antibody-bound proteins were detected by an iBright 1500 imaging system (Invitrogen). To ensure equal loading, the blots were reprobed with monoclonal anti-actin (1:10,000) or calnexin (1:1,000). For quantification purposes, band intensities of immunoblots were analyzed using imageJ software. For phosphorylated antibodies, membranes were washed and blocked in Tris-Buffered Saline with 1:2,000 of 20% Tween-20 and blocked in 5% BSA.

### Cytochemistry imaging

After treatments with aggregrated HiLyte Fluor-555-labeled Aβ_42_ (Anaspec, Fremont, CA), astrocytes were washed with 3 times with PBS for 5 min, before fixation with 4% formaldehyde for 10 min at room temperature, and permeabilization with 0.1% TritonX-100 for 5 min. After careful washing, cells were incubated with 100 nM of Alexaflour 488 phalloidin (Thermofisher A12379) and Topro3 (Thermofisher T3605) diluted in 1% BSA/PBS for 10 min at room temperature. Cells were washed then mounted on coverslips with 90% glycerol, 20 mM Tris–HCL (1 M, pH 8.8), and 0.5% (w/v) p-Phenylenediamine (CSHB Protocol). Images were captured with either a Leica TCS-SP8-AOBS or Zeiss LSM 800 Confocal microscope.

Images were quantified with ImageJ software. Integrated density of HiLyte Fluor-555-labeled Aβ_42_ fluorescence intensity with the bounds of a mask based upon phalloidin defined maximum threshold was measured and normalized to phalloidin mask area and nuclei marked by ToPro3.

### Quantification of amyloid plaque load

Quantification of amyloid plaques was conducted with serial *sagittal* sections, which were selected at 10-section intervals. Amyloid plaques were labeled with 0.05% Thioflavin-S. Images were captured by BZ-X800 microscope (Keyence) using a 2 × objective and stitched together with the Keyence stitching module. Total plaque numbers in the cerebral cortex and hippocampus (including subiculum) were counted using ImageJ software (National Institutes of Health). Seven female *5xFAD;Bace1*^*fl/fl*^ ;Gfap-cre and six female *5xFAD;Bace1*^*fl/fl*^ mice in each age group were used.

### Genotyping primers

**Table:** 

**Primer Target**	**Sequence (5’- > 3’)**
APPSwFlLon – common	ACC CCC ATG TCA GAG TTC
APPSwFlLon – mutant reverse	CGG GCC TCT TCG CTA TTA C
APPSwFlLon – wild type reverse	TAT ACA ACC TTG GGG GAT GG
Bace1 – forward	AGG CAG CTT TGT GGA GAT GGT G
Bace1 mutant	TGG ATG TGG AAT GTG TGC GAG
Bace1—reverse	CGG GAA ATG GAA AGG CTA CTC C
Gfap-cre forward	TCC ATA AAG GCC CTG ACA TC
Gfap-cre reverse	TGC GAA CCT CAT CAC TCG T
Loxp forward	TCTGACGATGGCACACATAAGC
Loxp reverse	TGCTAGTGTTTCCTGTCACCTG
UBC-creERt2 forward	GAC GTC ACC CGT TCT GTT G
UBC-creERt2 reverse	AGG CAA ATT TTG GTG TAC GG

### Antibody list used for the study

**Table:** 

**Antibody Name**	**Catalog no**	**RRID:**	**Manufacturer**
Actin (Clone AC-15)	A5441	AB_476744	Sigma
APP C-terminal	A8717	AB_258409	Sigma
6E10	803,002	AB_662804	Biolgend
Amyloid Beta (Oligomer), A11	AHB0052	AB_2536236	ThermoFisher Scientific
BACE1 (D10E5)	5606	AB_1903900	Cell Signaling
Caln	C4731	AB_476845	Cell Signaling
GFAP	3670	AB_2130165	Cell Signaling
Clusterin-a (B-5)	sc-5289	AB_673566	Santa Cruz
CXCL14	ab137541		Abcam
IRα	sc-57344	AB_782041	Santa Cruz
IRβ (CT-3)	sc-57342	AB_784102	Santa Cruz
Jun (60A8)	9165	AB_2130165	Cell Signaling
P38 (D13E1)	8690	AB_10999090	Cell Signaling
pP38 (Thr180/Tyr182)	4511	AB_2139682	Cell Signaling
pIRβ (10C3)	sc-81500	AB_1125642	Santa Cruz
pJun(Ser63)	9261	AB_2130162	Cell Signaling
pErk1/2 (Thr202/Tyr204)	4370	AB_2315112	Cell Signaling
pJNK (Thr183/Tyr185)	9251	AB_331659	Cell Signaling
Tubulin	sc-5274	AB_2288090	Santa Cruz

### Statistical analysis

Bar graph results are expressed as mean ± SD. Fold change values and *p*-values for scRNA seq comparison were based on Wilcoxon Ranked Sum test generated by Seurat. The statistical analyses were performed using GraphPad Prism 6.0 software (GraphPad Software, San Diego). Student’s t-tests were used to compare between 2 groups. Two-way ANOVAs were used to compare multiple groups, post-test of Sidak, Bonferonni, or Tukey was used to compare between different groups. Differences with *p < 0.05, **p < 0.01, ***p < 0.001 were considered significant. (Table 1).

**Table 1:** Differentially expressed genes in Bace1-deleted astrocytes

Gene Symbol	Gene Name	avg_log2FC	-log10(p_val)	Function	Citation
*Ttr*	*Transthyretin*	1.117156	132.9195	Amyloid related	[61]
*Cxcl14*	*C-x-c motif ligand 14*	3.061096	93.76337	Tau related, protein changes	[53]
*Sparc*	*Secreted Protein Acidic And Cysteine Rich*	0.76606	51.25117	Synaptic maintenance	[62]
*Npy*	*Neuropeptide Y*	1.82274	49.92431	Synaptic maintenance	[63]
*Itm2b*	*Integral membrane protein 2B*	0.46628	43.56427	Synaptic maintenance	[64]
*Igf1*	*Insulin growth factor 1*	1.500563	40.94406	Insulin or insulin growth factor signaling	[65]
*Ctsl*	*Cathepsin L*	0.807468	37.51631	Amyloid related	[66]
*Cav1*	*Caveolin 1*	1.235155	36.40204	Amyloid related, synaptic maintenance	[67, 68]
*Igfbp7*	*Insulin growth factor binding protein 7*	2.358861	32.96275	Insulin or insulin growth factor signaling	[69]
*Junb*	*Junb*	-0.40882	16.06157	AP-1 Transcription family	[56, 70]
*Aqp4*	*Aquaporin 4*	-0.40882	16.06157	Amyloid related	[71]
*Fos*	*Fos*	0.865464	13.75286	AP-1 Transcription family	[56, 70]
*Clu*	*Clusterin*	0.267696	6.670486	Amyloid related, protein changes	[27, 51, 72]
*Timp3*	*TIMP Metallopeptidase Inhibitor 3*	0.338117	6.511402	Amyloid related	[73]
*Jun*	*Jun*	0.583823	4.81512	AP-1 Transcription family	[56, 70]

## Supplementary Information

**Additional file 1: Supplemental Figure S1.** Quality control measure of *Bace1*^*-/-*^ and *Bace1*^*+/+*^ scRNAseq. (A) Violin plots of data set quality control measurement for nFeature_RNA, nCount_RNA, and percent.mt generated from each ACSA2+-enriched sample. Samples 1a, 2a, 3a are from *Bace1*^*+/+*^ mice, while samples 4a, 5a, 6a are from *Bace1*^*-/-*^. Filtered cutoff points were set at GEMS containing >1,000 identified genes, <25,000 read counts, and <20% mitochondrial RNA. (B) Visualization of UMAP dimension plot with identified cell type clusters from *Bace1*^*-/-*^ and *Bace1*^*+/+*^ scRNAseq. R Astrocytes refers reactive astrocytes, and OPC stands for oligodendrocyte precursor cells. The visible difference is the increase in the R Astrocyte cluster in *Bace1*^*-/-*^ samples. **Supplemental Figure S2. **Quality control measure of *5xFAD;Bace1*^*fl/fl*^*;UBC-creER* and *5xFAD;Bace1*^*fl/fl*^*. *Quality control measured for nFeature_RNA, nCount_RNA, and percent.mt generated from pooled ACSA2+-enriched samples from 5xFAD;Bace1^fl/fl^;UBC-creER (Sample 1A) and 5xFAD;Bace1^fl/fl^ (Sample 3a) Filtered cutoff points were set at GEMS containing >1,000 identified genes, <25,000 read counts, and <20% mitochondrial RNA. **Supplemental Figure S3.** Validation of siRNA *Clu* knockdown. (A) Western blot of WT primary astrocytes treated with either 80, 40, 20, 10 pmol of *Clu* siRNA or 80 pmol of control scrambled siRNA. Images indicate major bands for CLU and actin. (B) CLU band intensity normalized to actin. We noted that 80 pmol of *Clu* siRNA resulted in an approximately 50% decrease in LU levels compared to control siRNA. **Supplemental Figure S4.** Targeted astrocytic deletion of Bace1 increases Aβ clearance. Representative images from Thioflavin-S staining of amyloid plaques from fixed saggital brain sections of *5xFAD;Bace1*^*fl/fl*^*;Gfap-cre* and *5xFAD;Bace1*^*fl/fl*^. Insets highlight hippocampal and cortical regions that are presented in Fig. 9A. **Supplemental Table 1.** List of differentially expressed genes of *Bace1*^*-/-*^ reactive astrocytes. **Supplemental Table 2.** List of differentially expressed genes of *5xFAD;Bace1*^*fl/fl*^*;UBC-creER *reactive astrocytes.

## Data Availability

All original data presented in the paper will be made available for reviews when needed. Research materials will be also made available when it is required. The GEO entry for the scRNA seq data: GSE230116.

## Abbreviations

APP: Amyloid precursor protein
Bace1: β-site APP cleaving enzyme
Aβ: Amyloid peptides
GFAP: Glial fibrillary acidic protein
ApoE: apolipoprotein E
Clu: Clusterin
CXCL14: C-X-C Motif Chemokine Ligand 14
IR: Insulin receptor
CTF: C-terminal fragment
ScRNA-seq: Single cell RNA sequencing
tSNE: T-Distributed Stochastic Neighbor Embedding
MAPK: Mitogen-activated protein kinase
Jun: Jun proto-oncogene
P38: 38 KDa mitogen-activated protein kinases
Erk: Extracellular signal-regulated kinases
Kl: Klotho
IDE: Insulin-degrading enzyme
Lamp2: Lysosomal associated membrane protein-2
Cre: Cre-recombinase

## References

[1] Corriveau RA (2017). Alzheimer's Disease-Related Dementias Summit 2016: National research priorities. Neurology.

[2] Golde TE (2022). Alzheimer's disease - the journey of a healthy brain into organ failure. Mol Neurodegener.

[3] Long JM, Holtzman DM (2019). Alzheimer disease: an update on pathobiology and treatment strategies. Cell.

[4] Jack CR (2018). NIA-AA Research Framework: toward a biological definition of Alzheimer's disease. Alzheimers Dement.

[5] Yan R, Vassar R (2014). Targeting the beta secretase BACE1 for Alzheimer's disease therapy. Lancet Neurol.

[6] McDade E, et al. The case for low-level BACE1 inhibition for the prevention of Alzheimer disease. Nat Rev Neurol, 2021;17(11):703–14. 10.1038/s41582-021-00545-1 34548654

[7] Egan MF (2019). Further analyses of the safety of verubecestat in the phase 3 EPOCH trial of mild-to-moderate Alzheimer's disease. Alzheimers Res Ther.

[8] Sur C (2020). BACE inhibition causes rapid, regional, and non-progressive volume reduction in Alzheimer's disease brain. Brain.

[9] Novak G (2020). Long-term safety and tolerability of atabecestat (JNJ-54861911), an oral BACE1 inhibitor, in early Alzheimer's disease spectrum patients: a randomized, double-blind, placebo-controlled study and a two-period extension study. Alzheimers Res Ther.

[10] Das B, et al. BACE1 controls synaptic function through modulating release of synaptic vesicles. Mol Psychiatry, 2021;26(11):6394–410. 10.1038/s41380-021-01166-2 PMC8760050 34158621

[11] Ou-Yang MH (2018). Axonal organization defects in the hippocampus of adult conditional BACE1 knockout mice. Sci Transl Med.

[12] Wang H (2008). BACE1 knock-outs display deficits in activity-dependent potentiation of synaptic transmission at mossy fiber to CA3 synapses in the hippocampus. J Neurosci.

[13] Wang H (2010). Mossy fiber long-term potentiation deficits in BACE1 knock-outs can be rescued by activation of alpha7 nicotinic acetylcholine receptors. J Neurosci.

[14] Lombardo S (2019). BACE1 partial deletion induces synaptic plasticity deficit in adult mice. Sci Rep.

[15] Hu X (2018). BACE1 deletion in the adult mouse reverses preformed amyloid deposition and improves cognitive functions. J Exp Med.

[16] Zhu K (2018). Consequences of Pharmacological BACE Inhibition on Synaptic Structure and Function. Biol Psychiatry.

[17] Hampel H (2021). The β-Secretase BACE1 in Alzheimer's Disease. Biol Psychiatry.

[18] McDade E (2021). The informed road map to prevention of Alzheimer Disease: A call to arms. Mol Neurodegener.

[19] Selkoe DJ, Hardy J (2016). The amyloid hypothesis of Alzheimer's disease at 25 years. EMBO Mol Med.

[20] Zhao J, O'Connor T, Vassar R (2011). The contribution of activated astrocytes to Aβ production: implications for Alzheimer's disease pathogenesis. J Neuroinflammation.

[21] Chacón-Quintero MV (2021). Beta-Secretase 1 Underlies Reactive Astrocytes and Endothelial Disruption in Neurodegeneration. Front Cell Neurosci.

[22] Hu X (2013). BACE1 regulates hippocampal astrogenesis via the Jagged1-Notch pathway. Cell Rep.

[23] Tasic B (2018). Shared and distinct transcriptomic cell types across neocortical areas. Nature.

[24] Wang M (2022). Guidelines for bioinformatics of single-cell sequencing data analysis in Alzheimer's disease: review, recommendation, implementation and application. Mol Neurodegener.

[25] Liddelow SA (2017). Neurotoxic reactive astrocytes are induced by activated microglia. Nature.

[26] Bettens K (2015). Reduced secreted clusterin as a mechanism for Alzheimer-associated CLU mutations. Mol Neurodegener.

[27] Wojtas AM (2020). Astrocyte-derived clusterin suppresses amyloid formation in vivo. Mol Neurodegener.

[28] Stine WB (2011). Preparing synthetic Aβ in different aggregation states. Methods Mol Biol.

[29] Bettegazzi B (2011). β-Secretase activity in rat astrocytes: translational block of BACE1 and modulation of BACE2 expression. Eur J Neurosci.

[30] Herdegen T, Waetzig V (2001). AP-1 proteins in the adult brain: facts and fiction about effectors of neuroprotection and neurodegeneration. Oncogene.

[31] Raivich G, Behrens A (2006). Role of the AP-1 transcription factor c-Jun in developing, adult and injured brain. Prog Neurobiol.

[32] Meakin PJ (2018). The beta secretase BACE1 regulates the expression of insulin receptor in the liver. Nat Commun.

[33] Garcia AD (2004). GFAP-expressing progenitors are the principal source of constitutive neurogenesis in adult mouse forebrain. Nat Neurosci.

[34] Oakley H (2006). Intraneuronal beta-amyloid aggregates, neurodegeneration, and neuron loss in transgenic mice with five familial Alzheimer's disease mutations: potential factors in amyloid plaque formation. J Neurosci.

[35] Dislich B (2015). Label-free Quantitative Proteomics of Mouse Cerebrospinal Fluid Detects beta-Site APP Cleaving Enzyme (BACE1) Protease Substrates In Vivo. Mol Cell Proteomics.

[36] Hampel H, et al. The β-Secretase BACE1 in Alzheimer's Disease. Biol Psychiatry. 2020;89(8):745–56. 10.1016/j.biopsych.2020.02.001 PMC7533042 32223911

[37] Das B, Yan R (2019). A Close Look at BACE1 Inhibitors for Alzheimer's Disease Treatment. CNS Drugs.

[38] Yan R (2017). Physiological Functions of the beta-Site Amyloid Precursor Protein Cleaving Enzyme 1 and 2. Front Mol Neurosci.

[39] Wyss-Coray T (2003). Adult mouse astrocytes degrade amyloid-beta in vitro and in situ. Nat Med.

[40] Vekrellis K (2000). Neurons regulate extracellular levels of amyloid beta-protein via proteolysis by insulin-degrading enzyme. J Neurosci.

[41] Dorfman VB (2010). Differential cerebral deposition of IDE and NEP in sporadic and familial Alzheimer's disease. Neurobiol Aging.

[42] Matsubara E (1996). Apolipoprotein J and Alzheimer's amyloid β solubility. Biochem J.

[43] Nuutinen T (2007). Amyloid-β 1–42 induced endocytosis and clusterin/apoJ protein accumulation in cultured human astrocytes. Neurochem Int.

[44] Nelson AR, Sagare AP, Zlokovic BV (2017). Role of clusterin in the brain vascular clearance of amyloid-β. Proc Natl Acad Sci U S A.

[45] Ries M, Sastre M (2016). Mechanisms of Aβ Clearance and Degradation by Glial Cells. Front Aging Neurosci.

[46] Perez-Nievas BG, Serrano-Pozo A (2018). Deciphering the Astrocyte Reaction in Alzheimer’s Disease. Front Aging Neurosci.

[47] Soreq L (2017). Major Shifts in Glial Regional Identity Are a Transcriptional Hallmark of Human Brain Aging. Cell Rep.

[48] Boisvert MM (2018). The Aging Astrocyte Transcriptome from Multiple Regions of the Mouse Brain. Cell Rep.

[49] Harold D (2009). Genome-wide association study identifies variants at CLU and PICALM associated with Alzheimer's disease. Nat Genet.

[50] Lambert JC (2009). Genome-wide association study identifies variants at CLU and CR1 associated with Alzheimer's disease. Nat Genet.

[51] Foster EM (2019). Clusterin in Alzheimer's Disease: Mechanisms, Genetics, and Lessons From Other Pathologies. Front Neurosci.

[52] Rahman MM, Lendel C (2021). Extracellular protein components of amyloid plaques and their roles in Alzheimer's disease pathology. Mol Neurodegener.

[53] Chung J (2018). Genome-wide association study of Alzheimer's disease endophenotypes at prediagnosis stages. Alzheimers Dement.

[54] Wojtas AM (2017). Loss of clusterin shifts amyloid deposition to the cerebrovasculature via disruption of perivascular drainage pathways. Proc Natl Acad Sci U S A.

[55] Chen F (2021). Clusterin secreted from astrocyte promotes excitatory synaptic transmission and ameliorates Alzheimer's disease neuropathology. Mol Neurodegener.

[56] Singh N (2022). BACE-1 inhibition facilitates the transition from homeostatic microglia to DAM-1. Sci Adv.

[57] Singh N (2022). Targeted BACE-1 inhibition in microglia enhances amyloid clearance and improved cognitive performance. Sci Adv.

[58] Holt LM, Olsen ML (2016). Novel Applications of Magnetic Cell Sorting to Analyze Cell-Type Specific Gene and Protein Expression in the Central Nervous System. PLoS One.

[59] Chen X (2010). Jagged1 expression regulated by Notch3 and Wnt/beta-catenin signaling pathways in ovarian cancer. Oncotarget.

[60] Schildge S (2013). Isolation and culture of mouse cortical astrocytes. J Vis Exp.

[61] Li X (2013). Mechanisms of transthyretin inhibition of β-amyloid aggregation in vitro. J Neurosci.

[62] Kucukdereli H (2011). Control of excitatory CNS synaptogenesis by astrocyte-secreted proteins Hevin and SPARC. Proc Natl Acad Sci U S A.

[63] Ramamoorthy P, Whim MD (2008). Trafficking and fusion of neuropeptide Y-containing dense-core granules in astrocytes. J Neurosci.

[64] Biundo F (2015). Interaction of ApoE3 and ApoE4 isoforms with an ITM2b/BRI2 mutation linked to the Alzheimer disease-like Danish dementia: Effects on learning and memory. Neurobiol Learn Mem.

[65] Westwood AJ (2014). Insulin-like growth factor-1 and risk of Alzheimer dementia and brain atrophy. Neurology.

[66] Klein DM, Felsenstein KM, Brenneman DE (2009). Cathepsins B and L differentially regulate amyloid precursor protein processing. J Pharmacol Exp Ther.

[67] Kang MJ (2006). Caveolin-1 upregulation in senescent neurons alters amyloid precursor protein processing. Exp Mol Med.

[68] Wang S (2021). Synapsin-caveolin-1 gene therapy preserves neuronal and synaptic morphology and prevents neurodegeneration in a mouse model of AD. Mol Ther Methods Clin Dev.

[69] Agbemenyah HY (2014). Insulin growth factor binding protein 7 is a novel target to treat dementia. Neurobiol Dis.

[70] Wei R (2022). ceRNA Network Analysis Reveals AP-1 Tanscription Factor Components as Potential Biomarkers for Alzheimer's Disease. Curr Alzheimer Res.

[71] Iliff JJ (2012). A paravascular pathway facilitates CSF flow through the brain parenchyma and the clearance of interstitial solutes, including amyloid β. Sci Transl Med.

[72] Oh SB (2019). Clusterin contributes to early stage of Alzheimer's disease pathogenesis. Brain Pathol.

[73] Hoe HS (2007). The metalloprotease inhibitor TIMP-3 regulates amyloid precursor protein and apolipoprotein E receptor proteolysis. J Neurosci.

