# Supplementary material for: BACE1 regulates expression of Clusterin in astrocytes for enhancing clearance of β-amyloid peptides
Source: Mol Neurodegener. 2023 May 4;18:31. doi: 10.1186/s13024-023-00611-w (PMC10161466; doi:10.1186/s13024-023-00611-w)
Supplement: Supplementary file 1 — Additional file 1: Supplemental Figure S1. Quality control measure of Bace1-/- and Bace1+/+ scRNAseq. (A) Violin plots of data set quality control measurement for nFeature_RNA, nCount_RNA, and percent.mt generated from each ACSA2+-enriched sample. Samples 1a, 2a, 3a are from Bace1+/+ mice, while samples 4a, 5a, 6a are from Bace1-/-. Filtered cutoff points were set at GEMS containing >1,000 identified genes, <25,000 read counts, and <20% mitochondrial RNA. (B) Visualization of UMAP dimension plot with identified cell type clusters from Bace1-/- and Bace1+/+ scRNAseq. R Astrocytes refers reactive astrocytes, and OPC stands for oligodendrocyte precursor cells. The visible difference is the increase in the R Astrocyte cluster in Bace1-/- samples. Supplemental Figure S2. Quality control measure of 5xFAD;Bace1fl/fl;UBC-creER and 5xFAD;Bace1fl/fl. Quality control measured for nFeature_RNA, nCount_RNA, and percent.mt generated from pooled ACSA2+-enriched samples from 5xFAD;Bace1fl/fl;UBC-creER (Sample 1A) and 5xFAD;Bace1fl/fl (Sample 3a) Filtered cutoff points were set at GEMS containing >1,000 identified genes, <25,000 read counts, and <20% mitochondrial RNA. Supplemental Figure S3. Validation of siRNA Clu knockdown. (A) Western blot of WT primary astrocytes treated with either 80, 40, 20, 10 pmol of Clu siRNA or 80 pmol of control scrambled siRNA. Images indicate major bands for CLU and actin. (B) CLU band intensity normalized to actin. We noted that 80 pmol of Clu siRNA resulted in an approximately 50% decrease in LU levels compared to control siRNA. Supplemental Figure S4. Targeted astrocytic deletion of Bace1 increases Aβ clearance. Representative images from Thioflavin-S staining of amyloid plaques from fixed saggital brain sections of 5xFAD;Bace1fl/fl;Gfap-cre and 5xFAD;Bace1fl/fl. Insets highlight hippocampal and cortical regions that are presented in Fig. 9A. Supplemental Table 1. List of differentially expressed genes of Bace1-/- reactive astrocytes. Supplemental [file 13024_2023_611_MOESM1_ESM.zip › Supplementary Table 1.pdf]

	avg_log2FC	-log10(p_val)
Rpl29	0.921959	1.73E+02
Gm42418	-0.46154	1.63E+02
Ttr	0.990196	1.33E+02
Cxcl14	2.843545	9.38E+01
Lars2	-0.48738	7.27E+01
Serpina3n	1.176561	6.46E+01
Vtn	1.210359	6.08E+01
Foxd3	0.60078	5.83E+01
Frzb	0.729411	5.75E+01
Ptgds	0.743744	5.74E+01
Fxyd6	0.625237	5.30E+01
Sparc	0.683906	5.13E+01
Npy	1.554926	4.99E+01
Kctd12	1.307231	4.87E+01
Clca3a1	0.767453	4.80E+01
Sat1	0.615184	4.59E+01
Hmgcs2	0.548202	4.44E+01
Itm2b	0.446662	4.36E+01
Nr2f2	0.674504	4.15E+01
Akap12	0.343431	4.13E+01
Igf1	0.412032	4.09E+01
Igfbp4	0.448316	4.07E+01
Edil3	0.668597	4.06E+01
Cebpd	0.853598	4.03E+01
Fbln2	0.452453	3.96E+01
Sox10	0.353458	3.95E+01
Tmod2	0.515031	3.95E+01
Wnt6	0.309268	3.92E+01
Fkbp5	0.264721	3.89E+01
Brinp3	0.44296	3.85E+01
Matn4	0.454471	3.82E+01
Ctsl	0.637254	3.75E+01
Lurap1l	0.264403	3.74E+01
Apcdd1	0.501647	3.66E+01
Cav1	0.259447	3.64E+01
Col11a1	0.373332	3.58E+01
Gsn	0.930667	3.57E+01
Mt3	-0.30854	3.56E+01
Hey2	0.421733	3.46E+01
Tubb2b	-0.53482	3.43E+01
Hpgd	0.270112	3.38E+01
Nell2	0.32709	3.38E+01
Prrg3	0.330628	3.36E+01

Gpsm2	0.278619	3.35E+01
Mest	0.470153	3.30E+01
Igfbp7	0.907901	3.30E+01
Agt	0.707266	3.27E+01
Erbb3	0.288173	3.21E+01
Rbm3	0.452291	3.18E+01
Angptl4	0.402963	3.13E+01
Cryab	0.522876	3.12E+01
Spock2	0.397224	3.12E+01
Gm29521	0.536285	3.11E+01
2900052N01Rik	0.752225	3.11E+01
Tsc22d3	0.509386	3.08E+01
Ramp2	0.392614	3.06E+01
Spidr	0.599611	3.05E+01
Aspa	0.520035	3.05E+01
Bgn	0.395388	3.01E+01
Pmepa1	0.633423	2.96E+01
Id4	-0.39408	2.95E+01
Sult1a1	0.356844	2.86E+01
Eno1	-0.55478	2.83E+01
Aatk	0.396955	2.82E+01
Vstm4	0.275633	2.76E+01
Gas7	0.36579	2.75E+01
Plp1	0.688931	2.66E+01
Ahcyl2	0.548579	2.64E+01
Usp54	0.420012	2.63E+01
Matn2	0.295047	2.61E+01
Tax1bp3	0.303568	2.53E+01
Tmsb4x	0.663308	2.52E+01
Sox2	-0.35195	2.47E+01
Abca8a	0.332295	2.46E+01
Lhfp	0.321681	2.46E+01
Hopx	-0.5717	2.45E+01
Mfsd2a	0.33224	2.39E+01
Vim	0.477634	2.37E+01
Etnppl	0.363226	2.37E+01
Plce1	0.399726	2.36E+01
Ifitm3	0.489503	2.34E+01
Gpt2	0.303892	2.31E+01
Ddit4	0.28553	2.30E+01
Gatm	0.36863	2.30E+01
Cd200	0.27418	2.16E+01
Pabpc1	0.291423	2.15E+01
Fabp5	-0.29733	2.10E+01

Pik3r1	0.325468	2.08E+01
Lpar1	0.345659	2.03E+01
Fam3c	0.393758	2.01E+01
Nme7	0.316736	2.01E+01
Enpp2	0.314133	1.96E+01
Sdc4	0.321201	1.95E+01
Ucp2	0.343987	1.86E+01
Tspan15	0.360608	1.85E+01
Ifitm2	0.420149	1.85E+01
Ugp2	-0.43734	1.84E+01
Gpm6a	-0.36345	1.84E+01
Selenop	0.708668	1.84E+01
Cpne8	0.276451	1.82E+01
Plat	0.38037	1.80E+01
Scd1	0.420117	1.75E+01
Slc38a2	0.319134	1.75E+01
Junb	0.454613	1.75E+01
Cdkn1a	0.375135	1.74E+01
Nupr1	0.306821	1.72E+01
Gm9925	0.29837	1.71E+01
Plpp3	-0.38866	1.69E+01
Rorb	-0.48996	1.68E+01
Fuca1	0.321956	1.63E+01
Ncam1	0.648074	1.62E+01
Egr1	0.358142	1.62E+01
Aqp4	-0.35239	1.61E+01
Ralgps2	0.379984	1.59E+01
Fmo1	0.306995	1.57E+01
Pros1	0.277833	1.56E+01
Abhd12	0.252901	1.55E+01
Malat1	0.276179	1.55E+01
Nrxn1	-0.37656	1.54E+01
Sdc3	0.264836	1.52E+01
Ddah1	-0.40369	1.51E+01
Cirbp	0.254184	1.50E+01
Tmem47	-0.25609	1.48E+01
Slc22a17	0.285261	1.48E+01
Sorbs1	0.307678	1.43E+01
Arpc1b	0.27567	1.43E+01
Tmsb10	0.729566	1.42E+01
Sfrp1	-0.38871	1.42E+01
Cd9	0.256722	1.41E+01
S1pr1	-0.40627	1.41E+01
Mfap3l	0.255787	1.39E+01

Cd59a	0.391797	1.39E+01
Fos	0.509915	1.38E+01
Ccdc153	-0.953	1.36E+01
Mrpl41	0.399726	1.34E+01
Marcks	0.426488	1.33E+01
Apoc1	0.433234	1.29E+01
Mboat2	0.343038	1.28E+01
Dnajc1	0.266851	1.28E+01
Cyp2j6	0.299903	1.27E+01
Lxn	-0.38653	1.26E+01
Tst	-0.41181	1.25E+01
F3	0.282198	1.24E+01
Glud1	-0.33739	1.23E+01
Klf9	0.255484	1.22E+01
Arhgap5	-0.28486	1.20E+01
Pantr1	-0.29883	1.15E+01
Ntm	-0.33548	1.07E+01
Cxcl13	-0.28991	1.05E+01
Lix1	-0.44171	1.03E+01
Dbp	-0.32586	1.00E+01
Mfge8	-0.40216	1.00E+01
Ncald	0.277343	1.00E+01
Sgk1	0.252553	1.00E+01
Cldn10	-0.32008	9.68E+00
Phyhipl	-0.32091	9.60E+00
Alas1	-0.28897	9.45E+00
Cd47	0.251892	9.26E+00
Cspg5	-0.3621	8.97E+00
Nr1d1	-0.29353	8.48E+00
Neat1	0.304007	8.26E+00
Pou3f3	-0.27619	7.92E+00
Lhx2	-0.31377	7.82E+00
Fxyd1	0.347316	7.82E+00
Rgcc	0.32211	7.75E+00
Txnip	0.425769	7.63E+00
Rgs2	0.463413	7.55E+00
Arap2	-0.28154	7.37E+00
Adcy2	-0.26934	7.22E+00
Nbl1	0.30352	7.18E+00
Mt1	0.331612	7.08E+00
Clu	0.266635	6.67E+00
Timp3	0.258378	6.51E+00
Sash1	0.460557	6.39E+00
Slc1a2	-0.26207	6.35E+00

Hist1h4d	-0.30169	6.28E+00
Baalc	-0.28383	6.27E+00
Id2	-0.37267	6.18E+00
Ifi27	0.283058	6.14E+00
Gnao1	-0.27221	6.04E+00
Il18	-0.34924	5.99E+00
Glrb	-0.2933	5.84E+00
Hist1h1e	-0.32313	5.76E+00
Mgst1	0.322074	5.66E+00
Mgll	-0.3524	5.10E+00
Dbx2	-0.31581	5.09E+00
Fam13c	-0.29263	4.85E+00
Jun	0.419731	4.82E+00
AW047730	-0.36503	4.64E+00
Lmo4	0.270047	4.46E+00
Tril	-0.30162	4.04E+00
Idi1	-0.30766	3.72E+00
Slc15a2	-0.25345	3.51E+00
Car2	-0.37308	3.18E+00
Ctnnd2	-0.25067	2.95E+00
Mns1	-0.42898	2.89E+00
Tubb2a	-0.32319	2.74E+00
Il33	-0.27419	2.68E+00
Fabp7	1.561143	2.62E+00
Myo6	-0.28435	2.47E+00
Ppp2r2b	-0.25928	1.68E+00
Asrgl1	-0.26181	9.08E-01
