# Supplementary material for: BACE1 regulates expression of Clusterin in astrocytes for enhancing clearance of β-amyloid peptides
Source: Mol Neurodegener. 2023 May 4;18:31. doi: 10.1186/s13024-023-00611-w (PMC10161466; doi:10.1186/s13024-023-00611-w)
Supplement: Supplementary file 1 — Additional file 1: Supplemental Figure S1. Quality control measure of Bace1-/- and Bace1+/+ scRNAseq. (A) Violin plots of data set quality control measurement for nFeature_RNA, nCount_RNA, and percent.mt generated from each ACSA2+-enriched sample. Samples 1a, 2a, 3a are from Bace1+/+ mice, while samples 4a, 5a, 6a are from Bace1-/-. Filtered cutoff points were set at GEMS containing >1,000 identified genes, <25,000 read counts, and <20% mitochondrial RNA. (B) Visualization of UMAP dimension plot with identified cell type clusters from Bace1-/- and Bace1+/+ scRNAseq. R Astrocytes refers reactive astrocytes, and OPC stands for oligodendrocyte precursor cells. The visible difference is the increase in the R Astrocyte cluster in Bace1-/- samples. Supplemental Figure S2. Quality control measure of 5xFAD;Bace1fl/fl;UBC-creER and 5xFAD;Bace1fl/fl. Quality control measured for nFeature_RNA, nCount_RNA, and percent.mt generated from pooled ACSA2+-enriched samples from 5xFAD;Bace1fl/fl;UBC-creER (Sample 1A) and 5xFAD;Bace1fl/fl (Sample 3a) Filtered cutoff points were set at GEMS containing >1,000 identified genes, <25,000 read counts, and <20% mitochondrial RNA. Supplemental Figure S3. Validation of siRNA Clu knockdown. (A) Western blot of WT primary astrocytes treated with either 80, 40, 20, 10 pmol of Clu siRNA or 80 pmol of control scrambled siRNA. Images indicate major bands for CLU and actin. (B) CLU band intensity normalized to actin. We noted that 80 pmol of Clu siRNA resulted in an approximately 50% decrease in LU levels compared to control siRNA. Supplemental Figure S4. Targeted astrocytic deletion of Bace1 increases Aβ clearance. Representative images from Thioflavin-S staining of amyloid plaques from fixed saggital brain sections of 5xFAD;Bace1fl/fl;Gfap-cre and 5xFAD;Bace1fl/fl. Insets highlight hippocampal and cortical regions that are presented in Fig. 9A. Supplemental Table 1. List of differentially expressed genes of Bace1-/- reactive astrocytes. Supplemental [file 13024_2023_611_MOESM1_ESM.zip › Supplementary Table 2.pdf]

	avg_log2FC	-log10(p_value)
Gm42418	3.531962352	1.29E+02
Ttr	-3.658643957	1.09E+02
AY036118	3.586328392	1.09E+02
Eid1	-1.360338006	1.06E+02
Sparcl1	1.842070016	5.96E+01
Mt2	1.633181563	5.93E+01
Gstm1	1.510387911	5.66E+01
Mt1	1.55487183	5.63E+01
Dbi	1.604200368	5.55E+01
Pcolce	-1.393525466	5.52E+01
Calm1	1.227960562	5.46E+01
Bsg	-1.845259768	5.39E+01
Nnat	1.651057044	5.31E+01
Fabp5	1.494972827	5.29E+01
Mt3	1.304150416	5.12E+01
Tmem47	1.258415202	4.88E+01
Perp	-1.343419869	4.79E+01
Prdx6	1.225879253	4.77E+01
Tuba1a	1.34684209	4.64E+01
Wfdc2	-1.329157071	4.58E+01
Gpr37l1	1.199506046	4.52E+01
Fam183b	1.660431588	4.48E+01
Ahcyl2	-1.403097734	4.40E+01
Stk39	-1.288718477	4.31E+01
Ccdc153	1.706980796	4.28E+01
Gm10076	-0.992638308	4.26E+01
Ifitm2	-1.108362941	4.20E+01
Enkur	1.501399431	4.17E+01
Scg3	1.206106175	4.14E+01
Sntn	1.535537628	4.14E+01
Nuf2	1.282091321	4.12E+01
Sox2	1.127990989	4.12E+01
Islr	-1.802770142	4.10E+01
Mlc1	1.132061414	4.04E+01
Tppp3	1.400558901	4.01E+01
Meig1	1.577579474	3.93E+01
Rarres2	1.63001793	3.92E+01
Hmgb1	0.933601258	3.90E+01
Serpine2	1.078935569	3.80E+01
S100b	1.178229872	3.79E+01
Tmem212	1.510577945	3.77E+01
Fez1	0.952741154	3.76E+01
Car14	-0.862856156	3.74E+01
Kcne2	-1.224342051	3.72E+01
Ly6e	-1.17951455	3.71E+01
1700012B09Rik	1.437243688	3.68E+01

Kl	-1.764946999	3.67E+01
Npr3	-0.930594909	3.64E+01
Slc25a18	0.959329656	3.62E+01
Aqp4	1.151619996	3.60E+01
Col8a1	-0.913578909	3.58E+01
Psph	1.196718526	3.56E+01
Riiad1	1.275038523	3.54E+01
Plp1	0.557671403	3.53E+01
Ace	-1.068643506	3.52E+01
Dnali1	1.204123954	3.50E+01
Ucp2	-1.151366001	3.50E+01
Slc5a6	-1.343517495	3.49E+01
Kcnj13	-1.802915242	3.47E+01
Eef1a1	-0.853326293	3.47E+01
Tmem86a	-0.899650531	3.45E+01
Arhgap5	0.991499356	3.45E+01
Gm16006	1.07706794	3.45E+01
Ivns1abp	1.025368692	3.44E+01
Slc4a10	-1.880885628	3.42E+01
Cfap161	1.176730493	3.42E+01
Lgals1	-1.079012952	3.41E+01
Ptprz1	1.287698343	3.38E+01
Emb	-1.34430829	3.37E+01
Myl6	-1.111496289	3.36E+01
Cdkn1c	-1.432407527	3.36E+01
Pla2g7	1.391200463	3.35E+01
Sulf1	-0.957672065	3.35E+01
Mtch1	-1.113688817	3.28E+01
Chd3	-0.723489249	3.27E+01
Gm10714	1.07934991	3.26E+01
Ntrk2	0.835015606	3.26E+01
Ldha	-0.872643915	3.24E+01
Eef2	-0.78767849	3.21E+01
Cpe	1.097794412	3.21E+01
Got1l1	1.032100401	3.21E+01
Iqca	1.087496833	3.18E+01
Hdc	1.207414933	3.18E+01
1110017D15Rik	1.423722357	3.16E+01
1700001C02Rik	1.197890294	3.14E+01
Prlr	-1.760077072	3.13E+01
Abcg2	-1.301013622	3.12E+01
Mgp	-4.295632523	3.11E+01
Igf2	-1.191431505	3.09E+01
Dynlrb2	1.319082886	3.09E+01
Slc12a2	-1.118643882	3.08E+01
Vamp8	-1.160414985	3.07E+01
Atp1b1	-1.457235453	3.06E+01

Slc1a2	1.90489808	3.05E+01
Gpm6b	1.077752602	3.05E+01
Hsp90aa1	1.360332257	3.03E+01
Rbp1	-1.330986536	3.02E+01
Ascc1	1.399704832	3.01E+01
Aes	-0.829903731	3.00E+01
Rbm47	-0.696306092	3.00E+01
Calb1	1.390195136	2.99E+01
Iqcg	1.054116647	2.98E+01
Fam216b	0.960801439	2.97E+01
Akr1a1	-0.9972415	2.96E+01
Dmkn	1.069840226	2.93E+01
Car12	-0.88233219	2.92E+01
Spa17	1.172778531	2.90E+01
Gm867	0.971056459	2.89E+01
Cox8b	-1.888272954	2.88E+01
Ctsd	-1.268887324	2.87E+01
Ptgis	-0.766504284	2.83E+01
Dgat2	0.957937819	2.83E+01
Ccdc33	0.992245266	2.83E+01
Vat1l	-0.910653751	2.82E+01
1700007G11Rik	0.928923718	2.82E+01
Cpq	-0.801923801	2.82E+01
Cfap126	1.260033122	2.81E+01
Efcab10	1.14044177	2.80E+01
Ptgds	-2.962979475	2.80E+01
Dthd1	0.828124218	2.79E+01
Rsph1	1.315609274	2.78E+01
Ttyh1	0.95769709	2.78E+01
Slc1a3	1.276941424	2.78E+01
Tctex1d4	0.980966078	2.77E+01
Fxyd5	-2.994576551	2.76E+01
Stra6	-0.6146514	2.74E+01
Apoe	1.062831073	2.74E+01
Ccdc121	0.848803696	2.72E+01
Anxa5	1.035394842	2.72E+01
Clic6	-1.471748579	2.70E+01
Crygn	1.10504909	2.70E+01
Ppp1r32	0.807505871	2.70E+01
Stoml3	0.980901503	2.69E+01
Rhoc	-0.738667231	2.69E+01
Txn1	0.967646048	2.69E+01
Slc29a4	-0.744225872	2.69E+01
Atp1a2	1.237226992	2.68E+01
Ramp1	0.87689207	2.68E+01
Nudt4	1.193072865	2.67E+01
Pvalb	1.066565034	2.66E+01

Gm11744	-0.885953462	2.66E+01
Lrrc48	1.018376691	2.66E+01
Prnp	0.800258666	2.65E+01
Cd24a	0.966503423	2.64E+01
Tuba1c	-0.483372432	2.64E+01
Cadm1	0.779247563	2.62E+01
Igfbp6	-1.978378703	2.62E+01
Cfap77	0.942811448	2.61E+01
Ifi27	-1.030877699	2.61E+01
Gm973	0.936562117	2.60E+01
Nov	-3.037996782	2.59E+01
Gm21149	0.97144599	2.58E+01
1700001L19Rik	0.887439244	2.57E+01
Igfbp7	-0.953676158	2.57E+01
Nt5dc2	-0.587994111	2.57E+01
Slc26a7	-1.224995678	2.57E+01
Polr3h	0.838212714	2.56E+01
Cdc42ep5	-0.623478239	2.55E+01
Vim	1.116292475	2.55E+01
Cdhr4	0.943521644	2.54E+01
Ak7	1.067734515	2.54E+01
Prr32	-0.79770175	2.54E+01
Lrrc74b	0.843674005	2.54E+01
Slc47a1	-1.809226578	2.54E+01
Hsp90ab1	0.837864689	2.53E+01
Ccdc146	0.967602339	2.53E+01
Id4	0.892145781	2.52E+01
4933434E20Rik	1.33822124	2.52E+01
Hepacam	0.761592212	2.51E+01
Cdr2	-0.715027458	2.51E+01
Zic1	-0.930599915	2.50E+01
Nudc	0.987478399	2.49E+01
Gm5617	0.921482703	2.49E+01
Acsl3	1.025457052	2.49E+01
Abcb1a	-1.00863725	2.49E+01
Wdr86	-0.580852463	2.48E+01
Col1a2	-1.694870235	2.47E+01
Rsph4a	0.95377489	2.46E+01
Fam166b	0.819521692	2.46E+01
Atox1	-1.145984711	2.46E+01
Stmnd1	0.836421193	2.45E+01
Hspa8	-0.507406493	2.45E+01
Ppp1r36	0.978056378	2.44E+01
Lrrc23	0.886063217	2.44E+01
Fzd4	-0.555769085	2.43E+01
Tubb5	-0.63998101	2.42E+01
Crip1	1.02506959	2.42E+01

Dab2	-0.576496134	2.42E+01
Sult1a1	-0.717700315	2.41E+01
Col6a2	-1.232484555	2.39E+01
Ramp2	-0.997199444	2.39E+01
Bgn	-1.386570841	2.38E+01
1700016K19Rik	0.934724076	2.38E+01
Dnah5	0.938945619	2.38E+01
Chchd10	0.807747836	2.38E+01
Tcn2	-0.85242225	2.37E+01
Ccdc34	0.881680215	2.37E+01
Nkain4	0.802307351	2.37E+01
Abracl	-0.44920636	2.35E+01
Nptn	-0.660445703	2.35E+01
Dmd	0.953611324	2.35E+01
Drc1	0.762848696	2.35E+01
Odf3b	0.873089146	2.35E+01
Spata24	0.919720954	2.34E+01
Emp3	-1.049136457	2.34E+01
Htr2c	-0.777546577	2.34E+01
Arpc1b	-0.901424419	2.34E+01
Deptor	-0.514770444	2.33E+01
Lrrc10b	0.708459715	2.32E+01
Meis2	0.749569994	2.32E+01
Gnas	-0.670366661	2.32E+01
Col6a1	-1.505701643	2.32E+01
Anxa3	-1.285678819	2.32E+01
Ccdc74a	0.823452469	2.31E+01
Kbtbd11	0.773023496	2.31E+01
Phldb2	-0.672286734	2.31E+01
Tmtc1	-0.761858474	2.31E+01
Elof1	1.111079448	2.30E+01
Mro	0.710928113	2.29E+01
Arhgdib	-0.74815221	2.29E+01
Slc4a5	-0.550254542	2.29E+01
Slc16a8	-0.639196702	2.28E+01
Morn5	0.894953412	2.28E+01
Tm4sf1	1.16553735	2.28E+01
Foxd1	-1.420222132	2.28E+01
Tspan11	-0.869026972	2.27E+01
Mapk10	0.673027767	2.27E+01
Hspa4l	0.849586963	2.25E+01
Fn1	-1.776506445	2.25E+01
6820408C15Rik	0.767593451	2.24E+01
Mns1	0.905585445	2.24E+01
Ltc4s	-0.811527012	2.24E+01
Mrc2	-1.190674539	2.23E+01
Med9os	0.683324455	2.23E+01

Hist1h4h	1.049379168	2.23E+01
Ogn	-1.106936265	2.23E+01
Thbd	-1.199510873	2.22E+01
1700024G13Rik	0.772677032	2.22E+01
Mid1ip1	0.782188028	2.21E+01
Pih1d2	0.849059667	2.21E+01
Capsl	0.960298356	2.21E+01
Car13	-0.802655895	2.20E+01
App	-0.890619506	2.20E+01
1700088E04Rik	0.901335173	2.20E+01
Fbln5	-0.429039324	2.19E+01
Fabp7	0.461168506	2.19E+01
Bmp4	-0.733247222	2.19E+01
Tmem72	-0.624399238	2.18E+01
Prrx2	-0.766079069	2.18E+01
Lars2	0.778898912	2.17E+01
Slc23a2	-1.235543122	2.17E+01
Bmp7	-0.4537665	2.16E+01
Cnp	0.567728136	2.16E+01
Pgrmc1	-0.71123593	2.16E+01
Scd2	0.783430906	2.15E+01
Timp4	0.873528475	2.15E+01
Sox9	0.785860811	2.14E+01
Tekt4	0.767252636	2.14E+01
Abca4	-0.47775952	2.14E+01
Map2	0.65428974	2.13E+01
Hemk1	-1.254921531	2.13E+01
Elmod1	0.749621331	2.12E+01
Foxc2	-0.751337802	2.12E+01
Spef2	0.931877408	2.12E+01
Slc16a6	-0.450561978	2.12E+01
Kcnj10	0.723446039	2.12E+01
Ostf1	-0.572201047	2.11E+01
Nrp1	-1.255008233	2.11E+01
Trabd2b	-0.614688006	2.11E+01
Rassf9	0.725916796	2.10E+01
Ccdc81	0.710155427	2.10E+01
Thbs1	-1.367588221	2.09E+01
Ppil6	0.77105504	2.09E+01
Ruvbl2	0.800227369	2.09E+01
Hspa2	0.802873614	2.09E+01
Bbox1	0.737710201	2.09E+01
Trpm3	-0.754761321	2.09E+01
Dnah12	0.927612167	2.08E+01
Arhgdig	0.895448493	2.08E+01
Smim14	-0.640754242	2.08E+01
Efcab1	0.915448701	2.08E+01

2410004P03Rik	0.886884502	2.08E+01
Ift43	0.849567234	2.08E+01
Cfap206	0.818726276	2.07E+01
Selenbp1	0.674113728	2.07E+01
Cbs	0.64973933	2.07E+01
Gm12326	0.798812938	2.07E+01
Zcchc18	0.748596853	2.06E+01
BC051019	0.613135869	2.06E+01
Npc2	-0.777569003	2.05E+01
Aldoc	1.691364999	2.05E+01
Wdr66	0.766267524	2.05E+01
Ndufa1	-1.121020807	2.05E+01
Chd3os	0.665324793	2.05E+01
Selm	-0.758553712	2.04E+01
Uap1l1	-0.430978816	2.03E+01
Sgk3	-0.548986322	2.03E+01
Lrrc51	0.86553113	2.02E+01
Wnt4	0.791907953	2.01E+01
Ctxn1	0.774155657	2.00E+01
Mmd2	0.910271926	2.00E+01
Thbs2	-0.487582979	1.99E+01
S100a6	1.074774906	1.99E+01
Catip	0.719122377	1.99E+01
Hpcal1	-0.314436665	1.99E+01
Kcnk2	0.636902688	1.99E+01
Mia	1.08399958	1.98E+01
Slc31a1	-0.742146598	1.97E+01
Ccdc39	0.862680487	1.97E+01
Kcnmb1	0.687911431	1.96E+01
Enpp2	-3.117704493	1.96E+01
Ccnd3	-0.62222782	1.96E+01
Lsr	-0.448711184	1.96E+01
Fam213a	0.673555882	1.96E+01
Pkdcc	-0.726904202	1.95E+01
Rgs22	0.672583642	1.94E+01
Sh3pxd2a	-0.969493816	1.94E+01
Ccdc170	0.61712047	1.94E+01
Mlf1	0.960430488	1.94E+01
Spag6l	0.813859394	1.94E+01
Cfap54	1.059086456	1.92E+01
Dnase2a	-0.444840011	1.92E+01
Macf1	0.98694103	1.92E+01
Pde5a	-0.459161316	1.92E+01
Ooep	-0.488192828	1.91E+01
Ninj1	-0.593418054	1.91E+01
Ctsc	-0.498319889	1.91E+01
Lrrc9	0.576832501	1.90E+01

Slc2a1	0.816418623	1.89E+01
Tpd52l1	-0.651442621	1.89E+01
St6galnac2	-0.3900797	1.89E+01
Tmem132b	0.62816239	1.89E+01
Spag17	0.75081056	1.88E+01
Ttc21a	0.663079721	1.88E+01
H2-DMb1	-0.556215803	1.87E+01
Ift27	0.754297141	1.87E+01
Atp6ap2	-0.802975203	1.87E+01
Lrrc36	0.710125678	1.87E+01
Akap14	0.66808623	1.87E+01
Hprt	-0.765509805	1.87E+01
H2-T23	-0.753545865	1.87E+01
Ccdc173	0.701463212	1.86E+01
Acta2	1.51302203	1.86E+01
Fndc3b	-0.387523598	1.85E+01
Ccdc180	0.738173828	1.85E+01
Plekhb1	0.663332813	1.85E+01
Syne1	0.989084559	1.84E+01
Bnc2	-0.496301552	1.84E+01
Ckap5	0.669741762	1.84E+01
Aqp1	-0.520697359	1.83E+01
Ckb	0.747496185	1.83E+01
Ly6h	0.690597946	1.83E+01
1810011O10Rik	-0.762331015	1.82E+01
Tsga10	0.714421467	1.82E+01
Frmpd1os	-0.483377214	1.82E+01
Morn3	0.681657121	1.81E+01
Slc9a2	-0.592043794	1.80E+01
Erich2	0.703982134	1.80E+01
Cfap100	0.635549649	1.80E+01
Fhl1	0.797866455	1.80E+01
Gm17455	0.745300406	1.80E+01
3300002A11Rik	0.727307209	1.80E+01
Ngef	0.602479472	1.79E+01
BC005624	0.844856012	1.79E+01
Cdh5	-0.493880144	1.78E+01
C1galt1c1	-0.526343719	1.78E+01
Tmem107	0.892073525	1.78E+01
Myb	0.568659981	1.78E+01
Sult1c2	-0.408611983	1.77E+01
Col12a1	-0.95803121	1.77E+01
Pacrg	0.719521424	1.76E+01
Ncam1	0.564130042	1.76E+01
Tubb4b	0.747165298	1.76E+01
Tspan15	0.545134779	1.76E+01
Plbd2	-0.438598268	1.76E+01

Khdrbs3	0.614555221	1.75E+01
Slco1a4	-0.483315088	1.75E+01
Foxj1	0.743468811	1.75E+01
Xbp1	-0.578604677	1.75E+01
Clec3b	0.732794161	1.74E+01
Zfp474	0.556728736	1.74E+01
Tbc1d9	-0.970019694	1.74E+01
Mpzl2	-0.500612087	1.73E+01
Lamp2	-0.753877331	1.73E+01
Fbxo2	0.876642348	1.72E+01
1700019G24Rik	0.644725441	1.72E+01
Naca	-0.609184854	1.72E+01
Timp1	-0.462950348	1.71E+01
Qk	0.799307485	1.71E+01
Cdhr3	0.720248586	1.71E+01
Acad8	-0.85408311	1.71E+01
Bok	0.689060617	1.71E+01
Adamtsl1	-0.878145945	1.71E+01
Trpv4	-0.351120137	1.71E+01
1700026L06Rik	0.811142381	1.71E+01
Ptma	0.596724206	1.71E+01
Prr29	0.690999089	1.71E+01
6330403K07Rik	0.399960024	1.71E+01
Foxc1	-0.851730503	1.71E+01
Strip2	-0.53483191	1.70E+01
Fap	-0.265242658	1.70E+01
Slc35g1	-0.508057778	1.70E+01
Slc25a3	-0.671759376	1.69E+01
Slc14a2	0.663440785	1.69E+01
Slc16a9	-0.682718011	1.69E+01
Cfh	-0.565508091	1.69E+01
Phactr2	-0.563856116	1.68E+01
Fjx1	0.76182997	1.68E+01
Barx2	0.631650332	1.68E+01
Ift88	0.649782804	1.68E+01
Chek2	0.787062324	1.68E+01
Cnn3	0.67799938	1.68E+01
Efemp1	-1.812903527	1.67E+01
Coq10b	-0.604068264	1.67E+01
Adgb	0.638040963	1.67E+01
Sh3bgrl	-0.63929398	1.67E+01
Adamtsl3	-0.909452087	1.67E+01
Six2	-0.519203287	1.66E+01
Sox1	0.534062521	1.66E+01
Akap12	-0.785958605	1.66E+01
Enah	0.662281722	1.65E+01
Hydin	0.810127814	1.65E+01

A2m	-0.671425632	1.65E+01
Hspe1	0.718970672	1.65E+01
Pttg1	0.715962997	1.65E+01
Ccdc30	0.627755615	1.64E+01
Ttc25	0.643667028	1.64E+01
Eya2	-0.545646743	1.64E+01
Ipo11	0.721912869	1.64E+01
Gpc3	-0.755653552	1.64E+01
Arsg	-0.686501613	1.63E+01
Crabp2	-0.672336606	1.62E+01
Col1a1	-1.533211848	1.62E+01
Crb3	-0.358969285	1.62E+01
Styxl1	0.519223282	1.62E+01
Cfap44	0.748807825	1.62E+01
Creg2	0.541323122	1.61E+01
Adamts20	0.61323166	1.60E+01
Tgfbi	-0.779084639	1.60E+01
Eea1	0.832274677	1.60E+01
Paip2	0.633755132	1.60E+01
H2-Aa	-1.139555501	1.59E+01
Mdh1b	0.591257838	1.59E+01
Cxcl14	1.07978735	1.59E+01
Foxp2	-0.747635003	1.59E+01
Armc3	0.605456765	1.59E+01
Fcgrt	-0.675963159	1.59E+01
Itm2a	-0.964139481	1.59E+01
Fam132a	-0.445470863	1.58E+01
Bphl	0.795567019	1.58E+01
Glrx3	-0.798177427	1.58E+01
Kcne4	-0.732281045	1.58E+01
Ccdc190	0.558733221	1.58E+01
Casc1	0.605656595	1.58E+01
Cxcl16	-0.383996814	1.57E+01
Cped1	-0.481288726	1.57E+01
Nr4a1	-1.125877281	1.57E+01
Twist1	-0.522794778	1.57E+01
Cfap52	0.703996016	1.57E+01
Pifo	0.805139198	1.56E+01
Gng5	0.593227334	1.56E+01
Rfx4	0.533961069	1.56E+01
Ppic	-0.489074054	1.56E+01
Pomgnt1	-0.538470791	1.56E+01
Tinagl1	-0.318751172	1.56E+01
Scnn1a	0.555412869	1.55E+01
Cfap45	0.71422294	1.55E+01
Ccpg1os	0.785103895	1.55E+01
Dnah6	0.824859032	1.55E+01

Atp2b3	-0.345327704	1.55E+01
Id1	-1.182684307	1.54E+01
Scrn1	0.54179005	1.54E+01
Emilin1	-0.667219883	1.54E+01
Dnah3	0.517857419	1.53E+01
Ccdc191	0.61040054	1.53E+01
Cep126	0.641602425	1.53E+01
Ptges3	0.602271509	1.53E+01
Khk	0.641285121	1.53E+01
Cacna1s	0.697203975	1.53E+01
4932443I19Rik	0.626851524	1.53E+01
Anpep	-0.543331938	1.52E+01
Ccdc113	0.772149216	1.52E+01
Pip5k1b	-0.562450165	1.52E+01
Uox	0.522748494	1.51E+01
4933407L21Rik	0.566407751	1.51E+01
Cul4b	-0.69492045	1.51E+01
Ankub1	0.521375145	1.51E+01
Cfap70	0.595039102	1.51E+01
Ttll3	0.658757743	1.51E+01
Tfpi	-0.360003384	1.51E+01
Fabp3	-0.447728296	1.51E+01
Car10	-0.426109491	1.51E+01
Samd9l	-0.458893685	1.50E+01
Gm11992	0.566013104	1.50E+01
FRMPD2	0.558047812	1.50E+01
Pltp	0.947668744	1.50E+01
Rdh5	-1.229528524	1.50E+01
Maf	-0.707601386	1.49E+01
Skil	-0.496454399	1.49E+01
Sowahc	-0.646797692	1.49E+01
Slc9b2	-0.681806447	1.49E+01
Cfap20	0.702278143	1.48E+01
Zfr	0.642947122	1.48E+01
Gprc5b	0.642982481	1.48E+01
Slc6a20a	-0.315407172	1.48E+01
Arf4	-0.535501667	1.47E+01
Lrrc6	0.534432798	1.47E+01
Cfap157	0.48223064	1.47E+01
Pla2g5	-0.392317041	1.47E+01
Vit	0.558494524	1.46E+01
Armc4	0.52620823	1.45E+01
Sash1	0.54422117	1.45E+01
Ppp1r3b	-0.293181763	1.45E+01
Plin5	0.573207975	1.45E+01
Aig1	0.673479965	1.45E+01
Slc22a8	-0.301149923	1.45E+01

Dock5	-0.392965169	1.45E+01
Cfap43	0.590652769	1.45E+01
Six1	-0.424923791	1.44E+01
Gm44109	0.565778938	1.44E+01
Cldn2	-0.451331005	1.44E+01
Trim37	0.584208744	1.44E+01
Pcp4	-1.00384041	1.44E+01
Mdfic	-0.375728558	1.43E+01
Phkg1	0.561573752	1.43E+01
Kcnj16	0.489200655	1.43E+01
Car9	0.523069209	1.43E+01
Mest	-0.477118954	1.43E+01
Dnah9	0.65950212	1.43E+01
Cpxm2	-0.408193193	1.43E+01
Chchd6	0.680385994	1.42E+01
Nr4a2	-1.069577906	1.42E+01
Ldb2	0.448193795	1.42E+01
Pou3f2	0.503722845	1.42E+01
Glmp	-0.431939436	1.42E+01
Zp3	0.56429918	1.41E+01
Adamts2	-0.590733671	1.41E+01
Cd74	-2.201500641	1.41E+01
Ift22	0.711740776	1.41E+01
Htra3	-1.0156058	1.40E+01
Gstm2	-0.479822888	1.40E+01
Slco1c1	-0.808703564	1.40E+01
Oscp1	0.608187065	1.40E+01
Hacd4	-0.589512504	1.40E+01
Slc16a2	-0.651875656	1.40E+01
Apc	0.571086994	1.39E+01
Aldoa	-0.612403117	1.39E+01
Gm17750	0.589130333	1.39E+01
Fkbp5	-0.396333711	1.39E+01
Eif5a	-0.574528762	1.38E+01
Eif5	0.525429789	1.38E+01
Unc119	-0.526218259	1.38E+01
Nat9	0.550047975	1.38E+01
Acot5	0.546778339	1.38E+01
Eif4a1	-0.511555886	1.38E+01
Lix1	0.75753693	1.37E+01
1700029J07Rik	0.623063264	1.37E+01
Rgcc	0.815647958	1.37E+01
Tmem238	-0.351851737	1.37E+01
H2-T22	-0.316172067	1.37E+01
Etfa	0.656871296	1.37E+01
Hsph1	0.692511268	1.37E+01
Afap1l1	-0.561706019	1.37E+01

Uchl1	0.520164351	1.37E+01
Fsip1	0.478712564	1.36E+01
Efhb	0.488539943	1.36E+01
Asrgl1	0.960759743	1.36E+01
Bmp5	-0.45078493	1.36E+01
F5	-0.825709186	1.36E+01
Marcks	0.572964377	1.36E+01
Crip2	0.612634575	1.36E+01
Exoc1	0.619555558	1.35E+01
Gdf10	-0.700196739	1.35E+01
Nmb	0.464138108	1.35E+01
Fam81b	0.713068104	1.35E+01
Lgals3bp	-0.530440438	1.35E+01
Cetn4	0.777552312	1.34E+01
Anxa7	-0.443016853	1.34E+01
Cetn3	0.64133831	1.34E+01
Crispld1	-0.476393063	1.34E+01
Pdgfd	-0.292356713	1.34E+01
Gdpd5	-0.517654892	1.34E+01
H2-Eb1	-0.862717035	1.34E+01
Tomm20	-0.477275819	1.33E+01
Rspo3	-1.370809822	1.33E+01
Aldh2	-0.631657292	1.33E+01
Ttc16	0.453367512	1.33E+01
Nek1	0.570642837	1.33E+01
Sema3b	-0.365481633	1.33E+01
Pou3f3	0.58324297	1.33E+01
Atp6v1a	-0.570512861	1.32E+01
Atp5g1	-1.455464589	1.32E+01
Gm26740	-0.437008647	1.32E+01
Lipa	-0.360658753	1.32E+01
Npas3	0.614778097	1.32E+01
Spef1	0.632876148	1.31E+01
Hras	-0.535762935	1.31E+01
1700007K13Rik	0.666906285	1.31E+01
Nme5	0.665963255	1.31E+01
Tspan6	0.587047816	1.31E+01
Arhgef26	0.544262952	1.31E+01
Clu	0.571513755	1.31E+01
1500009C09Rik	0.526843277	1.30E+01
Dst	0.74458738	1.30E+01
Sostdc1	-1.479966056	1.30E+01
Kcnk5	-0.653070613	1.30E+01
Itgbl1	-0.383520209	1.30E+01
Ywhae	0.430363043	1.29E+01
Map3k19	0.430642779	1.29E+01
Rilp	-0.264242408	1.29E+01

Ifi27l2a	-0.498125169	1.29E+01
Dnah10	0.470018563	1.28E+01
Ttll6	0.399788047	1.28E+01
Serping1	-0.516970032	1.27E+01
Efcab2	0.592265712	1.27E+01
Tspo	-0.624756202	1.27E+01
Fzd7	-0.390770658	1.26E+01
Dync2li1	0.634742524	1.26E+01
1700040L02Rik	0.742057877	1.26E+01
Nid1	-0.374520811	1.26E+01
Cetn2	0.698111944	1.26E+01
Dhrs7	0.669215808	1.26E+01
Clmp	-0.41847012	1.26E+01
Fmn2	0.465133523	1.26E+01
Thsd4	-0.584388416	1.25E+01
Got2	-0.428143411	1.25E+01
Mrpl20	-0.507875543	1.25E+01
Psap	-0.754152575	1.25E+01
Ccdc108	0.609663013	1.25E+01
Gnai2	0.533908579	1.24E+01
Mapk15	0.52142983	1.24E+01
Gpr4	-0.282517585	1.24E+01
Ttc12	0.57839996	1.24E+01
Maats1	0.516534318	1.24E+01
Dnah11	0.58245266	1.24E+01
Ltbp4	-0.569886601	1.24E+01
Ube2m	-0.456613041	1.24E+01
Pbx3	-0.421205248	1.23E+01
Fank1	0.556021906	1.23E+01
Tango6	0.543661733	1.23E+01
Scn7a	0.52244892	1.23E+01
Prg4	-1.639510864	1.22E+01
Gaa	-0.530198171	1.22E+01
Syt10	0.439594495	1.22E+01
Ptp4a3	-0.332933423	1.22E+01
1700037C18Rik	0.516747173	1.21E+01
Dsg2	0.532836484	1.21E+01
Fuca1	-0.557973338	1.21E+01
Nr2f2	-0.516356045	1.21E+01
Myo16	0.49443611	1.21E+01
Pfn1	-0.504268458	1.21E+01
Slco4a1	-0.37965618	1.20E+01
Dbp	0.685916079	1.20E+01
Polr2l	-0.392360107	1.20E+01
Gm1673	0.594279417	1.20E+01
Gm12840	-0.905501432	1.20E+01
Abhd14a	0.596490485	1.20E+01

Thumpd3	-0.596796085	1.20E+01
Speg	-0.310064635	1.19E+01
Spag8	0.483619356	1.19E+01
Id3	-0.798588198	1.19E+01
Ptges	-0.433763108	1.19E+01
Ankrd66	0.469144062	1.19E+01
2610301B20Rik	0.567697174	1.19E+01
Defb11	-0.321052557	1.19E+01
Prrg4	0.502743786	1.19E+01
Rpl39	-0.531232304	1.18E+01
Dpysl2	0.453887922	1.18E+01
Slc26a2	-1.741480716	1.18E+01
Acot1	0.657091981	1.18E+01
Ccdc96	0.549157133	1.18E+01
Anxa8	0.555813569	1.18E+01
Tekt1	0.637722908	1.18E+01
Tmem160	-0.437465644	1.18E+01
0610009L18Rik	0.589646184	1.18E+01
Spire1	0.495107924	1.18E+01
Rpl10	-0.533151872	1.17E+01
Parva	-0.429451987	1.17E+01
Tmsb10	-0.793656998	1.17E+01
Bcam	-0.384351509	1.17E+01
Copz2	-0.372968479	1.17E+01
H2afv	0.522875941	1.17E+01
Ide	-0.375908536	1.17E+01
Crocc	0.533457982	1.17E+01
mt-Nd4l	0.623323824	1.17E+01
Tsc22d4	0.73657345	1.17E+01
Mcfd2	-0.409261432	1.17E+01
Ociad2	-0.812165333	1.17E+01
Acot6	0.441352722	1.16E+01
Abhd2	-1.005831944	1.16E+01
Wdr6	-0.483030677	1.16E+01
Gpx1	-0.626805473	1.16E+01
Dcaf12l1	-0.327222335	1.16E+01
Degs1	-0.517784532	1.16E+01
Ins2	-0.256720606	1.16E+01
Ccdc65	0.495182933	1.16E+01
Rab20	-0.275753899	1.16E+01
Atp1b2	0.788809988	1.16E+01
Ndufa3	-0.585781011	1.16E+01
Dnaic2	0.559283348	1.16E+01
Gnal	0.473853565	1.15E+01
Mbd3	-0.460505852	1.15E+01
Ccdc40	0.427246058	1.15E+01
Slc15a2	0.54900929	1.15E+01

Col9a3	-1.211630536	1.14E+01
Psat1	0.510754853	1.14E+01
Calm2	0.55655867	1.14E+01
Kif3a	0.569605938	1.14E+01
Cdc14a	0.529488916	1.14E+01
Igfbp4	-0.957318544	1.14E+01
Tbx15	-0.314741773	1.13E+01
Dse	-0.443770791	1.13E+01
Txndc17	-0.427719472	1.13E+01
Ift81	0.613219847	1.13E+01
Smkr-ps	0.487829988	1.13E+01
Eva1a	0.473054986	1.13E+01
Fbxo33	-0.321600987	1.13E+01
Slc4a2	-0.650071125	1.13E+01
Nhsl2	-0.36440288	1.13E+01
1700028P14Rik	0.525220519	1.12E+01
Supt20	0.556928632	1.12E+01
Sntb2	-0.25938983	1.12E+01
Ppm1h	-0.391405672	1.12E+01
Rtn4	0.499561578	1.12E+01
Cdh2	0.49329277	1.12E+01
Wrn	0.575839236	1.12E+01
Tes	-0.291081869	1.12E+01
Dap	-0.371626862	1.12E+01
Ccdc88a	0.612918132	1.12E+01
Sncg	0.884725636	1.11E+01
Tmeff2	-0.799014142	1.11E+01
Nin	0.642324136	1.11E+01
Dynlt3	-0.664100266	1.11E+01
Cd59a	-0.580751181	1.11E+01
Dnaja4	0.637321537	1.11E+01
Adgra1	0.377429005	1.11E+01
1110008P14Rik	-0.331822029	1.10E+01
Srsf2	-0.481919577	1.10E+01
Zfp704	0.511694806	1.10E+01
Alpl	-0.966021067	1.10E+01
Mrpl54	-0.340785081	1.10E+01
Oard1	0.648038587	1.10E+01
Msx1	-0.685449249	1.10E+01
Hes6	-0.394005716	1.10E+01
Mknk2	-0.345514682	1.10E+01
Cfap69	0.542964696	1.10E+01
Ccdc78	0.443307785	1.10E+01
Hmgn1	0.588022812	1.09E+01
Gfap	1.247156256	1.09E+01
Baiap2	0.479587303	1.09E+01
Klf2	-1.752141655	1.09E+01

Grb10	-0.364075903	1.09E+01
Taco1os	0.476901968	1.09E+01
Tnfrsf11b	-0.560731888	1.08E+01
Fibin	-0.550003023	1.08E+01
Lrriq1	0.588175494	1.08E+01
Cspp1	0.547028728	1.08E+01
St8sia1	0.448479081	1.08E+01
Hnrnpf	0.552232456	1.08E+01
Pmepa1	-0.715408197	1.08E+01
Oca2	-0.266330774	1.07E+01
Spag16	0.482909764	1.07E+01
Prdx4	0.545772022	1.07E+01
Ubl3	0.550543944	1.06E+01
Uqcrb	-0.707389125	1.06E+01
RP24-492L15.6	0.391487938	1.06E+01
Epas1	-0.390581674	1.06E+01
Strbp	0.593743689	1.06E+01
Ltbp3	-0.527732277	1.06E+01
Lrrc71	0.426893818	1.06E+01
Clec2d	-0.624319161	1.06E+01
Bche	-1.165772288	1.05E+01
Gm7173	0.436329799	1.05E+01
Dnajb2	0.621581148	1.05E+01
1810010H24Rik	0.485601577	1.05E+01
Zbbx	0.383268604	1.05E+01
Col25a1	-0.751399543	1.04E+01
Shisa3	-0.326609325	1.04E+01
Erich3	0.397188001	1.04E+01
Fdx1	-0.521836401	1.04E+01
Ndufa4	-1.021329048	1.03E+01
Slc22a23	0.43936415	1.03E+01
Mapk3	-0.687875493	1.03E+01
Sgce	0.449073369	1.03E+01
Ccdc3	0.544560968	1.03E+01
Polr2m	-0.53250919	1.03E+01
Bst2	-0.70038127	1.03E+01
Eps8	0.540378671	1.03E+01
Pcdh7	-0.573161427	1.02E+01
Caly	0.624060885	1.02E+01
BC026585	0.559042913	1.02E+01
Zmynd10	0.472787812	1.02E+01
Sema3d	-0.326942554	1.02E+01
Chchd2	0.376900216	1.02E+01
Rsph14	0.372225168	1.02E+01
Lrp1	-0.717713922	1.01E+01
Lca5	0.50878508	1.01E+01
Pcsk5	-0.331950186	1.01E+01

Cxcl12	-0.746225979	1.01E+01
Nbl1	-1.529032962	1.01E+01
Gnao1	0.613271355	1.01E+01
Dcn	-0.810546249	1.01E+01
Smim4	-0.375459195	1.01E+01
Asgr1	-0.357997634	1.00E+01
Lpar1	0.464161504	1.00E+01
St3gal6	0.605973798	1.00E+01
Dut	-0.325348325	1.00E+01
Tspan8	-0.556316173	1.00E+01
Col3a1	-0.481858965	9.98E+00
Tmem55a	-0.286965412	9.95E+00
Sod2	0.527876274	9.93E+00
Calr	-0.564428635	9.91E+00
Atp6v1e1	-0.583733484	9.90E+00
Pcm1	0.579278788	9.90E+00
Atp6v0b	-0.432745944	9.89E+00
Dnal1	0.448616697	9.85E+00
Sdf2l1	-0.451093618	9.84E+00
Wdr78	0.529488736	9.83E+00
Ropn1l	0.448292433	9.83E+00
Srek1ip1	0.571294279	9.82E+00
Musk	0.435848108	9.82E+00
Gulp1	-0.325357737	9.79E+00
Gjb2	-0.697677284	9.77E+00
Sorl1	0.525834523	9.72E+00
Cdh1	-0.509035635	9.71E+00
Dnaic1	0.501679984	9.70E+00
Hipk1	0.713463959	9.68E+00
Nfe2l2	-0.667495993	9.66E+00
Notch2	-0.53050743	9.66E+00
Scarf2	-0.332709046	9.65E+00
Rer1	-0.36943825	9.65E+00
Serpinf1	-0.691948794	9.65E+00
Ggh	-0.513111257	9.65E+00
Tctex1d1	0.386442155	9.59E+00
Il10rb	-0.337708111	9.58E+00
Ralgps2	0.416541999	9.58E+00
Cfap46	0.487615537	9.55E+00
Cdk5rap2	0.368495116	9.54E+00
Tle1	-0.317039695	9.53E+00
Rpgr	0.479690612	9.51E+00
Fsd1l	0.487250192	9.48E+00
Timm8b	-0.423658058	9.47E+00
B2m	-0.620359405	9.47E+00
1700013F07Rik	0.419800976	9.45E+00
Ndufs8	-0.513379979	9.45E+00

Epn3	-0.345592945	9.43E+00
Slc6a1	0.607703176	9.42E+00
Zbtb20	0.642516293	9.42E+00
Rpap3	0.455019064	9.41E+00
Rprm	0.436313922	9.41E+00
Ndufv3	-0.731322814	9.41E+00
H13	-0.385293749	9.40E+00
Rcn3	-0.549042913	9.40E+00
Dnajc7	0.657195114	9.40E+00
Dcdc2a	0.538643591	9.39E+00
Ak9	0.4656925	9.39E+00
Ier3	-0.378954117	9.39E+00
Hspb8	-0.358917504	9.38E+00
Psmc3ip	0.509830965	9.37E+00
Slc25a4	-0.377172011	9.37E+00
Glb1	-0.344908673	9.36E+00
Otx2	-0.62352329	9.36E+00
Ahsa1	0.637068687	9.35E+00
Ribc2	0.338016941	9.34E+00
Sf3b2	0.494020167	9.34E+00
Cfap57	0.433320313	9.34E+00
Syt11	0.682269839	9.34E+00
Rsph9	0.588804439	9.33E+00
Fzd3	0.441908602	9.33E+00
Hspb1	-0.576741577	9.32E+00
Dzank1	0.439569231	9.32E+00
Gm29508	0.431492629	9.31E+00
Fbln7	0.497337841	9.30E+00
Ctsa	-0.410267355	9.29E+00
Nr2f1	0.653123257	9.26E+00
Ifi35	-0.274683084	9.23E+00
Trp53i11	-0.52669155	9.22E+00
Traf1	0.442186002	9.21E+00
Cab39l	-0.873496125	9.20E+00
Polr1d	-0.449365502	9.19E+00
Ptgdr	-0.283559396	9.18E+00
Wdr31	0.411845023	9.13E+00
Gm1661	0.397055505	9.12E+00
RP23-435E16.1	0.361612988	9.11E+00
Gxylt2	-0.344290082	9.11E+00
Plcd4	0.474830488	9.11E+00
Nt5e	-0.281647747	9.10E+00
Ttll9	0.401190413	9.08E+00
Penk	-0.751166693	9.05E+00
Saxo2	0.463962299	9.04E+00
Lekr1	0.435845134	9.03E+00
Tcf4	0.606323212	9.02E+00

Dnah1	0.422793357	9.02E+00
Rab3ip	-0.363811992	9.02E+00
Ddr1	0.641029183	9.01E+00
Lrrc46	0.438641959	9.00E+00
Hmgb2	0.537828162	9.00E+00
Uckl1os	0.435578337	8.99E+00
Cep290	0.605456675	8.98E+00
Atp5d	-0.660835245	8.97E+00
Shc1	-0.413660302	8.95E+00
Rorb	0.779880561	8.93E+00
Gpi1	-0.508416319	8.90E+00
Isyna1	-0.40323291	8.88E+00
Slc35a1	-0.31145758	8.87E+00
Ppia	-0.550379748	8.87E+00
Ncan	0.464000414	8.87E+00
H2-DMa	-0.358392666	8.86E+00
Trps1	0.517827314	8.84E+00
Hdlbp	-0.444038593	8.83E+00
Cpn1	0.510944764	8.83E+00
Usp50	-0.336876642	8.82E+00
Nxn	-0.431644057	8.82E+00
Mycbpap	0.453356494	8.81E+00
Zfp36l1	0.481403374	8.80E+00
Tex9	0.437890284	8.80E+00
Sod3	-0.348124534	8.78E+00
Slc22a17	-1.254688772	8.78E+00
Hspa1b	-0.41172969	8.76E+00
Unc13c	0.331138696	8.75E+00
2700033N17Rik	0.26628807	8.75E+00
Snx2	-0.333273066	8.75E+00
Gm10138	0.270670218	8.72E+00
Smim22	-0.523975275	8.71E+00
Sema6d	0.47285802	8.70E+00
Hmgcs2	-0.338143507	8.70E+00
Tagln2	0.573693367	8.67E+00
Wfdc1	-0.30621963	8.66E+00
4930402H24Rik	0.454587603	8.64E+00
Sars	-0.341446158	8.64E+00
Sirt2	0.475966408	8.63E+00
Fam149a	0.420810399	8.62E+00
Cers2	-0.363578576	8.62E+00
Hs3st1	-0.69751013	8.62E+00
Cfap61	0.404763804	8.61E+00
Synpo2	0.369292977	8.57E+00
Cmpk1	-0.303598599	8.55E+00
Ppp2r2b	0.443364048	8.55E+00
Naa38	-0.366447123	8.54E+00

Hbb-bs	-3.128204812	8.54E+00
Fam179a	0.258199851	8.54E+00
Pgf	-0.527820194	8.53E+00
Srgap3	0.357298359	8.50E+00
Fstl1	-0.801241026	8.49E+00
Pycard	-0.270787164	8.49E+00
Ubxn10	0.416525392	8.48E+00
Ndufa5	-0.346612981	8.45E+00
1500015O10Rik	-0.752062149	8.45E+00
Serpinb6a	-0.555506414	8.45E+00
Pantr1	0.802961605	8.41E+00
Arhgap18	0.49329513	8.41E+00
1700003E16Rik	0.377582511	8.39E+00
Glod4	0.509347401	8.39E+00
2210013O21Rik	-0.409856956	8.38E+00
Bbof1	0.358766635	8.38E+00
Oxct1	0.459533046	8.37E+00
Stpg1	0.393257588	8.37E+00
Dnah7a	0.371566859	8.36E+00
Trappc6b	-0.417701439	8.35E+00
Dnajb13	0.43203942	8.35E+00
Cpd	0.531866804	8.35E+00
Pcdh10	0.456678766	8.33E+00
Ankrd44	0.387710335	8.33E+00
Tceal3	0.495083294	8.33E+00
Plet1	0.389765412	8.32E+00
Ap2m1	-0.420543315	8.32E+00
Ap3s1	-0.318079954	8.29E+00
Lrrc43	0.365586411	8.28E+00
Cdkl2	0.442354067	8.27E+00
Cfap53	0.505735248	8.27E+00
Raly	-0.358731593	8.27E+00
Krt15	0.787083842	8.26E+00
Eno4	0.438790119	8.26E+00
Atp5j2	-0.609197597	8.24E+00
Ctsb	-0.44904683	8.23E+00
Tmsb4x	0.381543586	8.22E+00
Pax6	0.394766943	8.21E+00
Rabl2	0.423163801	8.20E+00
Ntsr2	0.96619698	8.18E+00
Rps12	-0.513103462	8.17E+00
Vwa3a	0.458440313	8.17E+00
Dusp14	0.525969376	8.16E+00
Ccdc151	0.305408746	8.15E+00
Akap6	0.377435554	8.14E+00
Hsbp1	0.430433531	8.14E+00
Wdr93	0.36877416	8.13E+00

Psme2	-0.45350312	8.12E+00
Pdlim7	-0.339580737	8.11E+00
Mpped2	-0.455495561	8.09E+00
Smim5	0.581313738	8.09E+00
Loxl1	-0.255437267	8.07E+00
Ufc1	-0.344672582	8.06E+00
Setd3	-0.337223797	8.06E+00
Slc6a11	0.728707547	8.06E+00
Abca9	-0.280920583	8.05E+00
Sybu	0.340966115	8.04E+00
Vpreb3	0.346125568	8.02E+00
Odc1	0.484122797	7.98E+00
Klf4	-0.931846183	7.98E+00
Ahnak2	-0.305231162	7.97E+00
Wdr49	0.251985516	7.96E+00
Zfos1	-0.332298108	7.95E+00
Cgnl1	-0.387992545	7.95E+00
Lsm14b	0.464283801	7.93E+00
Itih2	-0.414915664	7.91E+00
Tspan2	0.342627574	7.89E+00
Ift20	0.483740148	7.88E+00
Ttc29	0.340206393	7.88E+00
Sgk1	-0.583710807	7.87E+00
Ddit4l	0.439421335	7.87E+00
Endog	0.453145896	7.87E+00
Spata33	0.439520347	7.86E+00
Igfbp2	-1.501620413	7.86E+00
4430402I18Rik	0.417285593	7.84E+00
Dnaaf1	0.361751714	7.84E+00
Smim1	-0.427497315	7.83E+00
Arvcf	0.409083958	7.83E+00
Cpt2	-0.270378223	7.83E+00
Lrrc34	0.414039175	7.81E+00
4930547M16Rik	0.399905513	7.80E+00
Kif6	0.308576842	7.75E+00
Gnai1	-0.277241981	7.71E+00
Layn	0.413182224	7.70E+00
Cdipt	-0.312753511	7.70E+00
Gm12144	0.363235622	7.69E+00
Tns2	-0.318508164	7.68E+00
Egr1	-0.74622216	7.66E+00
Adcy5	-0.319254394	7.65E+00
Slc20a2	-0.309698068	7.63E+00
Serpinb9	-0.695410663	7.63E+00
Arhgef4	0.326619157	7.62E+00
Cox20	-0.485977936	7.61E+00
Wsb2	-0.289527034	7.59E+00

Rnf138rt1	0.435041986	7.58E+00
Bcan	0.906239812	7.57E+00
Fas	0.367052252	7.56E+00
Hnrnpdl	0.444656103	7.56E+00
Klhl13	0.445307862	7.56E+00
Synm	0.563352521	7.54E+00
Ppp2r3a	0.418593274	7.54E+00
Wdr63	0.40825018	7.54E+00
Mgst1	0.555345291	7.53E+00
Ewsr1	0.513245086	7.53E+00
Tctex1d2	0.539938824	7.52E+00
Tceb2	-0.504366256	7.51E+00
mt-Nd1	-0.684098258	7.51E+00
Sox6	0.397539809	7.49E+00
Tprkb	0.611784656	7.47E+00
Rnase4	0.497289	7.47E+00
Kif19a	0.336037005	7.46E+00
Atxn1	0.486147715	7.44E+00
Slc35g3	0.321814094	7.41E+00
Vasn	-0.264516061	7.41E+00
Prkcd	-0.278012682	7.40E+00
Sssca1	0.498851114	7.40E+00
Alad	-0.327631207	7.38E+00
Ccdc189	0.501142657	7.36E+00
Glis2	-0.263512342	7.36E+00
Fam184a	0.381342629	7.33E+00
Ang	0.435561174	7.33E+00
Arhgap29	-0.293789497	7.33E+00
Ttc30b	0.472482312	7.32E+00
Slit2	-0.527205177	7.32E+00
Ucma	0.636225976	7.31E+00
Wdr60	0.473201572	7.28E+00
Tbx18	-0.265772035	7.28E+00
Adipor1	-0.329787637	7.28E+00
Gm20661	0.388275165	7.26E+00
Fosl2	-0.331404835	7.24E+00
Meg3	0.500911004	7.24E+00
Slc38a2	-2.367275629	7.23E+00
Lix1l	-0.266559283	7.22E+00
Atp6ap1	-0.418270064	7.22E+00
mt-Nd3	-0.570208596	7.20E+00
Gm33050	0.268679214	7.19E+00
Spata7	0.343377067	7.19E+00
Slc2a12	-0.50540623	7.19E+00
St3gal5	-0.406581721	7.14E+00
Tenm3	0.29499993	7.13E+00
Bola1	0.52715955	7.12E+00

Pdk3	-0.257581033	7.11E+00
Gm28694	0.375392779	7.08E+00
Ssbp4	0.508666838	7.08E+00
Atp2b4	0.295172759	7.07E+00
Larp1b	-0.300066437	7.07E+00
Borcs8	-0.286202496	7.06E+00
Apbb2	0.434426597	7.06E+00
Lamb2	-0.605668068	7.05E+00
Yipf4	-0.315080792	7.05E+00
Gm14964	0.510856039	7.05E+00
Arhgap20	-0.357460015	7.04E+00
D8Ertd82e	0.389826245	7.02E+00
Mrap	-0.266108376	7.01E+00
Fam26e	-0.297392731	7.00E+00
Lgals3	0.658369938	6.99E+00
Slc39a1	-0.388700885	6.99E+00
Arl2	-0.362060458	6.98E+00
Tapbp	-0.609031287	6.98E+00
Stox2	0.40153826	6.96E+00
2610028H24Rik	0.256575808	6.93E+00
Slc9a3r1	0.525219701	6.93E+00
Ctsl	-0.587777128	6.92E+00
Spag1	0.355966276	6.92E+00
Acsbg1	0.919883448	6.91E+00
Mccc1	-0.338590421	6.90E+00
Pih1h3b	0.266456244	6.89E+00
Ly6a	-0.316239269	6.87E+00
Cenpv	0.46776555	6.86E+00
Ftl1	-0.445692468	6.85E+00
Trf	-1.373112965	6.84E+00
Psip1	0.472943285	6.83E+00
Ptms	-0.609114946	6.83E+00
Iqck	0.414322291	6.82E+00
Slc38a1	0.594912009	6.80E+00
Cers5	-0.327026116	6.79E+00
Rps2	-0.355956091	6.78E+00
Pus7	0.356057171	6.78E+00
Ankrd45	0.451321673	6.77E+00
Ctxn3	-0.411875486	6.76E+00
Rad50	0.479354612	6.76E+00
Spp1	-1.258086177	6.76E+00
Uqcc2	-0.344550418	6.71E+00
Gpld1	0.491291533	6.69E+00
Zfp385a	-0.26931768	6.68E+00
Efcab12	0.384696167	6.68E+00
Atp5l	-0.723580089	6.68E+00
Uqcr10	-0.849728087	6.68E+00

Cluap1	0.408040573	6.66E+00
Pls3	-0.322149973	6.66E+00
Slc20a1	0.305154082	6.65E+00
Daw1	0.369462397	6.64E+00
Nampt	-0.367941514	6.63E+00
Tmem159	-0.282702246	6.61E+00
Rnf128	0.30143083	6.61E+00
Poln	0.293239963	6.60E+00
Nr1d1	0.564468971	6.59E+00
Arpc2	-0.380945857	6.56E+00
Clptm1	-0.333189468	6.55E+00
Acbd7	0.326912388	6.54E+00
Pbx4	0.327288995	6.54E+00
Ogg1	0.398279991	6.52E+00
Rbp2	0.40540031	6.52E+00
Fgl2	-0.451512014	6.51E+00
Dnajc8	0.478114485	6.49E+00
6430531B16Rik	0.301649975	6.47E+00
Aga	-0.278665573	6.46E+00
Odf2l	0.399523805	6.46E+00
Gde1	-0.496374337	6.41E+00
Cct3	0.425165675	6.41E+00
Hrk	0.290972386	6.40E+00
Katnal2	0.394115168	6.40E+00
Cspg5	1.102513155	6.39E+00
Ctgf	-0.42959123	6.39E+00
Tpi1	-0.400882102	6.36E+00
Dsc3	0.314976486	6.36E+00
Cldn10	0.895148464	6.35E+00
Uqcrq	-0.751415806	6.35E+00
Uchl3	-0.268557826	6.34E+00
Tiam1	0.294370611	6.33E+00
B9d1	0.506858935	6.33E+00
Ebf1	-0.438177849	6.32E+00
Grn	-0.395625561	6.32E+00
Wdyhv1	0.438329806	6.30E+00
Tns1	-0.286931501	6.29E+00
Calr3	0.295849976	6.28E+00
Tgfb1i1	-0.385054643	6.28E+00
Rit2	0.402139117	6.28E+00
Fam181b	0.576061415	6.27E+00
Slc38a10	-0.362072111	6.27E+00
Csrp1	0.627030679	6.26E+00
Tsnaxip1	0.390156305	6.26E+00
Araf	-0.277014875	6.25E+00
Nucb1	-0.39062318	6.24E+00
Tmed1	-0.279681506	6.24E+00

Col6a5	0.60524207	6.22E+00
Tbata	0.303667435	6.22E+00
Mettl7a1	0.504757279	6.21E+00
Flrt2	-0.306311449	6.21E+00
Myo10	0.503721881	6.20E+00
Tnfrsf1a	-0.353666134	6.20E+00
Slc24a3	-0.304326672	6.19E+00
Ybx3	-0.47957106	6.19E+00
Kif9	0.418641818	6.18E+00
A830010M20Rik	0.291931609	6.18E+00
Cycs	-0.61511196	6.17E+00
Sall1	0.257968794	6.17E+00
Ndn	0.383743904	6.17E+00
Tbc1d31	0.333823433	6.16E+00
Dyrk3	0.353507283	6.15E+00
Ruvbl1	0.460936849	6.15E+00
Kazn	0.410018085	6.14E+00
Snrpg	0.515966584	6.14E+00
Pla2g12a	-0.392230609	6.13E+00
Tagln3	0.487076856	6.13E+00
Hexa	-0.443497612	6.11E+00
Lima1	-0.405950145	6.08E+00
Igsf11	0.290065131	6.07E+00
Slc13a4	-0.375122222	6.06E+00
Kif11	0.280867264	6.05E+00
Cldn5	-0.377528009	6.05E+00
Dlec1	0.373598926	6.04E+00
Palmd	-0.384982138	6.04E+00
Col8a2	-0.603569374	6.01E+00
Ppp1r1b	-1.015860093	6.00E+00
Lrguk	0.321788272	5.97E+00
Trim2	0.396577034	5.97E+00
Nat14	0.395923218	5.96E+00
Abcc5	0.345819163	5.95E+00
Vcam1	-0.45205987	5.95E+00
Cfap74	0.317664619	5.93E+00
Surf4	-0.310614039	5.92E+00
Spata18	0.330151483	5.92E+00
Creb5	0.320501891	5.91E+00
Cd109	0.301363469	5.90E+00
Prrt1	0.344435245	5.90E+00
Aebp1	0.575328177	5.89E+00
Ccdc60	0.382929842	5.87E+00
Calu	-0.321537898	5.86E+00
Cyp4v3	-0.398835524	5.86E+00
Abhd3	0.502974002	5.84E+00
Tgfbr3	-0.352786163	5.84E+00

Agpat4	0.437687525	5.82E+00
Oasl2	-0.475789793	5.82E+00
Ntm	0.616157225	5.82E+00
Faah	0.33840227	5.81E+00
Ndufs2	-0.368760005	5.80E+00
Wnk2	0.29151168	5.78E+00
Abcd3	-0.436368913	5.78E+00
Tril	0.482786679	5.77E+00
Naglu	-0.273560285	5.77E+00
Btg2	-1.015317164	5.76E+00
Atp6v0e	-0.37351929	5.76E+00
Cox6b2	-0.473732161	5.76E+00
Pet100	-0.303126321	5.76E+00
Kcnb2	-0.308947609	5.73E+00
Tceal8	-0.387924056	5.72E+00
Pcolce2	-0.531173845	5.71E+00
Hmgn5	0.460303736	5.70E+00
Arl6ip1	-0.817980658	5.70E+00
Hhatl	0.315265619	5.70E+00
Smad6	-0.25742824	5.69E+00
Blvrb	-0.335009174	5.69E+00
Pisd	0.359173328	5.69E+00
Slc50a1	-0.384023318	5.68E+00
Serp1	-0.346784371	5.68E+00
Emc2	-0.342599916	5.67E+00
Sorcs2	0.284593669	5.67E+00
Adhfe1	0.380658282	5.66E+00
Gm19412	0.369347114	5.65E+00
Dchs1	0.296034284	5.64E+00
Uqcr11	-0.69315771	5.64E+00
Cryab	-0.263047766	5.63E+00
Zmynd12	0.406294893	5.63E+00
Snrpn	-0.358023597	5.63E+00
Nthl1	0.337585323	5.62E+00
Ccdc13	0.300694422	5.62E+00
C230037L18Rik	0.30403926	5.62E+00
Podn	-0.256986853	5.61E+00
Ube2b	0.468967802	5.60E+00
S1pr1	0.706878056	5.59E+00
Ribc1	0.375874155	5.58E+00
Rpl41	-0.32740235	5.57E+00
Ythdc1	0.45887044	5.57E+00
Actn4	-0.333902993	5.57E+00
Arhgap21	0.465744785	5.57E+00
Gm561	-0.255023315	5.56E+00
Crtc3	0.396503376	5.55E+00
Rps15a	-0.326755227	5.53E+00

Clcn4	0.48582058	5.53E+00
Egln2	-0.265095577	5.52E+00
Cfap221	0.256670495	5.51E+00
Spata6	0.37412297	5.50E+00
Cmtm5	0.531672302	5.48E+00
Rchy1	-0.357301334	5.48E+00
4-Mar	0.253324062	5.48E+00
Aar2	-0.259262039	5.48E+00
Agbl3	0.342425773	5.47E+00
Dnah7b	0.3474229	5.47E+00
Atp2b1	0.475171303	5.45E+00
Sap30bpos	0.340363533	5.45E+00
Atp6v1f	-0.390346889	5.45E+00
Kndc1	0.28808367	5.45E+00
Tmbim4	-0.365445581	5.44E+00
Timm17a	-0.412755088	5.44E+00
Eif4g2	-0.331858835	5.43E+00
H2-Ab1	-0.86868076	5.43E+00
Tax1bp3	-0.266373743	5.42E+00
D830044I16Rik	0.29468418	5.41E+00
Atxn7l1	0.381158899	5.41E+00
Lztfl1	0.427292485	5.41E+00
Jam3	0.367140592	5.40E+00
Psme1	-0.357942566	5.40E+00
Ctnnal1	-0.358200919	5.40E+00
Myl9	0.462808914	5.39E+00
Etl4	-0.352372678	5.39E+00
Ankrd42	0.328729367	5.39E+00
Fkbp9	-0.34893747	5.39E+00
Nap1l5	0.573536942	5.38E+00
Srpr	-0.418855729	5.38E+00
Reps2	-0.326982922	5.37E+00
Pik3r3	-0.274543959	5.35E+00
Cnbp	0.402889536	5.34E+00
Pdlim2	-0.289029964	5.33E+00
Lhx2	0.475106416	5.33E+00
Usp25	-0.303798297	5.31E+00
Cisd2	-0.3627967	5.31E+00
Imp3	-0.287662514	5.31E+00
Negr1	0.319690366	5.30E+00
Nrn1	-0.256928017	5.30E+00
Cacna1d	0.263118684	5.29E+00
E130114P18Rik	0.338373599	5.29E+00
Epn1	-0.269434444	5.29E+00
Tmed3	-0.377770935	5.27E+00
Fam229b	0.421089722	5.27E+00
Wdr19	0.354736151	5.27E+00

Soga3	0.260633015	5.27E+00
Hilpda	-0.502473542	5.26E+00
Hmbs	-0.285479685	5.24E+00
Nme7	0.327126726	5.23E+00
Ccdc181	0.400507989	5.22E+00
Arf2	-0.284540276	5.21E+00
Naaa	0.434512428	5.21E+00
Mfsd7b	0.32266715	5.19E+00
St8sia2	0.305357731	5.18E+00
Arhgap31	-0.321121618	5.18E+00
Higd1a	-0.404259789	5.18E+00
Atp5g3	-0.553880409	5.17E+00
Man2b1	-0.295284071	5.16E+00
Npnt	0.422054447	5.16E+00
Ctnnd2	0.443076868	5.15E+00
Slc12a7	-0.443121693	5.12E+00
Dab1	0.255962201	5.09E+00
Dpp7	-0.26755937	5.08E+00
Sar1a	-0.312592424	5.08E+00
Nrxn3	0.343273875	5.08E+00
Dtx1	0.338624203	5.07E+00
Tcp11	0.278231758	5.06E+00
Ppp1cb	0.395073186	5.04E+00
Eef2k	0.371382322	5.04E+00
Cep41	0.334715407	5.02E+00
Hey1	0.389819794	5.02E+00
Smpd1	-0.381005794	5.02E+00
Lca5l	0.278152541	5.02E+00
Ndufb11	-0.428069261	5.01E+00
Myl4	0.481595659	5.00E+00
Cfl2	-0.298926539	4.99E+00
Srl	0.297904669	4.98E+00
Ccdc103	0.307056423	4.98E+00
Rps11	-0.350336408	4.98E+00
Hmgn2	0.398654677	4.98E+00
Pdpn	-0.382844722	4.98E+00
Rbm24	0.285500898	4.98E+00
Tsn	0.377570581	4.97E+00
Phyhipl	0.679191125	4.96E+00
Dyx1c1	0.297659275	4.96E+00
Lsm4	-0.318572638	4.95E+00
Vtn	-0.526961751	4.95E+00
Eps15	0.445315692	4.95E+00
Spata17	0.278218651	4.94E+00
Dync2h1	0.416429645	4.93E+00
Slc1a4	0.308962478	4.93E+00
Nadk2	0.457652869	4.93E+00

Rpl15	-0.348693179	4.92E+00
Magi2	0.342912089	4.90E+00
Prelid1	-0.322043908	4.89E+00
Tmem216	0.43715246	4.87E+00
Ttc9	0.393863217	4.87E+00
Cltb	-0.32409721	4.86E+00
Cntrl	0.407478917	4.86E+00
Ppdpf	0.48170052	4.86E+00
2010107E04Rik	-0.649055031	4.86E+00
Asah1	-0.315274588	4.85E+00
Serpinb1a	0.3872214	4.85E+00
Phtf1	0.387552229	4.85E+00
Sat1	0.454132772	4.84E+00
Coq9	-0.260069783	4.84E+00
Poc5	0.282045447	4.83E+00
Nme1	-0.382839364	4.83E+00
Fkbp1a	-0.354871194	4.83E+00
2410089E03Rik	0.37364216	4.82E+00
Rassf2	-0.658311255	4.82E+00
Txnip	0.530252209	4.81E+00
Tmem232	0.282014314	4.81E+00
Nek7	-0.306463159	4.80E+00
1810037I17Rik	0.378406709	4.79E+00
Apoo	-0.514958831	4.79E+00
Csrp2	0.650148492	4.78E+00
Mtfr1	-0.26145719	4.78E+00
Lxn	0.576654677	4.78E+00
Nedd8	-0.365059921	4.74E+00
Tmem237	-0.508670505	4.73E+00
Nkapl	0.252341045	4.73E+00
Ppp6c	-0.271675758	4.73E+00
Sned1	-0.413261825	4.73E+00
Acaa2	-0.564770147	4.71E+00
Sirpa	0.472635927	4.71E+00
Gclm	0.419988216	4.70E+00
Dnajc6	0.331678895	4.70E+00
Rpl36al	-0.3567043	4.70E+00
Kctd12	-0.948630785	4.69E+00
Hist1h4i	0.477446399	4.69E+00
Elmod2	0.312305348	4.69E+00
Cox7a2	-0.552075977	4.69E+00
Tmem147	-0.376865773	4.68E+00
Fnbp1	0.378310688	4.68E+00
Rpl28	-0.320203518	4.67E+00
Stk33	0.353661954	4.66E+00
5-Mar	-0.295580636	4.66E+00
Rraga	-0.348441366	4.66E+00

Sdhd	-0.364111348	4.66E+00
Lrpap1	-0.412175745	4.64E+00
Ndufb5	0.385750488	4.63E+00
Rpl18a	-0.322633193	4.62E+00
Bcl9	0.295460273	4.62E+00
Gpx8	-0.376555451	4.62E+00
Sdpr	-0.374273434	4.62E+00
Sclt1	0.33868083	4.61E+00
Ednrb	0.447811907	4.59E+00
Ktn1	0.415368892	4.59E+00
Nupr1	-0.478308404	4.59E+00
Pfdn2	0.443491477	4.58E+00
Fbxo16	0.343988581	4.58E+00
Trp53bp2	0.51393685	4.58E+00
Hhip	-0.337399688	4.57E+00
Egfr	-0.298363675	4.57E+00
4931406C07Rik	0.486644767	4.56E+00
Ndrg1	-1.167244005	4.55E+00
Fkbp7	-0.266566024	4.54E+00
Neo1	-0.418647317	4.53E+00
Ndufa13	-0.379846402	4.53E+00
Ank	0.397512298	4.52E+00
4833427G06Rik	0.405435372	4.49E+00
Atp8a1	0.383374278	4.49E+00
P3h3	-0.260303504	4.48E+00
Adamts1	-0.561909822	4.47E+00
Mpp5	0.345924219	4.47E+00
Macrod1	0.428875593	4.47E+00
Sin3b	-0.30882979	4.45E+00
P4ha3	0.27392497	4.45E+00
Kif1b	0.44649213	4.45E+00
Lhfpl4	0.262995801	4.43E+00
Fam161b	0.30135996	4.43E+00
Fhod3	0.277090317	4.42E+00
Fus	0.354282243	4.42E+00
Znhit1	0.494911292	4.41E+00
Tmem138	0.306728593	4.40E+00
Aif1l	0.253467087	4.39E+00
Abcc4	-0.332217349	4.39E+00
Dalrd3	0.409392376	4.36E+00
Mb	0.293569984	4.36E+00
Shank2	0.341784627	4.35E+00
Prex2	0.629856317	4.35E+00
Cox7a1	-0.377714471	4.34E+00
Sfxn5	0.626796761	4.32E+00
Metrn	0.361734523	4.31E+00
Osbpl6	0.302942321	4.31E+00

Ddt	0.476505935	4.31E+00
Cd151	-0.490881395	4.30E+00
Slc25a39	-0.347788075	4.28E+00
Ccdc167	-0.265022412	4.28E+00
Tgif1	0.3745567	4.28E+00
Rpgrip1l	0.369522664	4.26E+00
Rbpms	0.339547381	4.26E+00
Mrps16	-0.256735366	4.25E+00
Spsb1	0.265309397	4.23E+00
Rfk	-1.040153657	4.23E+00
Fbxo36	0.551290697	4.22E+00
Car2	-0.635907265	4.22E+00
Mospd1	-0.406279168	4.21E+00
Elovl7	-0.530005035	4.21E+00
Fam13a	0.343963892	4.21E+00
Chaf1a	0.28294755	4.20E+00
Elovl1	-0.265668812	4.20E+00
4732414G09Rik	0.264903328	4.19E+00
Dtna	0.432143739	4.19E+00
Itm2c	0.313415619	4.16E+00
Hacd2	0.449892131	4.15E+00
Fam175b	0.414926596	4.13E+00
Kif27	0.317274635	4.13E+00
Rab21	-0.286877227	4.13E+00
Casd1	0.444781645	4.12E+00
Dnajc15	0.409203042	4.11E+00
Basp1	0.260273345	4.10E+00
Fmo5	0.281420781	4.10E+00
Lrrc73	0.33239246	4.10E+00
1-Mar	0.255350487	4.09E+00
Ppp1ca	-0.276920002	4.08E+00
Kcnj2	-0.298133336	4.07E+00
Mast4	0.32422439	4.07E+00
Nrxn1	0.792355593	4.06E+00
Shfm1	0.336555734	4.06E+00
Sfrp4	-0.897450287	4.05E+00
Clta	-0.313327138	4.05E+00
Stt3a	-0.331954942	4.04E+00
Cyp4f15	0.268666372	4.01E+00
RP24-185P7.1	0.257295066	4.01E+00
Tpt1	-0.29135776	4.01E+00
Cox6a1	-0.419849636	4.01E+00
Ino80b	0.393879415	4.00E+00
Dynll1	0.417732096	4.00E+00
Ces1d	0.310495133	3.99E+00
Romo1	-0.37643904	3.99E+00
1110004E09Rik	0.428947219	3.98E+00

Col5a3	0.308978835	3.98E+00
Ift74	0.497051595	3.97E+00
Luzp2	0.981835348	3.96E+00
Srebf1	0.339434262	3.95E+00
Ccdc162	0.299077431	3.94E+00
Cdh11	-0.819719064	3.94E+00
Zc3h6	0.308557199	3.94E+00
Ndufa11	-0.657354679	3.93E+00
Tmem9	-0.270789559	3.92E+00
Ckap4	-0.470851532	3.92E+00
Pink1	0.395570965	3.91E+00
Prkag2	-0.344164942	3.90E+00
Tmem176b	-0.438644714	3.90E+00
Mthfd2l	0.453273997	3.90E+00
Ifnar2	-0.307254233	3.88E+00
Psmd8	-0.286788397	3.87E+00
Rpl19	-0.334299267	3.87E+00
Sorbs2	0.329868607	3.87E+00
Ergic2	-0.300828387	3.87E+00
Jam2	0.391245189	3.87E+00
Bmp2k	0.346034567	3.86E+00
Tmem176a	-0.446056003	3.85E+00
Col4a3bp	-0.279356094	3.85E+00
Chd5	0.304038224	3.85E+00
Itih3	0.416873073	3.83E+00
Steap2	-0.313372638	3.83E+00
Naa35	0.344788362	3.81E+00
Cnksr2	0.29434797	3.81E+00
Slc6a6	-0.541356582	3.81E+00
Ap3m1	0.45952593	3.80E+00
Ulk4	0.323047365	3.78E+00
Nt5c	0.43605223	3.78E+00
Faim2	0.257984795	3.77E+00
Cdc42bpa	0.45941273	3.73E+00
Nfe2l1	-0.383625788	3.73E+00
Sh3bgrl3	-0.290763132	3.72E+00
Cdc42	-0.302169618	3.71E+00
Snap47	-0.305644839	3.70E+00
Tmem229a	0.349486991	3.70E+00
St3gal4	-0.322060777	3.68E+00
Cdkl4	0.284830748	3.67E+00
Mtx2	-0.308503562	3.64E+00
Rpl36	-0.296091132	3.64E+00
Kifap3	0.382343441	3.63E+00
Akt1	-0.288347833	3.62E+00
Actr3	-0.335078866	3.60E+00
Arl13b	0.316233991	3.60E+00

Atp6v0e2	0.315943698	3.58E+00
Gjb6	-0.7388165	3.58E+00
Mapk4	-0.260175232	3.58E+00
Zfand4	0.296003206	3.58E+00
Rplp2	0.314278432	3.56E+00
Myl12a	-0.344128119	3.56E+00
Dusp18	0.362682421	3.55E+00
Plekha2	0.362078757	3.54E+00
Sh3glb2	-0.357221992	3.52E+00
Gas8	0.367403929	3.51E+00
Vwa5b1	0.273918	3.51E+00
Pkm	-0.37092385	3.51E+00
Adprh	0.381685469	3.49E+00
Ophn1	0.331241641	3.49E+00
Fbn1	-0.299225556	3.48E+00
Syf2	0.44117159	3.48E+00
Nudt11	0.255721613	3.47E+00
Trpc7	0.302726402	3.47E+00
Ctsz	-0.333477909	3.47E+00
Cdk14	0.26409786	3.46E+00
Gt(ROSA)26Sor	-0.270707973	3.46E+00
Fth1	0.330683811	3.46E+00
Steap1	-0.560651213	3.46E+00
Cml1	0.497549666	3.46E+00
Scara3	-0.457028244	3.45E+00
Adam22	0.288123857	3.44E+00
Kif21a	0.304290198	3.42E+00
Zc2hc1a	0.427197374	3.42E+00
Med31	0.366701838	3.41E+00
Lims1	-0.319459182	3.41E+00
Sp3os	0.420668564	3.39E+00
Gda	0.266360327	3.38E+00
Vdac1	-0.365817919	3.38E+00
Ssr1	-0.347948571	3.38E+00
2010300C02Rik	0.278731258	3.37E+00
Arhgdia	-0.303204625	3.37E+00
Ssb	0.349909019	3.36E+00
Tst	0.716101466	3.36E+00
Dek	0.308914054	3.36E+00
Tcte1	0.255530962	3.35E+00
Smad5	0.360742089	3.35E+00
Cyfip2	-0.265184211	3.34E+00
2810428I15Rik	-0.319450036	3.34E+00
Casq2	0.251079753	3.34E+00
Hp	0.314930242	3.33E+00
Tmem67	0.314009774	3.32E+00
Kctd1	0.319898664	3.30E+00

Ap3m2	0.360447779	3.29E+00
Mbtps1	-0.376770753	3.29E+00
Gm20383	0.275069216	3.28E+00
Bbs7	0.304646168	3.27E+00
Sra1	-0.27117112	3.27E+00
1110032A03Rik	0.454134946	3.27E+00
Akip1	0.328541505	3.26E+00
Rpl38	0.287677447	3.26E+00
Zic4	-0.320542487	3.23E+00
Elmod3	0.34225704	3.20E+00
Srp9	-0.30832978	3.20E+00
Lman2l	-0.352999896	3.19E+00
Odf2	0.305137147	3.19E+00
Etfb	-0.484108846	3.19E+00
Zrsr2	-0.255948795	3.18E+00
Map2k2	-0.298556491	3.18E+00
Utp14b	0.386845657	3.17E+00
Xpc	0.310628498	3.17E+00
Cep128	0.262936074	3.16E+00
Fam47e	0.40099199	3.13E+00
Kcnn2	0.26009407	3.13E+00
Tmie	0.281037034	3.13E+00
Cst3	0.626017776	3.11E+00
Phb	-0.253985236	3.10E+00
Arpc5	-0.288560618	3.10E+00
Acadsb	-0.298572338	3.09E+00
Ssx2ip	0.338737745	3.08E+00
Lrwd1	0.315376736	3.07E+00
Usp24	0.279858313	3.06E+00
Hspd1	0.342588116	3.06E+00
Hnrnpc	0.434109224	3.05E+00
Rfx3	0.327047521	3.04E+00
Mtfr1l	-0.276562985	3.03E+00
Iqcd	0.267316115	3.02E+00
Ddo	0.292178004	3.02E+00
Bad	-0.272322933	3.01E+00
Elf1	-0.274775798	2.99E+00
Hnrnpab	-0.295016787	2.98E+00
Slf1	0.319694627	2.97E+00
Cfap36	0.423863556	2.95E+00
Smoc1	-0.696265437	2.95E+00
Ifitm3	0.526841351	2.94E+00
Fam98b	0.334284104	2.91E+00
Ahi1	0.433057015	2.90E+00
Pxdc1	0.326535002	2.88E+00
Sfrp1	-0.270125588	2.88E+00
S100a1	-0.267495427	2.88E+00

Cox8a	-0.66063123	2.87E+00
Lpin1	0.267020792	2.86E+00
Osbpl9	-0.278777418	2.86E+00
Socs2	-0.271848308	2.84E+00
Slc22a4	0.280399285	2.83E+00
Pbxip1	0.462493748	2.82E+00
Kcnmb4	0.261399992	2.81E+00
Lman2	-0.314690567	2.81E+00
Rap1a	-0.292851908	2.80E+00
Cmtm6	-0.276338427	2.80E+00
Sdc4	0.34969903	2.78E+00
Clec1b	0.296375237	2.77E+00
Zfand5	0.389356289	2.77E+00
Itpkb	0.451606958	2.77E+00
Isg15	-0.286717463	2.76E+00
C3	0.299407878	2.75E+00
Kdelr1	-0.304771231	2.75E+00
Atp2a2	0.367272247	2.75E+00
Prkcsh	-0.26685084	2.73E+00
D8Ertd738e	-0.274770294	2.73E+00
Slc24a4	0.30868464	2.72E+00
Rgs12	0.257101588	2.72E+00
Mark1	-0.257212883	2.71E+00
Nrcam	0.2873003	2.71E+00
Ift80	0.35880656	2.70E+00
Arl3	0.326921325	2.69E+00
Fos	-1.250189068	2.69E+00
Fam92b	0.45595147	2.68E+00
Serpinh1	-0.582206123	2.68E+00
Gm16160	0.346881826	2.67E+00
Itgb1	-0.465309771	2.66E+00
Rhou	0.296103059	2.66E+00
Itgb5	-0.375064186	2.66E+00
Rps25	0.302338207	2.65E+00
Irak1bp1	0.420732429	2.65E+00
H1fx	0.350367024	2.65E+00
Lhfp	-0.295826932	2.65E+00
Dctn3	-0.313839561	2.64E+00
Cisd1	-0.270936426	2.63E+00
Myo6	0.509298592	2.63E+00
Rab36	0.268082085	2.63E+00
Mid1	0.251075366	2.63E+00
Rasgrf1	0.314581892	2.62E+00
Ddah1	0.667452267	2.62E+00
Gfod2	0.283287561	2.61E+00
Akap9	0.438126437	2.61E+00
Hist1h2be	0.391623868	2.61E+00

Tppp	0.260976164	2.60E+00
Pgd	-0.267253703	2.58E+00
Coa3	-0.265777468	2.58E+00
Fbln1	-1.003488982	2.58E+00
Prps2	-0.353241405	2.56E+00
Sidt2	-0.305363557	2.55E+00
Emsy	0.280119328	2.55E+00
Ptk7	0.316948245	2.54E+00
Kdsr	-0.262710686	2.53E+00
Gars	-0.280474211	2.52E+00
Pid1	-0.400673385	2.51E+00
Mrpl39	0.385879999	2.50E+00
Zeb2	0.309726019	2.50E+00
Edf1	-0.273520955	2.50E+00
Irx5	0.266849087	2.48E+00
Man1a	-0.350662837	2.45E+00
Wscd1	0.277479871	2.44E+00
Cenpb	-0.326225874	2.43E+00
Cdk2ap1	0.362794808	2.42E+00
Wdr35	0.392998226	2.41E+00
Cat	-0.2564486	2.41E+00
Nkap	0.321536284	2.41E+00
Spcs2	0.32942733	2.41E+00
Efhc1	0.337038686	2.40E+00
Prkcdbp	0.304305218	2.40E+00
Mrps25	-0.302775404	2.40E+00
Txn2	-0.256111986	2.39E+00
Map9	0.295111491	2.39E+00
Psma5	0.338639562	2.38E+00
Scrg1	0.591993637	2.36E+00
Lrrc18	0.251707276	2.34E+00
Appl2	0.412422277	2.34E+00
Aurkaip1	-0.298479684	2.32E+00
Dnajb11	-0.261297958	2.31E+00
Cndp2	-0.25509191	2.31E+00
Mphosph6	0.352624631	2.30E+00
Cald1	0.37581231	2.29E+00
Dnajb6	0.3398249	2.28E+00
Med19	0.338771815	2.27E+00
Atxn7l3b	0.346022257	2.27E+00
Scd1	-0.251186229	2.27E+00
Erp29	-0.264298321	2.24E+00
Zfp703	0.354860468	2.24E+00
Rnf145	-0.278157148	2.24E+00
D3Ertd751e	0.286320018	2.21E+00
Trp53inp2	-0.252043699	2.21E+00
Bzw1	-0.770952023	2.21E+00

Slc24a5	-0.274498864	2.20E+00
Ift57	0.394289118	2.19E+00
Ptrf	-0.56175096	2.19E+00
Ssr4	-0.308082352	2.18E+00
Dnaaf3	0.324117567	2.18E+00
Fam151b	0.287768405	2.18E+00
Mllt4	-0.372240688	2.17E+00
Dnajb9	-0.289840968	2.16E+00
Tmem98	-0.268665092	2.16E+00
Ubc	0.297018718	2.15E+00
Plcb3	0.371809701	2.15E+00
Nrbp2	0.38773701	2.15E+00
Rpl7a	-0.255207547	2.14E+00
Abat	0.408913315	2.14E+00
Adcyap1r1	0.318387271	2.13E+00
Hmox2	-0.262625903	2.12E+00
Mir124-2hg	0.303111569	2.11E+00
Plat	0.32198072	2.10E+00
Lsamp	0.572914488	2.10E+00
Junb	-1.201706473	2.09E+00
Trove2	0.374740457	2.08E+00
Fgfr1op	0.259127891	2.07E+00
Abca1	0.323349537	2.07E+00
Drc7	0.333388398	2.04E+00
4931429I11Rik	0.269673953	2.04E+00
Idh2	0.312433652	2.04E+00
Fndc3a	-0.382295105	2.03E+00
Slc25a25	-0.485300465	2.03E+00
Gabrg1	0.252499753	2.03E+00
mt-Nd6	0.371345348	2.02E+00
Nipal3	0.250399701	2.01E+00
Ngfrap1	0.394945105	2.01E+00
Tgoln1	-0.404997777	2.01E+00
2700060E02Rik	-0.286957515	2.01E+00
Pkig	0.377314409	2.00E+00
Smoc2	-0.526230357	2.00E+00
Plpp3	0.434258258	2.00E+00
Rasa2	0.345779862	2.00E+00
Ctsh	0.35090276	1.99E+00
Nipbl	0.337914024	1.98E+00
Suclg2	0.377770359	1.98E+00
Ccdc148	0.262136011	1.98E+00
Abcd2	0.259506106	1.97E+00
Tchp	0.258388443	1.96E+00
Ak8	0.308146173	1.96E+00
Adss	0.361774535	1.95E+00
Cox7c	-0.407860004	1.95E+00

Morn1	0.317424578	1.95E+00
Mtss1l	0.365289921	1.95E+00
Gm9780	0.268888123	1.94E+00
Tmem256	-0.321011091	1.94E+00
Ube2e3	-0.260974247	1.93E+00
Lrrc45	0.308358653	1.90E+00
Ntn1	-0.309017149	1.89E+00
Ext2	-0.301953366	1.89E+00
Mpst	0.320073681	1.89E+00
Fzd1	-0.253750274	1.89E+00
Sh3d19	-0.264867837	1.88E+00
Thyn1	-0.393713758	1.88E+00
Padi2	0.307562683	1.85E+00
Anapc1	0.330151252	1.85E+00
Gclc	0.32855111	1.85E+00
Rcc2	0.261911881	1.83E+00
Ier2	-0.480035225	1.83E+00
Snrpd2	0.357051885	1.82E+00
Prdx5	-0.839516364	1.81E+00
Osbpl3	0.264862214	1.80E+00
Wdr73	0.252285239	1.80E+00
Fam216a	0.392269145	1.79E+00
Rapgef3	0.250867982	1.78E+00
Ndufb7	-0.349521629	1.77E+00
Krt18	-0.553363113	1.76E+00
Agt	0.570407817	1.76E+00
Zfp467	0.37834068	1.76E+00
Ift46	0.3581817	1.74E+00
Dgcr2	-0.272142438	1.74E+00
Mdm1	0.254616581	1.74E+00
Tmem17	0.270421901	1.74E+00
Rpl35	-0.301541774	1.73E+00
Arpc3	-0.254326625	1.72E+00
Rnd3	0.32266087	1.71E+00
Nol7	0.353899335	1.70E+00
Nphp3	0.2501827	1.70E+00
Hist1h2bc	0.276837169	1.69E+00
Fbxl2	0.273588895	1.68E+00
Cd59b	0.352370739	1.68E+00
Tacc1	0.354889084	1.67E+00
Nrxn2	0.408433132	1.67E+00
Dbx2	0.516017026	1.66E+00
Fosb	-0.513332189	1.66E+00
Nsrp1	0.3541693	1.66E+00
Pcmt1	0.3095053	1.65E+00
Itpr1	-0.390857275	1.65E+00
Rbbp7	-0.29249021	1.62E+00

Kcne1l	0.260022126	1.61E+00
Fam172a	0.2730295	1.60E+00
Pspc1	0.266086649	1.60E+00
Enkd1	0.253423453	1.60E+00
Pon2	-0.268800373	1.59E+00
Serf2	-0.252734094	1.59E+00
Pfdn5	0.270366047	1.58E+00
Gas2l1	0.253076991	1.57E+00
Mettl9	-0.300190145	1.57E+00
Mok	0.284266139	1.57E+00
Fam117a	-0.271050379	1.56E+00
Hacd3	0.366848066	1.56E+00
Ankrd26	0.277090954	1.55E+00
Slc3a2	-0.338639369	1.55E+00
Aifm3	-0.291718108	1.55E+00
Mboat2	0.301436751	1.54E+00
Mycbp	0.430068274	1.54E+00
Adora2b	0.28356968	1.54E+00
Rhoa	-0.338765241	1.52E+00
Zfhx4	-0.373209668	1.51E+00
Mdh1	-0.705764989	1.51E+00
Map1lc3b	0.314629898	1.50E+00
Trim9	0.251946747	1.48E+00
Gnb2	0.276823798	1.47E+00
Aoc1	0.309444401	1.46E+00
Ttl	0.258837647	1.45E+00
Rbm28	0.348202932	1.43E+00
Rps20	-0.279575779	1.41E+00
Cfdp1	0.37689829	1.39E+00
G3bp2	-0.314298956	1.39E+00
Plekha5	0.283919148	1.38E+00
Ergic1	-0.256867794	1.38E+00
Cry2	0.292084986	1.37E+00
mt-Nd5	0.279472561	1.37E+00
Trap1	0.354419522	1.35E+00
Gapvd1	0.285276982	1.33E+00
Fgfr3	0.425395627	1.32E+00
Myo7a	0.258097764	1.32E+00
Sec61a1	-0.293176014	1.31E+00
Ephx4	0.274498003	1.31E+00
